# Comparison of group 4 and thorium M(iv) substituted cyclopentadienyl silanide complexes†

**DOI:** 10.1039/d3dt00987d

**Published:** 2023-05-12

**Authors:** Benjamin L. L. Réant, Dukula De Alwis Jayasinghe, Ashley J. Wooles, Stephen T. Liddle, David P. Mills

**Affiliations:** a Department of Chemistry, The University of Manchester Oxford Road Manchester M13 9PL U.K stephen.liddle@manchester.ac.uk david.mills@manchester.ac.uk

## Abstract

We report the synthesis and characterisation of a series of M(iv) substituted cyclopentadienyl hypersilanide complexes of the general formula [M(Cp^R^)_2_{Si(SiMe_3_)_3_}(X)] (M = Hf, Th; Cp^R^ = Cp′, {C_5_H_4_(SiMe_3_)} or Cp′′, {C_5_H_3_(SiMe_3_)_2_-1,3}; X = Cl, C_3_H_5_). The separate salt metathesis reactions of [M(Cp^R^)_2_(Cl)_2_] (M = Zr or Hf, Cp^R^ = Cp′; M = Hf or Th, Cp^R^ = Cp′′) with equimolar K{Si(SiMe_3_)_3_} gave the respective mono-silanide complexes [M(Cp′)_2_{Si(SiMe_3_)_3_}(Cl)] (M = Zr, 1; Hf, 2), [Hf(Cp′′)(Cp′){Si(SiMe_3_)_3_}(Cl)] (3) and [Th(Cp′′)_2_{Si(SiMe_3_)_3_}(Cl)] (4), with only a trace amount of 3 presumably formed *via* silatropic and sigmatropic shifts; the synthesis of 1 from [Zr(Cp′)_2_(Cl)_2_] and Li{Si(SiMe_3_)_3_} has been reported previously. The salt elimination reaction of 2 with one equivalent of allylmagnesium chloride gave [Hf(Cp′)_2_{Si(SiMe_3_)_3_}(η^3^-C_3_H_5_)] (5), whilst the corresponding reaction of 2 with equimolar benzyl potassium yielded [Hf(Cp′)_2_(CH_2_Ph)_2_] (6) together with a mixture of other products, with elimination of both KCl and K{Si(SiMe_3_)_3_}. Attempts to prepare isolated [M(Cp^R^)_2_{Si(SiMe_3_)_3_}]^+^ cations from 4 or 5 by standard abstraction methodologies were unsuccessful. The reduction of 4 with KC_8_ gave the known Th(iii) complex, [Th(Cp′′)_3_]. Complexes 2–6 were characterised by single crystal XRD, whilst 2, 4 and 5 were additionally characterised by ^1^H, ^13^C{^1^H} and ^29^Si{^1^H} NMR spectroscopy, ATR-IR spectroscopy and elemental analysis. In order to probe differences in M(iv)–Si bonds for d- and f-block metals we studied the electronic structures of 1–5 by density functional theory calculations, showing M–Si bonds of similar covalency for Zr(iv) and Hf(iv), and less covalent M–Si bonds for Th(iv).

## Abstract 2

The metal-silicon bonds of a series of structurally similar zirconium(iv), hafnium(iv) and thorium(iv) substituted cyclopentadienyl hypersilanide complexes are compared with each other.

## Graphical abstract

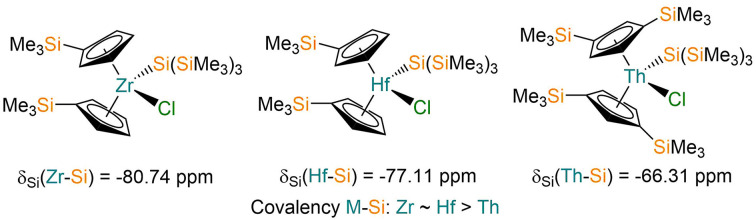

## Introduction

Transition metal (TM) silicon chemistry is well-established,^1^ with applications ranging from homogeneous (hydro)silylation catalysts^2–5^ to TM silicides in catalysis, ceramics and microelectronics.^6^ f-Block silicon chemistry is currently underdeveloped in comparison,^1,7^ but potential uses include lanthanide (Ln) silanide ({SiR_3_}) complexes in olefin polymerisation catalysts,^8,9^ Ln silicides as additives in low-alloy steels,^10^ and high-density actinide (An) silicide nuclear fuels.^11–14^ Given that the physical and chemical properties of Ln and An complexes can differ markedly from each other and related early TM complexes,^15,16^ it follows that comparative studies of their electronic structures and the relative amount of covalency in M–Si bonds are crucial for the development of novel applications.

Since the discovery nearly 40 years ago that group 4 TM bent metallocenes can promote the dehydrogenative polymerisation of silanes,^17^ M(iv) silanide complexes of the general formula [M(Cp^R^)_2_(SiR_3_)(X)] (M = Ti, Zr, Hf; Cp^R^ = substituted cyclopentadienyl, {C_5_R_5_}; X = anion) have been studied extensively;^1^ however, f-block analogues of these complexes have not previously been isolated. As the +4 oxidation state is limited for organometallic Ln complexes^15^ and given the increased radiological hazard and decreased stability of An(iv) complexes across the series,^16^ we targeted a Th(iv) silanide complex for comparison with group 4 M(iv) analogues. Whilst there are >130 structurally authenticated examples of Ti, Zr and Hf complexes containing one or more M–Si bonds, and >90 complexes containing Ln–Si bonds, for An silicon chemistry there are only 1 Th–Si and 6 U–Si bonds reported to date.^18^ Some of us recently reported the synthesis and characterisation of the sole thorium silanide complex, [Th(Cp′)_3_{Si(SiMe_3_)_3_}] (Cp′ = {C_5_H_4_(SiMe_3_)}) and showed by a variety of calculated covalency metrics that the Th–Si bond was less covalent than the corresponding U–Si bond of the isostructural U(iv) complex [U(Cp′)_3_{Si(SiMe_3_)_3_}].^19^ Our previously reported attempt to synthesise the heteroleptic U(iv) complex “[U(Cp′′)_2_{Si(SiMe_3_)_3_}(Cl)]” (Cp′′ = {C_5_H_3_(SiMe_3_)_2_-1,3}) led to metal reduction by the group 1 silanide transfer reagent to give the U(iii) complex [U(Cp′′)_2_{Si(SiMe_3_)_3_}]; this complex showed greater covalency over homologous Ln(iii) complexes [Ln(Cp′′)_2_{Si(SiMe_3_)_3_}] (Ln = La, Ce, Nd), but was less covalent than the group 4 TM(iii) complexes [M(Cp′′)_2_{Si(SiMe_3_)_3_}] (M = Ti, Zr).^20^

Here we report the synthesis of a series of M(iv) substituted cyclopentadienyl hypersilanide complexes of general formula [M(Cp^R^)_2_{Si(SiMe_3_)_3_}(X)] (M = Hf, Th; Cp^R^ = Cp′ or Cp′′; X = Cl or C_3_H_5_) by salt metathesis protocols. All complexes were characterised by single crystal XRD, and for samples with elemental analysis results in accord with expected values we additionally collected ATR-IR and ^1^H, ^13^C{^1^H} and ^29^Si{^1^H} NMR spectra. Density functional theory (DFT) calculations were performed in order to compare the electronic structures of the Hf(iv) and Th(iv) complexes herein with a previously reported Zr(iv) analogue [Zr(Cp′)_2_{Si(SiMe_3_)_3_}(Cl)];^21^ the Th–Si bonds were shown to be less covalent than the Zr–Si and Hf–Si bonds, which showed similar covalency.

## Results and discussion

### Synthesis

**Scheme 1:**
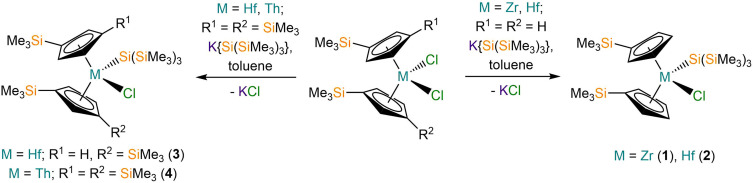
Synthesis of 1–4.

The M(iv) bis-substituted cylopentadienyl dichloride precursors [M(Cp^R^)_2_(Cl)_2_] (M = Zr or Hf, Cp^R^ = Cp′; M = Hf or Th, Cp^R^ = Cp′′) were synthesised by separate salt metathesis reactions of parent [MCl_4_(S)_2_] (M = Zr or Hf, S = THF; M = Th, S = DME) with two equivalents of MCp^R^ (M = Li, K) followed by work-up and recrystallisation, by following literature procedures.^20,22–24^ The separate salt elimination reactions of [M(Cp^R^)_2_(Cl)_2_] with equimolar K{Si(SiMe_3_)_3_}^25^ gave the respective mono-silanide complexes [M(Cp′)_2_{Si(SiMe_3_)_3_}(Cl)] (M = Zr, 1; Hf, 2), [Hf(Cp′′)(Cp′){Si(SiMe_3_)_3_}(Cl)] (3) and [Th(Cp′′)_2_{Si(SiMe_3_)_3_}(Cl)] (4) (Scheme 1). We note that the synthesis of 1 by the salt metathesis reaction of [Zr(Cp′)_2_(Cl)_2_] with Li{Si(SiMe_3_)_3_} has previously been disclosed by Imori *et al.*^21^ Complexes 1, 2 and 4 were obtained in 59–75% yields following work-up and recrystallisation from pentane, whilst 3 formed in <1% crystalline yield, precluding the collection of a full set of characterisation data. We attribute the low yield of 3 to a silyl cleavage reaction occurring, likely by sigmatropic and silatropic shifts, as previously seen in the reactions of MCl_4_ (M = Zr, Hf) with Cp′H and Cp′′H.^26^ This side reaction highlights issues associated with attempting to install two bulky Cp′′ ligands at highly Lewis acidic Zr(iv) and Hf(iv) centres by salt elimination protocols. We were unable to prepare [Th(Cp′)_2_(Cl)_2_] by a salt metathesis reaction of [Th(Cl)_4_(DME)_2_] with two equivalents of LiCp′ due to facile ligand scrambling and the formation of [Th(Cp′)_3_(Cl)];^27^ this precluded the isolation of a Th(iv) hypersilanide complex that is isostructural to 1 and 2 to enable a more direct comparison.

**Scheme 2:**
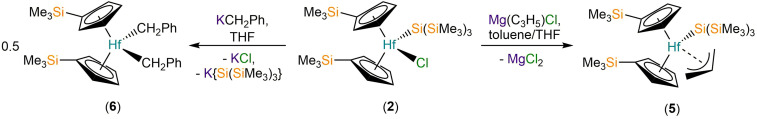
Synthesis of 5 and 6.

In order to install additional synthetic functionality, the salt metathesis reaction of 2 with allylmagnesium chloride in a mixture of toluene and THF was performed to give [Hf(Cp′)_2_{Si(SiMe_3_)_3_}(η^3^-C_3_H_5_)] (5) in 63% yield following work-up and recrystallisation from pentane to remove the MgCl_2_ byproduct (Scheme 2). In contrast, the reaction of 2 with benzyl potassium^28^ in toluene gave several crystals of [Hf(Cp′)_2_(CH_2_Ph)_2_] (6) as part of a mixture of products, presumably by the elimination of both KCl and K{Si(SiMe_3_)_3_} (Scheme 2). We attribute the outcome of the latter reaction to a combination of the low solubility of KCH_2_Ph in toluene, coupled with the proposed intermediate “[Hf(Cp′)_2_{Si(SiMe_3_)_3_}(CH_2_Ph)]” likely being more reactive towards KCH_2_Ph than 2, though we cannot discount ligand scrambling processes occurring. A small-scale reaction of 2 with benzyl potassium in C_6_D_6_ monitored by ^29^Si{^1^H} NMR spectroscopy exhibited a single resonance at −86 ppm after 15 min of reaction time, indicating that a product with a Hf–Si bond is present in the reaction mixture, but this could not be structurally authenticated; the product distribution of this small-scale reaction is likely not representative of the scaled-up version due to differences in solvent, substrate concentration and reaction time.

In efforts to prepare isolated [M(Cp^R^)_2_{Si(SiMe_3_)_3_}]^+^ cations by adapting literature abstraction methodologies, complex 4 was treated with [CPh_3_][Al{OC(CF_3_)_3_}_4_]^29^ and two equivalents of Et_3_SiH (in order to form a silylium cation *in situ* that abstracts Cl^−^ to form strong Si–Cl bonds as an enthalpic driving force)^30^ in benzene, and complex 5 was treated with [NEt_3_H][BPh_4_]^31^ in either toluene or THF (in order to provide an entropic driving force by the formation of propene and trimethylamine *via* a protonolysis reaction). No product could be isolated from the former reaction, whilst no reaction was observed between 5 and the ammonium reagent in both solvent systems investigated. Finally, in an attempt to target the Th(iii) complex “[Th(Cp′′)_2_{Si(SiMe_3_)_3_}]”, which would be structurally analogous to previously reported Ti, Zr, La, Ce, Nd and U analogues,^20^ complex 4 was reacted with 1.2 eq. of KC_8_ ^32^ in DME. Following work-up and recrystallisation from pentane the previously reported homoleptic Th(iii) complex [Th(Cp′′)_3_]^33,34^ was isolated in 59% crystalline yield, presumably by ligand scrambling of “[Th(Cp′′)_2_(X)]” following elimination of KX (X = Cl, {Si(SiMe_3_)_3_}) in accord with the previously reported reduction of [Th(Cp′′)_2_(Cl)_2_] in THF over excess Na/K alloy.^32^

Elemental analysis results obtained for 1, 2, 4 and 5 were in reasonable agreement with expected values, and their ATR-IR spectra (see ESI Fig. S39–S42†) exhibit absorption features that are similar to each other and those previously reported for the closely related M(iii) complexes [M(Cp′′)_2_{Si(SiMe_3_)_3_}] (M = Ti, Zr, La, Ce, Nd, U).^20^ The ATR-IR spectrum of 5 additionally contains the expected resonance at 1531 cm^−1^ for a Hf(iv) η^3^-allyl group, which is at a higher frequency to that previously reported for [Hf(Cp*)(C_4_H_4_BN^i^Pr_2_)(η^3^-C_3_H_5_)] (*ῦ* = 1504 cm^−1^).^35^

### NMR spectroscopy

Complexes 1, 2, 4 and 5 were characterised by ^1^H, ^13^C{^1^H} and ^29^Si{^1^H} NMR spectroscopy (see Table 1 and ESI Fig. S1–S38†). ^1^H and ^13^C{^1^H} NMR chemical shifts were previously reported for 1 ^21^ and the M(iv) precursors [M(Cp^R^)_2_(Cl)_2_],^20,22–24^ but in order to consistently use C_6_D_6_ as the solvent we include these data for [M(Cp′)_2_(Cl)_2_] herein, along with their ^29^Si{^1^H} NMR spectral data for completeness. The spectral assignments were confirmed by correlation experiments for 1, 2, 4 and 5.

**Table 1:** ^1^H, ^13^C{^1^H} and ^29^Si{^1^H} NMR spectral data for [M(Cp^R^)_2_(Cl)_2_], 1, 2, 4 and 5 at 298 K in C_6_D_6_

Complex	Environment	*δ*_H_/ppm	*δ*_C_/ppm	*δ*_Si_/ppm
[Zr(Cp′)_2_(Cl)_2_]^22^	*SiMe*_3_-Cp′	0.33, s, 18H^a^	0.15^a^	−6.40
	3,4-Cp′	5.95, m, 4H^a^	115.36^a^	—
	2,5-Cp′	6.40, m, 4H^a^	126.12^a^	—
	1-Cp′	—	—b	—
[Hf(Cp′)_2_(Cl)_2_]^22^	*SiMe*_3_-Cp′	0.32, s, 18H^a^	0.21^a^	−6.53
	3,4-Cp′	5.89, m, 4H^a^	114.28^a^	—
	2,5-Cp′	6.31, m, 4H^a^	124.94^a^	—
	1-Cp′	—	—b	—
[Hf(Cp′′)_2_(Cl)_2_]^20^	*SiMe*_3_-Cp′′	0.40, s, 36H	0.29	−6.97
	4,5-Cp′′	6.44, m, 4H	119.48	—
	2-Cp′′	7.20, m, 2H	142.34	—
	1,3-Cp′′	—	—b	—
[Th(Cp′′)_2_(Cl)_2_]^24^	*SiMe*_3_-Cp′′	0.37, s, 36H	0.23	−8.83
	4,5-Cp′′	6.95, m, 4H	139.86	—
	2-Cp′′	7.24, m, 2H	140.47	—
	1,3-Cp′′	—	—b	—
1 ^21^	*SiMe*_3_-Cp′	0.26, s, 18H	0.19	−6.13^c^
	3,4-Cp′	5.02, m, 2H; 5.82, m, 2H	111.22, 112.46	—
	2,5-Cp′	6.76, m, 2H; 7.67, m, 2H	114.85, 120.46	—
	1-Cp′	—	129.47	—
	Si(*SiM*e_3_)_3_	0.48, s, 27H	5.22	−6.61^c^
	*Si*(SiMe_3_)_3_	—	—	−80.74^c^
2	*SiMe*_3_-Cp′	0.26, s, 18H	0.23	−6.12
	3,4-Cp′	5.00, m, 2H; 5.87, m, 2H	109.99, 112.59	—
	2,5-Cp′	6.59, m, 2H; 7.50, m, 2H	115.00, 119.07	—
	1-Cp′	—	—b	—
	Si(*SiMe*_3_)_3_	0.49, s, 27H	5.43	−5.66
	*Si*(SiMe_3_)_3_	—	—	−77.11
4	*SiMe*_3_-Cp′′	0.31, s, 18H; 0.34, s, 18H	0.96, 1.18	−7.49, −7.67
	4,5-Cp′′	7.06, m, 2H; 7.46, m, 2H	129.70, 131.12	—
	2-Cp′′	7.32, m, 2H	132.76	—
	1,3-Cp′′	—	140.17, 141.58	—
	Si(*SiMe*_3_)_3_	0.60, s, 27H	6.71	−0.71
	*Si*(SiMe_3_)_3_	—	—	−66.31
5	*SiMe*_3_-Cp′	0.16, s, 18H	0.24	−6.20
	3,4-Cp′	4.88, m, 2H; 5.36, m, 2H	102.69, 104.55	—
	2,5-Cp′	5.62, m, 2H; 6.06, m, 2H	105.10, 110.27	—
	1-Cp′	—	115.23	—
	Si(*SiMe*_3_)_3_	0.52, s, 27H	6.47	−5.29
	*Si*(SiMe_3_)_3_	—	—	−108.82
	CH(*CH*_2_)_2_	2.58, d, 4H, ^3^*J*_HH_ = 12.0 Hz	55.86	—
	*CH*(CH_2_)_2_	4.03, pent, 1H, ^3^*J*_HH_ = 12.0 Hz	112.67	—

a ^1^H and ^13^C{^1^H} NMR data originally reported in CDCl_3_ but recollected here in C_6_D_6_.

b Not observed/obscured by solvent resonance.

c Not reported previously, collected herein.

The ^1^H NMR spectra of [M(Cp′)_2_(Cl)_2_] exhibited three major signals in the expected ratio of 4 : 4 : 18, with typical second order AA′BB′ four spin systems for the two Cp′ ring proton environments and a singlet for the trimethylsilyl groups. The ^1^H NMR spectra of 1 ^21^ and 2 show six major signals in a ratio of 2 : 2 : 2 : 2 : 27 : 18, with two sets of AA′BB′ patterns for the Cp′ ring protons; the inequivalent 2,5- and 3,4-Cp′ ring proton resonances arise from the diminished symmetry in 1 and 2 compared with parent [M(Cp′)_2_(Cl)_2_],^22^ which have mirror planes bisecting the metal and Cp′ centroids. The ^1^H NMR spectrum of 5 shows a similar pattern of signals to 1 and 2, as well as the expected doublet (4H) signal for the methylene and quintet (1H) resonance for the methine of the allyl ligand. This AX_4_ pattern is characteristic of fluxional behaviour, interconverting between η^3^- and η^1^-bound forms faster than the NMR timescale at this temperature; previously reported mono-allyl Hf(iv) complexes can show inequivalent *syn*- and *anti*-methylene groups in an AM_2_X_2_ pattern due to stronger η^3^-binding locking this conformation in solution, *e.g.* [Hf(Cp*)(C_4_H_4_BN^i^Pr_2_)(η^3^-C_3_H_5_)] (Cp* = C_5_Me_5_; *δ*_H_ = 6.99 ppm, m, 1H, C*H*; 3.66 ppm, d, 2H, ^3^*J*_HH_ = 15.4 Hz, C*H*_2_-*anti*; 1.70 ppm, d, 2H, ^3^*J*_HH_ = 9.0 Hz, C*H*_2_-*syn*)^35^ and [Hf(Cp′′)(C_4_H_4_Me_2_-2,3)(η^3^-C_3_H_5_)] (*δ*_H_ = 5.65 ppm, m, 1H, C*H*; 1.64 ppm, m, 2H, C*H*_2_-*syn*; −0.83 ppm, d, 2H, ^3^*J*_HH_ = 8.4 Hz, C*H*_2_-*anti*).^36^ The ^1^H NMR spectrum of 4 differs slightly, with six signals integrating to 2 : 2 : 2 : 27 : 18 : 18; two resonances are observed for the protons at the 4,5-Cp′′ ring positions due to the absence of a mirror plane in 4 compared with parent [Th(Cp′′)_2_(Cl)_2_].^24^ The ^13^C{^1^H} NMR spectra of [M(Cp′)_2_(Cl)_2_], 1 and 2 contain the same number of resonances as their respective ^1^H NMR spectra, with no resonance observed for the quaternary ring carbon atoms; by contrast resonances for all ring carbon environments are present in the ^13^C{^1^H} NMR spectra of 4 and 5. The silyl group resonances in the ^13^C{^1^H} NMR spectra of all complexes studied exhibit satellites from coupling to ^29^Si nuclei, with ^1^*J*_SiC_ ≈ 53 Hz for Cp′/Cp′′ and ^1^*J*_SiC_ ≈ 42 Hz for hypersilanide silyl groups. The allyl group signals in the ^13^C{^1^H} NMR spectrum of 5 are best compared to those of [Hf(Cp′′)(C_4_H_4_Me_2_-2,3)(η^3^-C_3_H_5_)] (*δ*_C_ = 127.87 ppm, *C*H; 57.13 ppm, *C*H_2_).^36^

The ^29^Si{^1^H} NMR spectra of 1, 2, 4 and 5 all exhibit signals for the silyl groups of Cp^R^ rings and the hypersilanide ligands. Most importantly, resonances for the metal-bound silicon atoms were observed for each of these complexes, with the *δ*_Si_ values of structurally analogous 1 (−80.74 ppm) and 2 (−77.11 ppm) being similar to each other and previously reported Zr and Hf hypersilanide complexes, *e.g.* [M(Cp*)(Cp){Si(SiMe_3_)_3_}(Cl)] (M = Zr, *δ*_Si_ = −87.30 ppm;^37^ M = Hf, *δ*_Si_ = −77.87 ppm^38^), [M(Cp)_2_{Si(SiMe_3_)_3_}(Cl)] (M = Zr, *δ*_Si_ = −85.5 ppm; M = Hf, *δ*_Si_ = −79.7 ppm).^39,40^ The substitution of chloride with allyl in 5 leads to a significant upfield shift (*δ*_Si_ = −108.82 ppm), highlighting the sensitivity of silanide chemical shifts to the metal coordination environment. Finally, the *δ*_Si_ value of the metal-bound silicon atom in the Th hypersilanide complex 4 (−66.32 ppm) is significantly downfield to that of the only other known Th(iv) silanide complex, [Th(Cp′)_3_{Si(SiMe_3_)_3_}] (−108.92 ppm).^19^

### Single crystal XRD

**Fig. 1:**
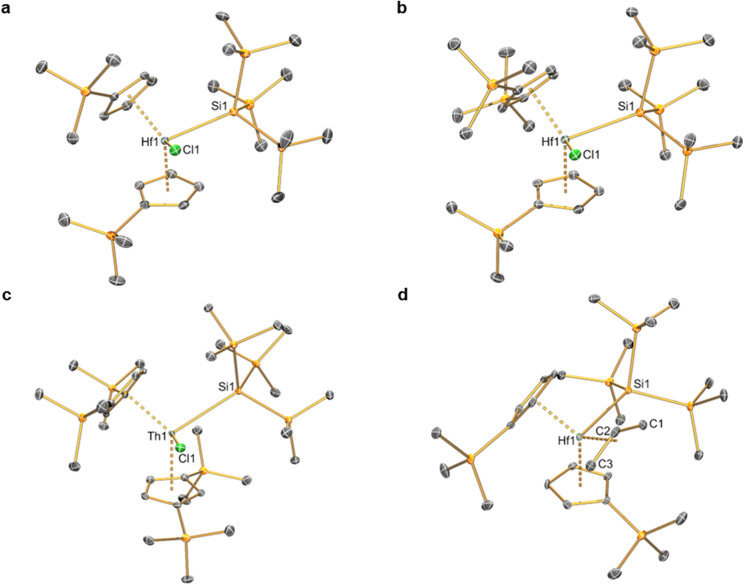
Single crystal XRD structure of (a) [Hf(Cp′)_2_{Si(SiMe_3_)_3_}(Cl)] (2), (b) [Hf(Cp′′)(Cp′){Si(SiMe_3_)_3_}(Cl)]·0.5C_5_H_12_ (3**·0.5C**_**5**_**H**_**12**_), (c) [Th(Cp′′)_2_{Si(SiMe_3_)_3_}(Cl)] (4) and (d) [Hf(Cp′)_2_{Si(SiMe_3_)_3_}(η^3^-C_3_H_5_)] (5) with selective atom labelling. Displacement ellipsoids set at 30% probability level and hydrogen atoms, lattice solvent and modelled disorder components omitted for clarity.

**Fig. 2:**
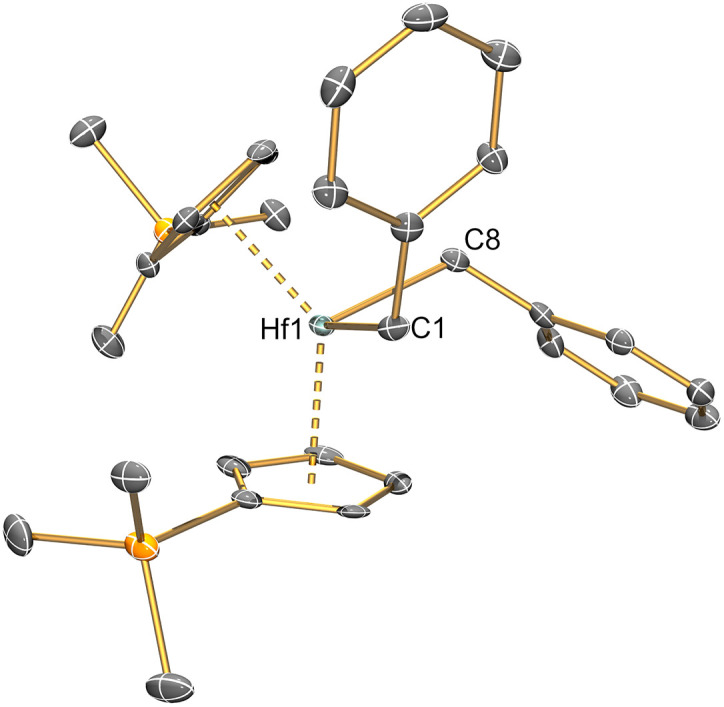
Single crystal XRD structure of [Hf(Cp′)_2_(CH_2_Ph)_2_] (6) with selective atom labelling. Displacement ellipsoids set at 30% probability level and hydrogen atoms omitted for clarity.

Complexes 2–6 and [Hf(Cp′)_2_(Cl)_2_] were studied by single crystal XRD; the solid-state structures of 1 ^21^ and other M(iv) precursors [M(Cp^R^)_2_(Cl)_2_]^20,22–24^ have all been characterised by this method previously (see Fig. 1 and 2 for depictions of 2–6, and Table 2 for selected bond distances and angles; see ESI Fig. S43† for a depiction of [Hf(Cp′)_2_(Cl)_2_], together with Tables S1–S3† for additional crystallographic data). All complexes exhibit bent metallocene geometries with typical M⋯Cp^R^_cent_ distances for the metals and Cp^R^ ligands present;^18^ [Hf(Cp′)_2_(Cl)_2_] exhibits a similar solid-state structure to both its Zr congener^22^ and the related Hf complex [Hf(Cp′′)_2_(Cl)_2_],^20^ and as its metrical parameters are unremarkable we do not comment on this structure further.

**Table 2:** Selected bond lengths (Å), mean M⋯Cp^R^_cent_ distances (Å) and Cp^R^_cent_⋯M⋯Cp^R^_cent_ and Si–M–X angles (°) for 1–6

Parameter	1 ^21^	2	3·0.5C_5_H_12_	4	5	6 a
M–Si	2.833(3)	2.7901(9)	2.8057(8)	3.1053(13)	2.871(2)	—
M–Cl	2.430(2)	2.4041(7)	2.3977(7)	2.6118(11)	—	—
Hf–C^b^	—	—	—	—	2.426(5), 2.501(5), 2.555(5)	2.281(6), 2.268(6)
Mean M⋯Cp^R^_cent_	2.21(1)	2.190(3)	2.198(2)	2.520(4)	2.211(4)	2.198(4)
Si–M–Cl	91.5(1)	91.20(3)	91.10(2)	89.25(6)	—	—
Cp^R^_cent_⋯M⋯Cp^R^_cent_	128.3(4)	128.37(5)	131.70(5)	124.33(12)	127.78(10)	131.68(11)

a Two independent molecules in asymmetric unit, parameters shown for one of these molecules.

b Three allyl Hf⋯C distances for 5 and two Hf–C alkyl bond lengths for 6.

The solid-state structure of 2 is analogous to that of 1, though the bond distances are all shorter in the former complex, as exemplified by the Hf–Si distance (2.7901(9) Å) being shorter than the corresponding Zr–Si bond reported for 1 (2.833(3) Å);^21^ this is in accord with the greater covalent radii of Zr (1.54 Å) *vs*. Hf (1.52 Å).^41^ The structure of 3 is comparable to that of 2, though the additional steric buttressing provided by the additional silyl group in the Cp′′ ring leads to a slightly longer Hf–Si bond (2.8057(8) Å) and a more obtuse Cp^R^_cent_⋯Hf⋯Cp^R^_cent_ angle (131.70(5)° *c.f*. 128.37(5)° for 2). Similarly, the Hf–Si bond (2.871(2) Å) in 5 is elongated due to the additional steric bulk of the allyl ligand compared to the chloride in 2; this distance is still shorter than that previously seen for [Hf(Cp*)(Cp){Si(SiMe_3_)_3_}(Cl)] (2.881(4) Å), due to increased steric buttressing by Cp* *vs*. Cp′′.^38^ The Hf⋯C_allyl_ distances of 5 (range: 2.426(5)–2.555(5) Å) are relatively long in comparison with previously reported Hf allyl complexes,^18^*e.g.* [Hf(Cp′′)(C_4_H_4_Me_2_-2,3)(η^3^-C_3_H_5_)] (Hf⋯C_allyl_ range: 2.409(6)–2.452(6) Å);^36^ this is in accord with the relatively weak binding of the η^3^-allyl in 5 indicated by solution ^1^H NMR studies (see above). The Th–Si distance of 4 (3.1053(13) Å) is shorter than that of [Th(Cp′)_3_{Si(SiMe_3_)_3_}] (3.1191(8) Å),^19^ as expected from the decreased combined steric requirements of one halide and two Cp′′ ligands in the former complex *vs*. the tris-Cp′ motif in the latter. In 1–5 all M–Si distances are longer than the sum of single bond covalent radii for the respective metal and Si (M = Zr, 2.70 Å; Hf, 2.68 Å; Th, 2.91 Å).^41^ The presence of a bulky hypersilanide ligand in 1–5 does not lead to significant changes in M–Cl and M⋯Cp^R^_cent_ distances compared to parent [M(Cp^R^)_2_(Cl)_2_].^20,22–24^ Finally, the mean Hf–C distances (2.275(8) Å) and Hf–C–C_ipso_ angles (121.4(4) and 124.9(4)°) of 6 are comparable to previously reported Hf benzyl complexes,^18^ with the most closely related example being [Hf(Cp)_2_(CH_2_Ph)_2_] (mean Hf–C = 2.293(10) Å, Hf–C–C_ipso_ angles = 120.1(5) and 123.3(5)°).^42^

### Quantum chemical calculations

We performed restricted DFT calculations on 1–6, in order to probe the M–Si and M–C σ-bonds in these complexes further (Table 3). As geometry optimisations effectively reproduced the metrical parameters observed by single crystal XRD we posit that these models are representative of the electronic structures of 1–6; although there are minor variations in the steric bulk provided by ancillary ligands, qualitative comparisons can be made (see ESI Tables S4–S9† for atomic coordinates of geometry-optimised structures). For 1–5 the calculated M–Si distances are longer than those determined experimentally but follow the same trend, with the M–Si bond of 2 shorter than that of 1 and the M–Si bond of 5 being longer than that of 1 and 2, but shorter than the Th–Si bond of 4. The Nalewajski–Mrozek bond indices of 1–3 (range: 0.88–0.89) are essentially identical, with the Th–Si bond of 4 (0.84) lower than these values but higher than the Hf–Si bond of 5 (0.75). The Multipole-Derived Charges (MDC-q) vary markedly for different metals. The *q*_Si_ values for 2, 3 and 5 are consistently −0.16 to −0.17, with higher values for 1 (−0.28) and 4 (−0.68), and whilst the *q*_M_ values are similar for the d-block complexes 1–3 and 5 (range: 0.66–0.77), the *q*_Th_ value for 4 is higher (1.78), showing a higher polarity of M–Si bonds for M = Th *vs*. Zr and Hf. The Hf–Si bond polarity is higher for 5 than for 2 and 3, showing that the allyl ligand is a stronger donor than chloride. The *q*_Hf_ of 6 is 1.15 and the *q*_C_ is −0.99, indicating that there is poorer orbital energy matching between Hf and C than between Hf and Si. The Hf–C bonds in 6 are therefore more polar than the Hf–Si bonds in 2, 3 and 5, but are more covalent than the corresponding Th–Si bond in 4.

**Table 3:** Selected computed DFT, NBO and QTAIM data for the M–Si and M–C σ-bonds in 1–6

Entry^a^	Bond length and index^b^^,^^c^			Charges			Natural bond orbital (NBO) analyses				QTAIM^g^			
	Bond	Bond Length	Bond Index	*q*_M_ d	*q*_Si_ e	*q*_C_ f	M [%]	Si/C [%]	M s/p/d/f [%]	C/Si *n*s/*n*p [%]	*ρ*(*r*)	∇^2^*ρ*(*r*)	*H*(*r*)	*ε*(*r*)
1	Zr–Si	2.8787	0.89	0.66	−0.28	—	32	68	17/0/83/0	36/64	0.05	−0.03	−0.02	0.05
2	Hf–Si	2.8483	0.89	0.69	−0.17	—	29	71	27/0/73/0	37/63	0.05	−0.04	−0.02	0.04
3	Hf–Si	2.8652	0.88	0.71	−0.16	—	28	72	29/0/71/0	37/63	0.05	−0.03	−0.02	0.05
4	Th–Si	3.1759	0.84	1.87	−0.68	—	19	81	34/0/54/12	39/61	0.07	0.07	−0.01	0.01
5	Hf–Si	2.9676	0.75	0.77	−0.17	—	29	71	27/0/73/0	37/63	0.04	0.01	−0.01	0.09
6	Hf–C	2.3232	0.65	1.15	—	−0.99	18	82	27/073/0	23/77	0.08	0.08	−0.04	0.04

a All molecules geometry-optimised without symmetry constraints at the LDA VWN BP86 TZP/ZORA level.

b Calculated bond of interest.

c Nalewajski–Mrozek bond indices.

d Multipole Derived Charges (MDC-q) on M.

e MDC-q charge on Si.

f MDC-q charge on C.

g Quantum Theory of Atoms in Molecules (QTAIM) topological electron density [*ρ*(*r*)], Laplacian [∇^2^*ρ*(*r*)], electronic energy density [*H*(*r*)], and ellipticity [*ε*(*r*)] 3, −1 bond critical point data.

**Fig. 3:**
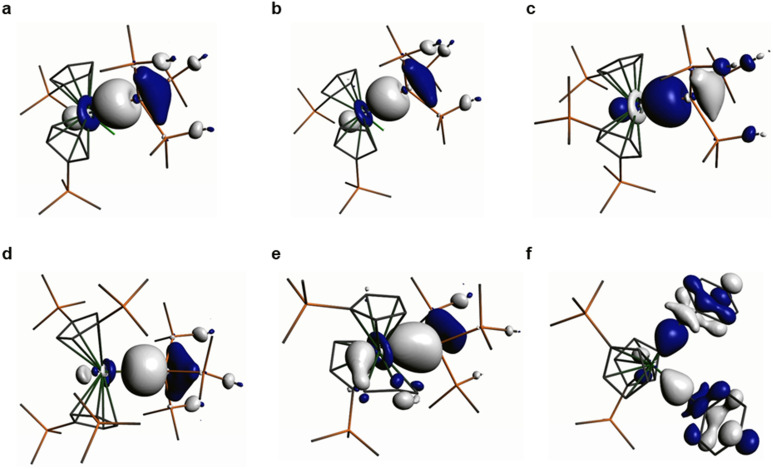
Kohn–Sham Highest Occupied Molecular Orbitals (HOMOs) representing the M–Si or M–C σ-bonds in 1–6. (a) 1 (−4.866 eV); (b) 2 (−4.919 eV); (c) 3 (−4.884 eV); (d) 4 (−4.616 eV); (e) 5 (−4.571 eV); (f) 6 (−4.800 eV). Hydrogen atoms are omitted for clarity.

Natural Bond Orbital (NBO) analyses of the M–Si and M–C bonds in 1–6 show that these are the Highest Occupied Molecular Orbitals (HOMOs) in all cases (see Fig. 3 for Kohn–Sham Molecular Orbital depictions for 1–6, and ESI Fig. S44–S49† for other selected Kohn–Sham Molecular Orbitals and NBOs; N.B. for 6 the HOMO−1 is also a Hf–C σ-bond). The Zr–Si bond in 1 has the most metal character (32%), followed by the Hf–Si bonds in 2, 3 and 5 (28–29%), with the Th–Si bond in 4 showing the least metal contribution (19%) as expected.^16^ The Hf–C bonds of 6 have a lower Hf contribution (18%) than any of the Hf–Si bonds, but these are all remarkably invariant to each other with respect to orbital character (27–29% 6s : 71–73% 5d); the Zr–Si bond in 1 shows a greater metal *n*d-orbital component (17% 5s : 83% 4d), whilst the Th–Si bond in 4 has the most *n*s, the least *n*d, and some 5f contribution (34% 7s : 54% 6d : 12% 5f). The Si orbital contributions to the M–Si bonds in 1–5 do not vary markedly (36–39% 3s : 61–64% 3p), whilst there is a greater *n*p character from C in the Hf–C bonds of 6 (2s 23% : 2p 77%).

Quantum Theory of Atoms in Molecules (QTAIM) analyses of 1–6 reveals highly polarised (range of topological electron densities, *ρ*(*r*): 0.05–0.08) and cylindrical (range of bond ellipticities, *ε*(*r*): 0.01–0.09) M–Si and M–C σ-bonds. Electronic energy densities, *H*(*r*), are all negative as expected and for heavy atoms the Laplacian values (∇^2^*ρ*(*r*), range: 0.04–0.07) are insignificant, as we have commented on previously in DFT analyses of diamagnetic f-block silanide complexes.^19,43,44^ The QTAIM parameters extracted are consistent with the NBO data and bond indices and a visual evaluation of the HOMOs, which all indicate that the Th–Si bond of 4 is less covalent than the Zr–Si bond of 1 and the Hf–Si bonds of 2, 3 and 5. Conversely, the metal-bound silanide resonance in the ^29^Si NMR spectrum of 4 (−66.31 ppm) is downfield to that of 1 (−80.74 ppm) and 2 (−77.11 ppm), which would indicate lower polarity of the Th–Si bond. This discrepancy could be attributed to Heavy Atom on Light Atom (HALA) spin–orbit effects;^45^this is also in-line with the larger ρ(*r*) value of 4 compared to 1–3 perhaps reflecting the more diffuse nature of 6d orbitals, but we note the smaller *H*(*r*) value for 4*vs*. 1–3. A quantitative comparison of the DFT-calculated parameters for 4 with the previously reported Th(iv) hypersilanide complex [Th(Cp′)_3_{Si(SiMe_3_)_3_}] is not possible as these were performed at a different level of theory,^19^ but qualitatively the Th–Si bond of 4 appears to be more polarised and have a lower metal 6d orbital contribution. This highlights that the replacement of a Cp^R^ ligand with a chloride ligand in the Th coordination sphere can have a significant effect on the Th–Si bond, though we caution against making firm conclusions as the calculated Th–Si bond for 4 (3.1759 Å) is 2.3% longer than the distance determined by single crystal XRD (3.1053(13) Å), whereas for [Th(Cp′)_3_{Si(SiMe_3_)_3_}] there was only a 1.6% discrepancy between theory and experiment (3.071 Å calculated *vs*. 3.1191(8) Å measured).^19^

## Conclusion

We have used salt metathesis protocols to synthesise a series of M(iv) hypersilanide complexes for M = Hf and Th, supported by two substituted cyclopentadienyls and one chloride or allyl ancillary ligand, in order to compare their M–Si bonds with each other and previously reported similar Th(iv)^19^ and Zr(iv)^21^ hypersilanide complexes. Attempts to prepare neutral Th(iii) or cationic M(iv) (M = Hf, Th) hypersilanide analogues of these complexes by respective reduction or anion abstraction protocols were unsuccessful under the reaction conditions investigated. However, this work has provided the second structurally authenticated example of a Th–Si bond, and has allowed an extended solid-state structural and ^29^Si{^1^H} NMR spectroscopic comparison of group 4 metal and Th(iv) silanide complexes. We have also shown the importance of the steric bulk of supporting silyl-substituted cyclopentadienyl ligands in dictating the reaction outcome when installing a hypersilanide ligand, due to differences in M(iv) ion size, Lewis acidity and covalency between these metals. DFT calculations of structurally similar M(iv) hypersilanide complexes for M = Zr, Hf and Th confirmed as expected that the highly polarised Th–Si bonds are less covalent than Zr–Si and Hf–Si bonds, which exhibit similar covalency to each other.

## Experimental

### General methods

All manipulations were performed under strict anaerobic conditions using argon as the inert gas, by using a combination of Schlenk line and glove box techniques. HPLC grade pentane, toluene and THF were passed through alumina columns in a solvent purification system. Benzene, 1,4-dioxane and DME were dried by refluxing over potassium for 24 h followed by distillation. All solvents were degassed under dynamic vacuum and were stored over potassium mirrors (benzene pentane, toluene) or 4 Å molecular sieves (1,4-dioxane, DME, THF) before use. C_6_D_6_ was dried by refluxing over potassium for 2 days in a J. Young tap-appended ampoule before trap-to-trap distillation and three freeze–pump–thaw cycles to degas before use. [Zr(Cp′)_2_(Cl)_2_] (M = Zr, Hf),^22^ [M(Cp′′)_2_(Cl)_2_] (M = Hf,^20^ Th^24^), K{Si(SiMe_3_)_3_},^25^ KCH_2_Ph,^28^ [CPh_3_][Al{OC(CF_3_)_3_}_4_],^29^ [NEt_3_H][BPh_4_]^31^ and KC_8_ ^32^ were prepared by previously reported procedures, whilst complex 1 was prepared by adapting the literature method.^21^ Details of the revised synthesis and data obtained for 1 are provided below, together with synthetic procedures and experimental data obtained for 2–6. All other starting materials were purchased from commercial sources and were used as received. **Caution**: *Thorium from natural sources is a weak α-emitter; it is recommended that compounds of this element are only manipulated by trained personnel in designated radiochemical laboratories, and that α-counting equipment is routinely available for monitoring the radiochemical hazard*.

NMR spectra were recorded on samples in J. Young tap-appended NMR tubes using a Bruker Avance III 400 spectrometer operating at 400.07 (^1^H), 100.60 (^13^C) or 79.48 (^29^Si) MHz. ^1^H and ^13^C{^1^H} NMR spectra were internally referenced to the residual solvent resonance of C_6_D_6_, whilst ^29^Si{^1^H} NMR spectra were externally referenced to tetramethylsilane; chemical shifts are provided in ppm. NMR data was analysed using the MestReNova software suite.^46^ ATR-IR spectra of microcrystalline powders were collected on a Bruker Alpha 2 spectrometer. Elemental analyses were carried out by Mr Martin Jennings and Mrs Anne Davies using a Flash 2000 elemental analyser at the Microanalytical service, Department of Chemistry, the University of Manchester. Results generally showed reasonable agreement with expected compositions, though low carbon compositions were reproducibly measured for 4. This is a common occurrence for early metal complexes with high silicon content, including for f-block Cp′′ complexes, where it has been attributed to the formation of silicon carbide, leading to incomplete combustion;^47^ the observation of low carbon values in elemental analyses of [Th(Cp′)_3_{Si(SiMe_3_)_3_}] was previously ascribed to the same phenomenon.^19^

### Crystallographic methods

Crystals of 1–6 were dispersed in fomblin and single crystals were selected and transferred to MicroMounts™ on a goniometer head under a cryostream for exposure to X-rays. Crystallographic data for [Hf(Cp′)_2_(Cl)_2_], 2, 5 and 6 were obtained using an Oxford Diffraction Xcalibur diffractometer equipped with an Agilent Atlas CCD detector with graphite-monochromated Mo Kα (*λ* = 0.71073 Å) radiation, whilst data for 3**·0.5C**_**5**_**H**_**12**_ and 4 were collected using a Rigaku FR-X diffractometer equipped with a HyPix 6000HE photon counting pixel array detector with mirror-monochromated Cu Kα (1.54184 Å) radiation. Intensities were integrated from data recorded on 0.5° (5) or 1° ([Hf(Cp′)_2_(Cl)_2_], 2, 3**·0.5C**_**5**_**H**_**12**_, 4 and 6) frames by ω rotation. Cell parameters were refined from the observed positions of all strong reflections in each data set. A Gaussian grid face-indexed with a beam profile was applied for all structures.^48^ The SHELXT program was used to solve all structures.^49^ Datasets were refined by full-matrix least-squares on all unique *F*^2^ values,^49^ with anisotropic displacement parameters for all non-hydrogen atoms, and with constrained riding hydrogen geometries; *U*_iso_(H) was set at 1.2 (1.5 for methyl groups) times *U*_eq_ of the parent atom. The largest features in final difference syntheses were close to heavy atoms and were of no chemical significance. The CrysAlisPro program^48^ was used for control and integration, and the SHELX suite^49,50^ was employed through the OLEX2 program^51^ for structure solution and refinement. The programs ORTEP-3^52^ and POV-Ray^53^ were used in combination to generate depictions of 1–6.

### Computational methods

The Amsterdam Density Functional (ADF) program (2017 version) was used to perform restricted DFT calculations using standard convergence criteria.^54,55^ Atomic coordinates determined from single crystal XRD were used as the start points for geometry optimisations, which were performed with no constraints applied. Slater-type orbital TZP polarisation all-electron basis sets from the Dirac and zeroth order regular approximation (ZORA) and triple-zeta with polarisation (TZP) database of the Amsterdam Density Functional (ADF) package were used for DFT geometry optimisations. Scalar relativistic approaches (spin–orbit neglected) were used within the ZORA Hamiltonian to include relativistic effects.^56–58^ The local density approximation (LDA) with a correlation potential was used in all calculations.^59^ Generalised gradient approximation corrections were performed using the functionals of Becke and Perdew.^60,61^ NBO analysis was carried out using the NBO v6.0 program.^62^ QTAIM analysis^63,64^ was performed within the ADF suite. The Kohn–Sham MOs and NBOs were visualised using ADFView.

### Synthetic procedures

#### General procedure for the synthesis of [M(Cp^R^)_2_{Si(SiMe_3_)_3_}(Cl)] (1–4)

A Schlenk flask was charged with [M(Cp^R^)_2_(Cl)_2_] (M = Zr, Hf, Cp^R^ = Cp′ = {C_5_H_4_SiMe_3_}; M = Hf, Th, Cp^R^ = Cp′′ = {C_5_H_3_(1,3-SiMe_3_)}), cooled to −78 °C and dissolved in toluene (15 mL mmol^−1^). K{Si(SiMe_3_)_3_} (1 eq.) was dissolved in toluene (10 mL mmol^−1^) and added dropwise to the cooled [M(Cp^R^)_2_(Cl)_2_]. The red (M = Zr) or orange (M = Hf, Th) suspension was warmed to room temperature and stirred for 1 hour. All volatiles were subsequently removed *in vacuo*, and the solids were extracted with pentane (40 mL mmol^−1^). Concentration and storage of solutions at −25 °C led to the formation of needles of [M(Cp^R^)_2_{Si(SiMe_3_)_3_}(Cl)].

#### Preparation of [Zr(Cp′)_2_{Si(SiMe_3_)_3_}(Cl)] (1)

Modified from previous literature method,^21^ prepared according to the general procedure above with [Zr(Cp′)_2_(Cl)_2_] (0.873 g, 2 mmol) and K{Si(SiMe_3_)_3_} (0.574 g, 2 mmol); [Zr(Cp′)_2_{Si(SiMe_3_)_3_}(Cl)] (1) was obtained as red needles (0.792 g, 1.22 mmol, 61%). Anal. Calcd for C_25_H_53_ClSi_6_Zr: C, 46.28; H, 8.23. Found: C, 46.69; H, 8.56. ^1^H NMR (400.07 MHz, C_6_D_6_, 298 K): *δ* = 0.26 (s, 18H, Cp′-Si(C*H*_3_)_3_), 0.48 (s, 27H, Si{Si(C*H*_3_)_3_}_3_), 5.02 (m, 2H, 3,4-Cp′-*H*), 5.82 (m, 2H, 3,4-Cp′-*H*), 6.76 (m, 2H, 2,5-Cp′-*H*), 7.67 (m, 2H, 2,5-Cp′-*H*). ^13^C{^1^H} NMR (100.60 MHz, C_6_D_6_, 298 K): *δ* = 0.19 (^1^*J*_SiC_ = 53.8 Hz, Cp′-Si(*C*H_3_)_3_), 5.22 (^1^*J*_SiC_ = 42.1 Hz, Si{Si(*C*H_3_)_3_}_3_), 111.22 (3,4-Cp′-*C*H), 112.46 (3,4-Cp′-*C*H), 114.85 (2,5-Cp′-*C*H), 120.46 (2,5-Cp′-*C*H), 129.47 (1-Cp′-*C*). ^29^Si{^1^H} NMR (79.48 MHz, C_6_D_6_, 298 K): *δ* = –80.74 (*Si*Zr), −6.61 (Si{*Si*(*C*H_3_)_3_}_3_), −6.13 (Cp′-*Si*(CH_3_)_3_). ATR-IR *ῦ*/cm^−1^: 2949 (m), 2890 (m), 1445 (w), 1406 (m), 1393 (m), 1375 (m), 1245 (s), 1172 (m), 1046 (m), 906 (m), 824 (s), 808 (s), 754 (s), 678 (s), 623 (s), 427 (s).

#### Preparation of [Hf(Cp′)_2_{Si(SiMe_3_)_3_}(Cl)] (2)

Prepared according to the general procedure with [Hf(Cp′)_2_(Cl)_2_] (0.524 g, 1 mmol) and K{Si(SiMe_3_)_3_} (0.287 g, 1 mmol); [Hf(Cp′)_2_{Si(SiMe_3_)_3_}(Cl)] was obtained as orange needles (0.519 g, 0.70 mmol, 70%). Anal. Calcd for C_25_H_53_ClHfSi_6_: C, 40.79; H, 7.26. Found: C, 40.83; H, 7.45. ^1^H NMR (400.07 MHz, C_6_D_6_, 298 K): *δ* = 0.26 (s, 18H, Cp′-Si(C*H*_3_)_3_), 0.49 (s, 27H, Si{Si(C*H*_3_)_3_}_3_), 5.00 (m, 2H, 3,4-Cp′-*H*), 5.87 (m, 2H, 3,4-Cp′-*H*), 6.59 (m, 2H, 2,5-Cp′-*H*), 7.50 (m, 2H, 2,5-Cp′-*H*). ^13^C{^1^H} NMR (100.60 MHz, C_6_D_6_, 298 K): *δ* = 0.23 (^1^*J*_SiC_ = 53.3 Hz, Cp′-Si(*C*H_3_)_3_), 5.43 (^1^*J*_SiC_ = 42.3 Hz, Si{Si(*C*H_3_)_3_}_3_), 109.99 (3,4-Cp′-*C*H), 112.59 (3,4-Cp′-*C*H), 115.00 (2,5-Cp′-*C*H), 119.07 (2,5-Cp′-*C*H), 1-Cp′-*C* not observed. ^29^Si{^1^H} NMR (79.48 MHz, C_6_D_6_, 298 K): *δ* = –77.11 (*Si*Hf), −6.12 (Cp′-*Si*(CH_3_)_3_), −5.66 (Si{*Si*(*C*H_3_)_3_}_3_). ATR-IR *ῦ*/cm^−1^: 2951 (m), 2892 (m), 1438 (w), 1406 (m), 1393 (m), 1376 (m), 1245 (s), 1178 (m), 1046 (m), 908 (m), 818 (s), 812 (s), 754 (s), 678 (s), 623 (s), 427 (s).

#### Preparation of [Hf(Cp′′)(Cp′){Si(SiMe_3_)_3_}(Cl)] (3)

Prepared according to the general procedure with [Hf(Cp′)_2_(Cl)_2_] (0.524 g, 1 mmol) and K{Si(SiMe_3_)_3_} (0.287 g, 1 mmol); [Hf(Cp′′)(Cp′){Si(SiMe_3_)_3_}(Cl)] was obtained as orange needles in <1% crystalline yield, precluding the collection of additional characterisation data.

#### Preparation of [Th(Cp′′)_2_{Si(SiMe_3_)_3_}(Cl)] (4)

Prepared according to the general procedure with [Th(Cp′′)_2_(Cl)_2_] (1.805 g, 2.5 mmol) and K{Si(SiMe_3_)_3_} (0.717 g, 2.5 mmol); 4 was obtained as orange needles (1.756 g, 1.88 mmol, 75%). Anal. Calcd for C_31_H_69_ClSi_8_Th: C, 39.86; H, 7.45. Found: C, 38.89; H, 7.51. ^1^H NMR (400.07 MHz, C_6_D_6_, 298 K): *δ* = 0.31 (s, 18H, Cp′′′-Si(C*H*_3_)_3_), 0.34 (s, 18H, Cp′′-Si(C*H*_3_)_3_), 0.60 (s, 27H, Si{Si(C*H*_3_)_3_}_3_), 7.06 (m, 2H, 4,5-Cp′′-*H*), 7.32 (m, 2H, 2-Cp′′-*H*), 7.46 (m, 2H, 4,5-Cp′′-*H*). ^13^C{^1^H} NMR (100.60 MHz, C_6_D_6_, 298 K): *δ* = 0.96 (^1^*J*_SiC_ = 52.7 Hz, Cp′′-Si(*C*H_3_)_3_), 1.18 (^1^*J*_SiC_ = 52.1 Hz, Cp′′-Si(*C*H_3_)_3_), 6.71 (^1^*J*_SiC_ = 42.6 Hz, Si{Si(*C*H_3_)_3_}_3_), 129.70 (4,5-Cp′′-*C*H), 131.12 (4,5-Cp′′-*C*H), 132.76 (2-Cp′′-*C*H), 140.17 (1,3-Cp′′-*C*), 141.58 (1,3-Cp′′-*C*). ^29^Si{^1^H} NMR (79.48 MHz, C_6_D_6_, 298 K): *δ* = –66.32 (*Si*Th), −7.67 (Cp′′-*Si*(CH_3_)_3_), −7.50 (Cp′′-*Si*(CH_3_)_3_), −0.71 (Si{*Si*(*C*H_3_)_3_}_3_). ATR-IR *ῦ*/cm^−1^: 2951 (m, C–H stretch), 2894 (w, C–H stretch), 1436 (w), 1407 (w), 1243 (s), 1077 (s), 1021 (m), 917 (m), 820 (s), 797 (s), 750 (s), 691 (s), 678 (s), 637 (s), 619 (s), 469 (s), 413 (m).

#### Preparation of [Hf(Cp′)_2_{Si(SiMe_3_)_3_}(η^3^-C_3_H_5_)] (5)

A solution of 2.0 M Mg(C_3_H_5_)Cl in THF (0.6 mL, 1.2 mmol) was added dropwise *via* a glass syringe with a stainless steel Luer lock needle to a Schlenk flask containing a solution of 2 (0.736 g, 1 mmol) in toluene (20 mL). The yellow reaction mixture was allowed to stir overnight. Volatiles were removed *in vacuo* and pentane (30 mL) and 1,4-dioxane (3 mL) was added and the reaction mixture was stirred for 1 h in order to form MgCl_2_·1,4-dioxane. Volatiles were removed *in vacuo* to give a bright yellow powder, which was extracted with pentane (40 mL). Filtration, concentration and storage of the resultant bright yellow solution at −25 °C led to the formation of yellow blocks of 5 (0.466 g, 0.63 mmol, 63%). Anal. Calcd for C_28_H_58_ClHfSi_6_: C, 45.34; H, 7.88. Found: C, 45.04; H, 8.16. ^1^H NMR (400.07 MHz, C_6_D_6_, 298 K): *δ* = 0.16 (s, 18H, Cp′-Si(C*H*_3_)_3_), 0.52 (s, 27H, Si{Si(C*H*_3_)_3_}_3_), 2.58 (d, 4H, ^3^*J*_HH_ = 12.0 Hz, CH(C*H*_2_)_2_), 4.03 (pent, 1H, ^3^*J*_HH_ = 12.0 Hz, C*H*(CH_2_)_2_), 4.88 (m, 2H, 3,4-Cp′-*H*), 5.36 (m, 2H, 3,4-Cp′-*H*), 5.62 (m, 2H, 2,5-Cp′-*H*), 6.06 (m, 2H, 2,5-Cp′-*H*). ^13^C{^1^H} NMR (100.60 MHz, C_6_D_6_, 298 K): *δ* = 0.24 (^1^*J*_SiC_ = 53.3 Hz, Cp′-Si(*C*H_3_)_3_), 6.47 (^1^*J*_SiC_ = 41.1 Hz, Si{Si(*C*H_3_)_3_}_3_), 55.86 (CH(*C*H_2_)_2_), 102.69 (3,4-Cp′-*C*H), 104.55 (3,4-Cp′-*C*H), 105.10 (2,5-Cp′-*C*H), 110.27 (2,5-Cp′-*C*H), 112.67 (*C*H(CH_2_)_2_), 115.23 (1-Cp′-*C*). ^29^Si{^1^H} NMR (79.48 MHz, C_6_D_6_, 298 K): *δ* = –108.82 (*Si*Hf), −6.20 (Cp′-*Si*(CH_3_)_3_), −5.29 (Si{*Si*(*C*H_3_)_3_}_3_). ATR-IR *ῦ*/cm^−1^: 2951 (m, C–H stretch), 2897 (w, C–H stretch), 1531 (w, allyl stretch) 1445 (w), 1405 (w), 1376 (w), 1315 (w), 1241 (s), 1167 (m), 1044 (m), 904 (m), 824 (s), 799 (s), 752 (s), 667 (s), 620 (s), 423 (m).

#### Preparation of [Hf(Cp′)_2_(CH_2_Ph)_2_] (6)

Toluene (10 mL) was added *via* a stainless steel cannula to a Schlenk flask charged with 2 (0.368 g, 0.5 mmol) and KCH_2_Ph (0.065 g, 0.5 mmol) in toluene (20 mL). The orange reaction mixture was allowed to stir for 2 h. Volatiles were removed *in vacuo* to give an orange powder, which was extracted with pentane (25 mL). Filtration, concentration and storage of the resultant orange solution at −25 °C led to the formation of yellow blocks of 6 together with other reaction products, precluding the collection of additional characterisation data.

## Conflicts of interest

The authors declare no conflicts of interest.

## Supplementary Material

DT-052-D3DT00987D-s001

DT-052-D3DT00987D-s002
