# Supplementary material for: Comparison of group 4 and thorium M(iv) substituted cyclopentadienyl silanide complexes
Source: Dalton Trans. 2023 May 12;52(22):7635–45. doi: 10.1039/d3dt00987d (PMC10242453; doi:10.1039/d3dt00987d)
Supplement: DT-052-D3DT00987D-s001 [file DT-052-D3DT00987D-s001.pdf]

Electronic Supplementary Material (ESI) for Dalton Transactions.
This journal is © The Royal Society of Chemistry 2023

- Electronic Supplementary Information -

Comparison of group 4 and thorium M(IV) substituted cyclopentadienyl silanide complexes

Benjamin L. L. Réant, Dukula De Alwis Jayasinghe, Ashley J. Wooles, Stephen T. Liddle,* and David P. Mills*

Department of Chemistry, The University of Manchester, Oxford Road, Manchester, M13 9PL, U.K.

*stephen.liddle@manchester.ac.uk; david.mills@manchester.ac.uk.

Contents

1. NMR spectroscopy S1

1.1. ^{1}H NMR spectra [M(Cp′)$_2$(Cl)$_2$] (M = Zr, Hf), 1, 2, 4 and 5 .... S2

1.2. ^{13}C{^{1}H} NMR spectra of [M(Cp′)$_2$(Cl)$_2$] (M = Zr, Hf), 1, 2, 4 and 5 .... S8

1.3. ^{29}Si{^{1}H} NMR spectra of [M(Cp′)$_2$(Cl)$_2$] (M = Zr, Hf), 1, 2, 4 and 5 .... S18

1.4. 2D NMR spectra of 1, 2, 4 and 5 .... S24

2. ATR-IR spectroscopy S40

3. Crystallographic data S42

4. NBO representations of selected frontier orbitals of 1-6 .... S46

5. Optimised geometry coordinates for 1-6 .... S49

1. NMR spectroscopy

1.1. ^{1}H NMR spectra of $[M(Cp')_2(Cl)_2]$ (M = Zr, Hf), 1, 2, 4 and 5

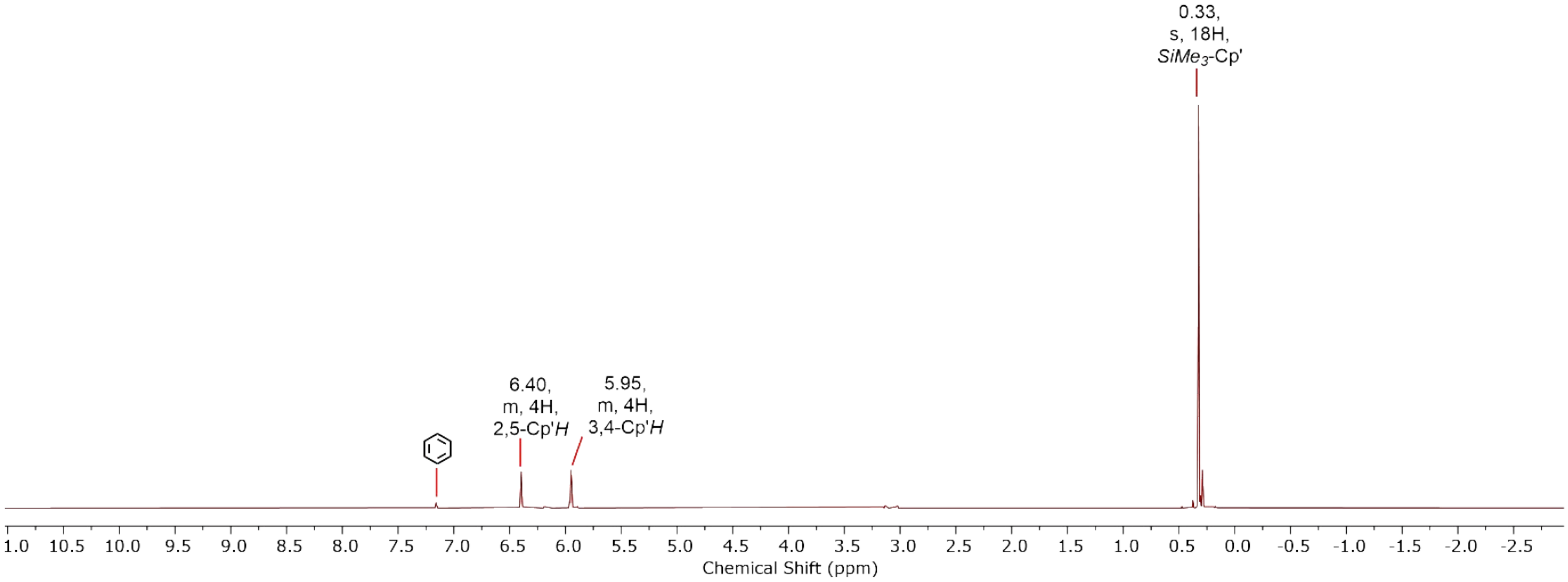

Figure S1. ^{1}H NMR spectrum of $[Zr(Cp')_2(Cl)_2]$ in d_6-benzene.

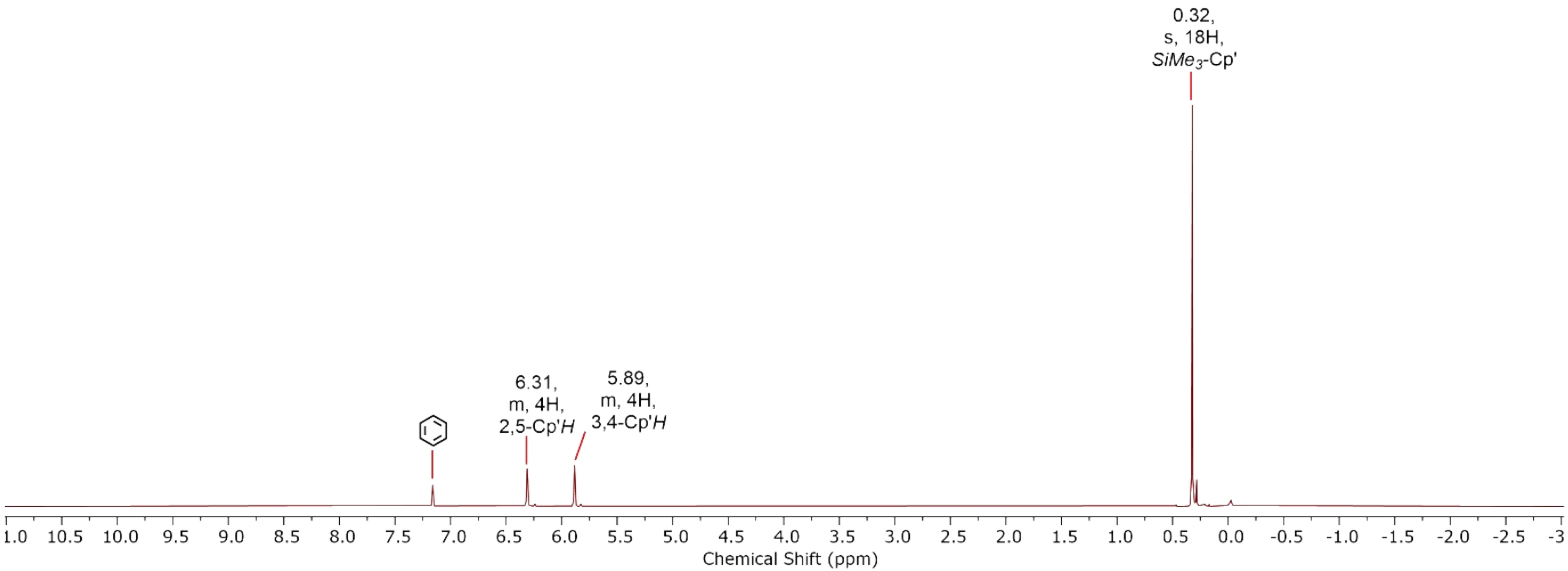

Figure S2. ^{1}H NMR spectrum of [Hf(Cp′)$_2$(Cl)$_2$] in d_6-benzene.

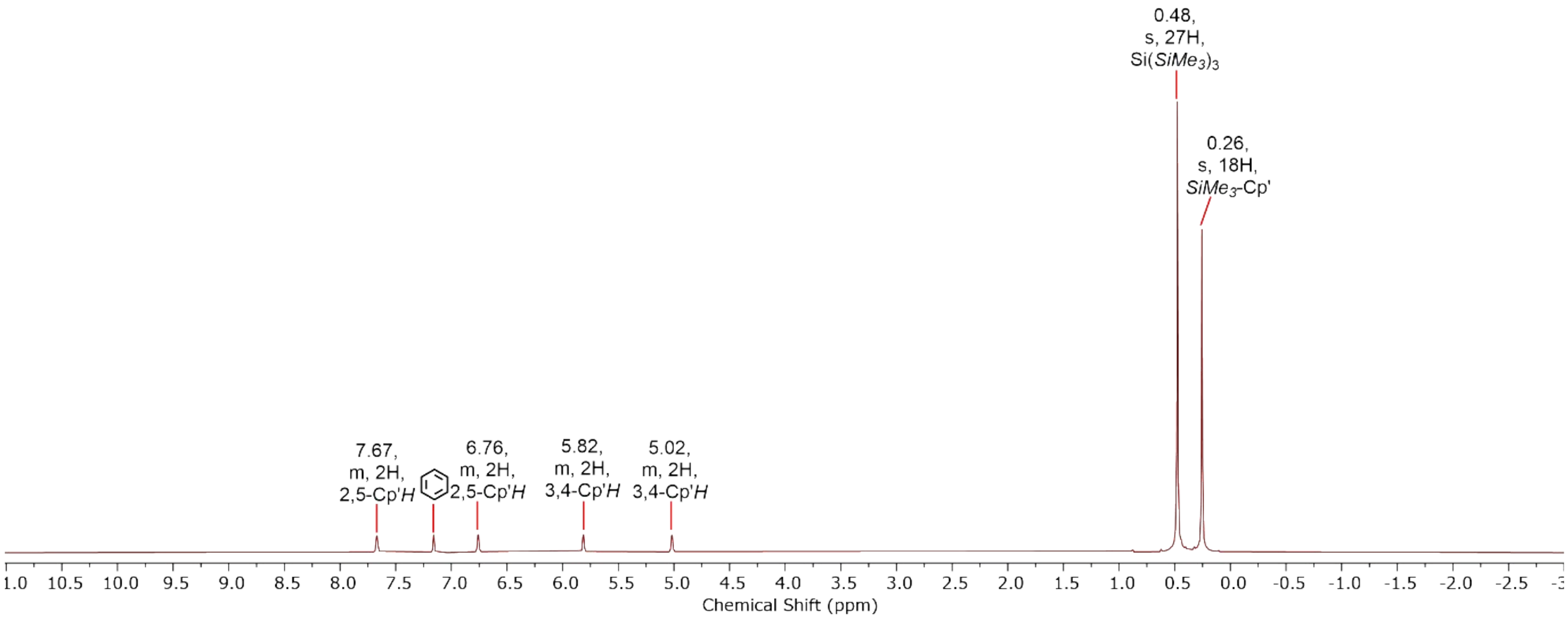

Figure S3. ^{1}H NMR spectrum of **1** in d_6-benzene.

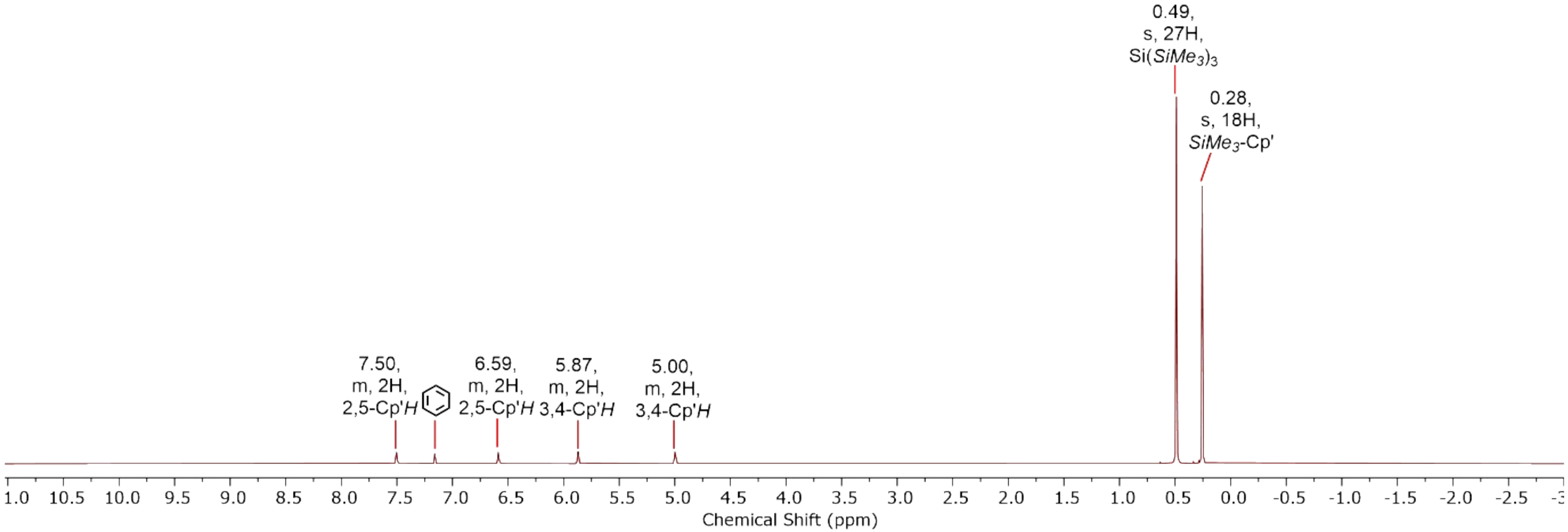

Figure S4. ^{1}H NMR spectrum of **2** in d_6-benzene.

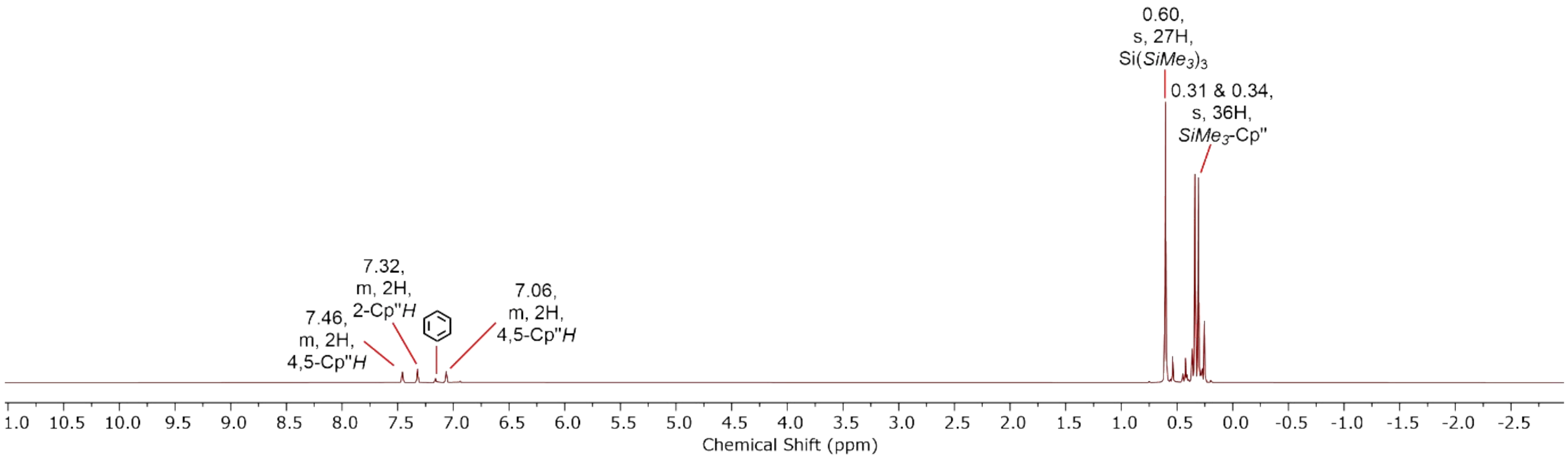

Figure S5. ^{1}H NMR spectrum of **4** in d_6-benzene.

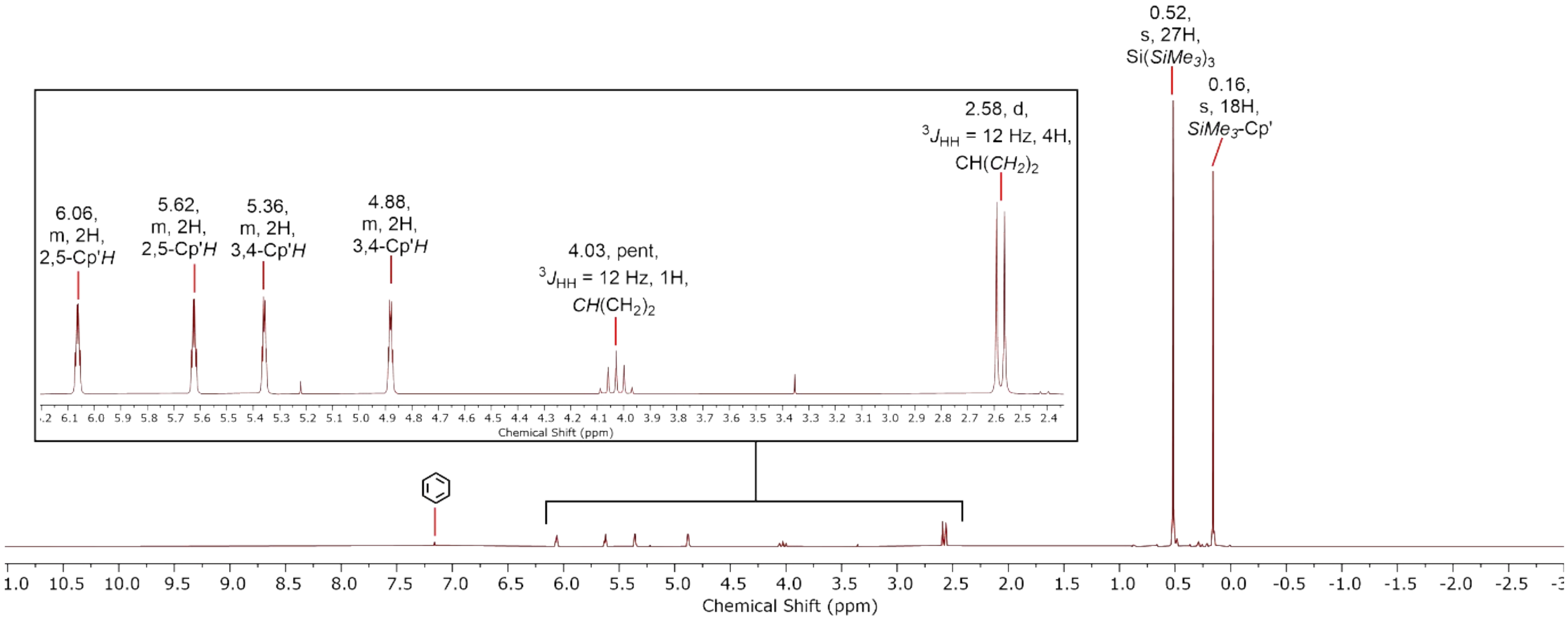

Figure S6. ^{1}H NMR spectrum of **5** in d_6-benzene.

1.2. $^{13}C\{^1H\}$ NMR spectra of [M(Cp′)$_2$(Cl)$_2$] (M = Zr, Hf), 1, 2, 4 and 5

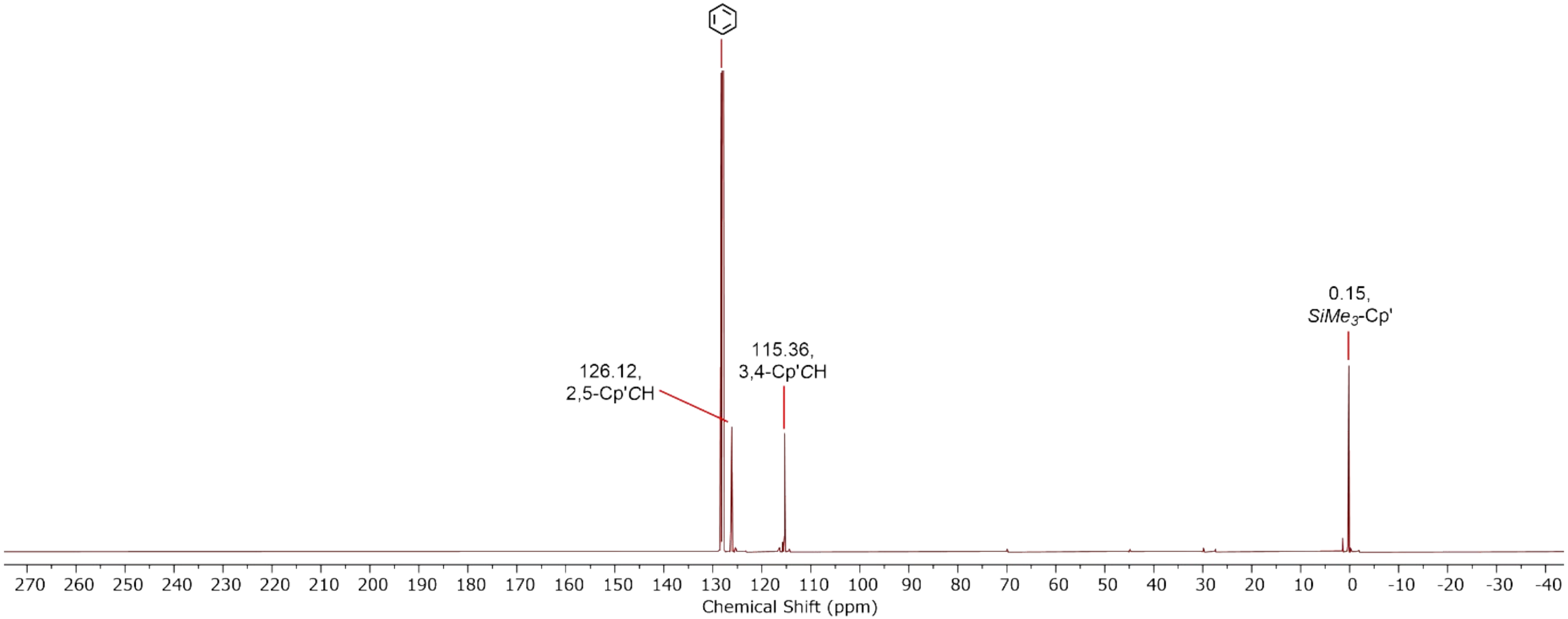

Figure S7. $^{13}C\{^1H\}$ NMR spectrum of [Zr(Cp′)$_2$(Cl)$_2$] in d_6-benzene.

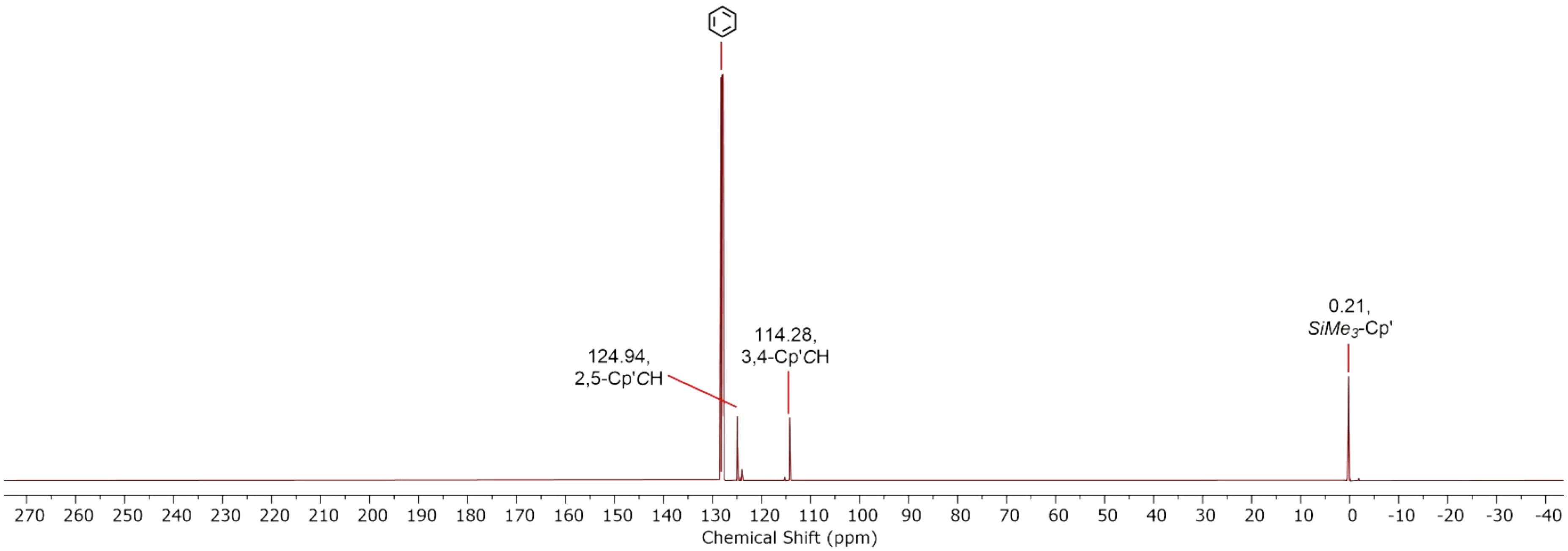

Figure S8. $^{13}C\{^{1}H\}$ NMR spectrum of $[Hf(Cp')_2(Cl)_2]$ in d_6-benzene.

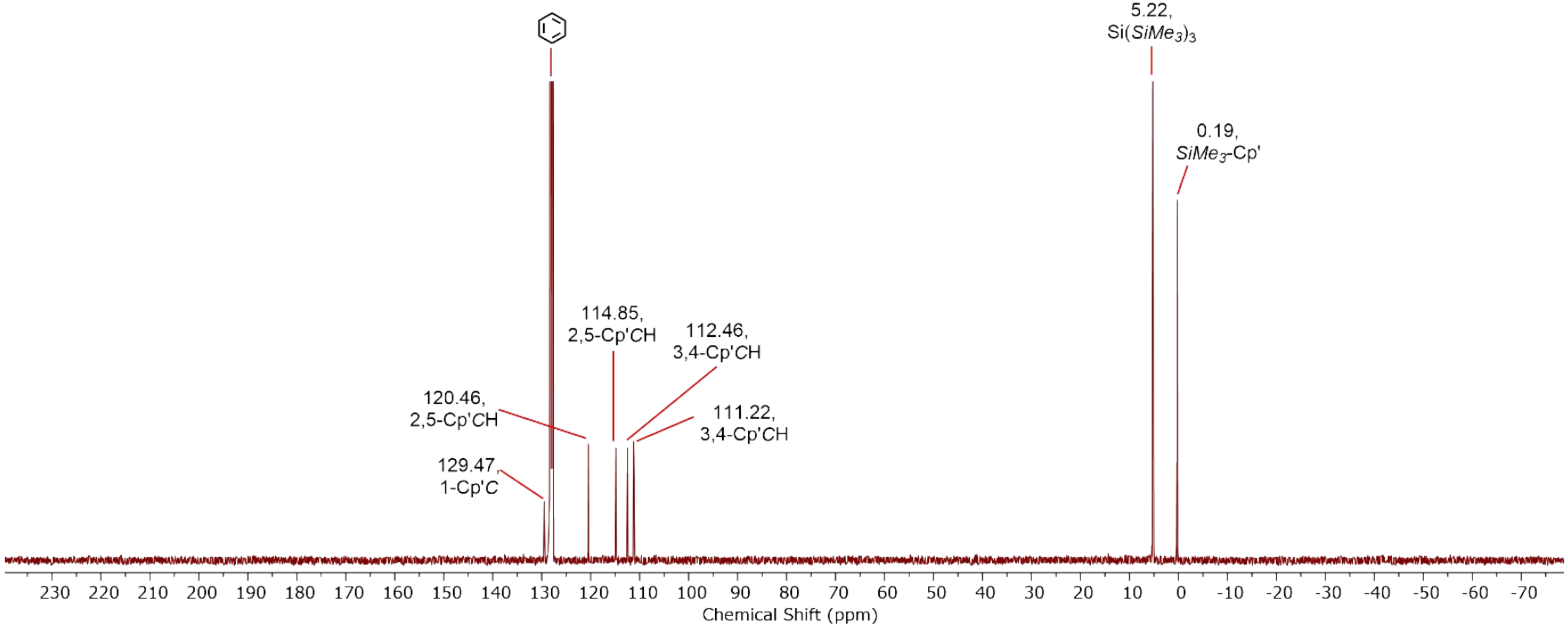

Figure S9. $^{13}C\{^1H\}$ NMR spectrum of **1** in d_6-benzene.

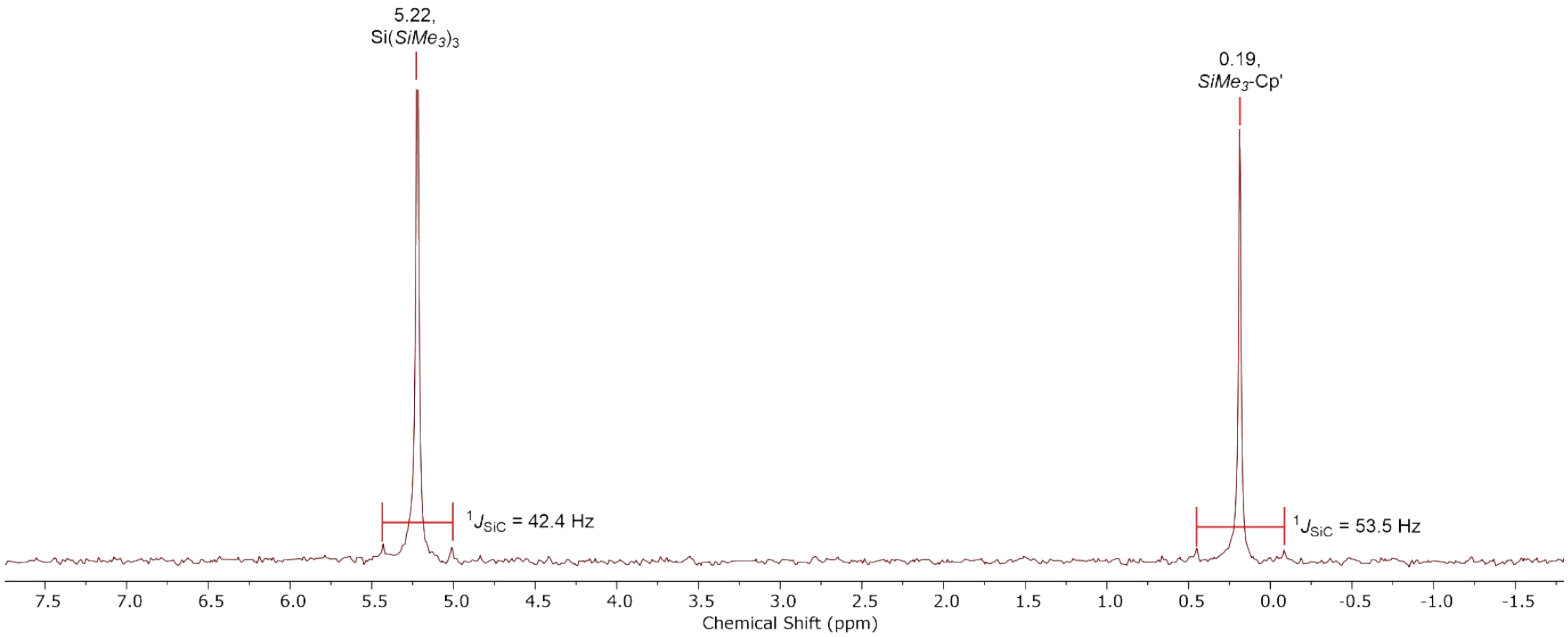

Figure S10. Zoomed in silyl region $^{13}C\{^1H\}$ NMR spectrum of **1** in d_6-benzene.

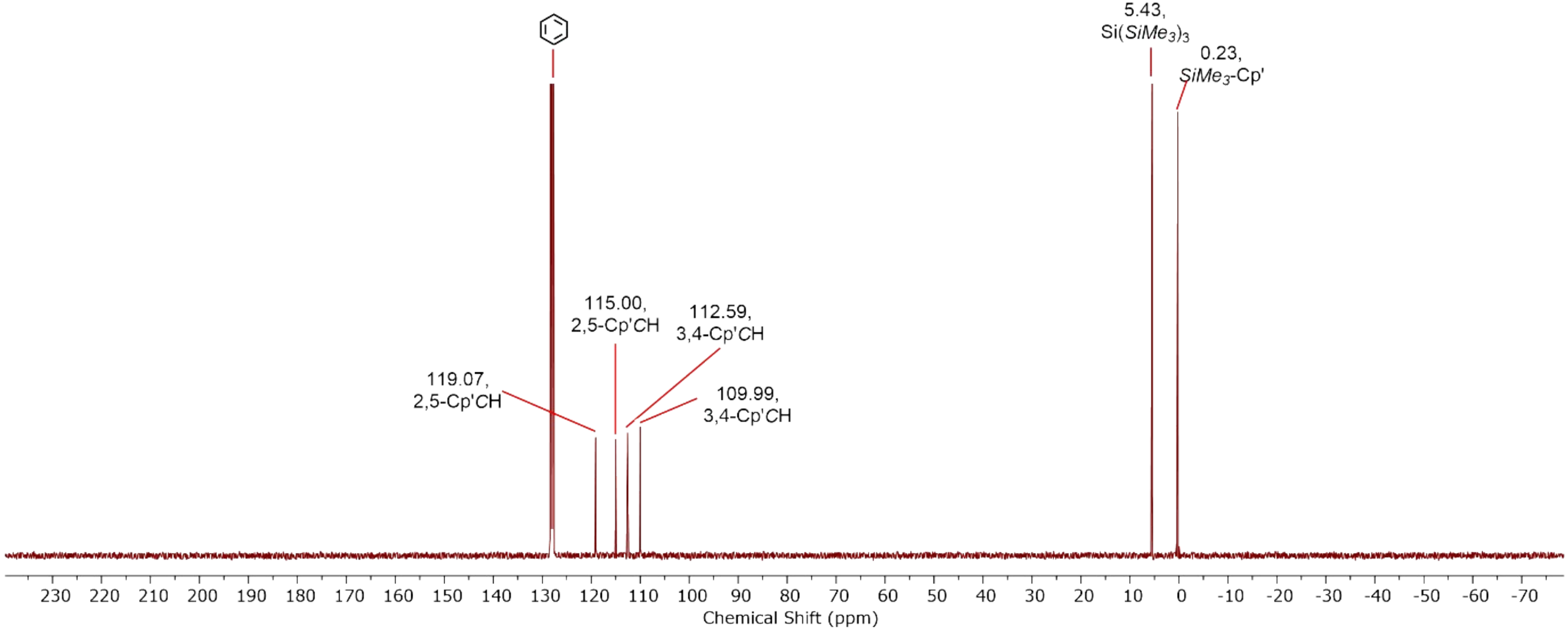

Figure S11. $^{13}C\{^{1}H\}$ NMR spectrum of **2** in d_6-benzene.

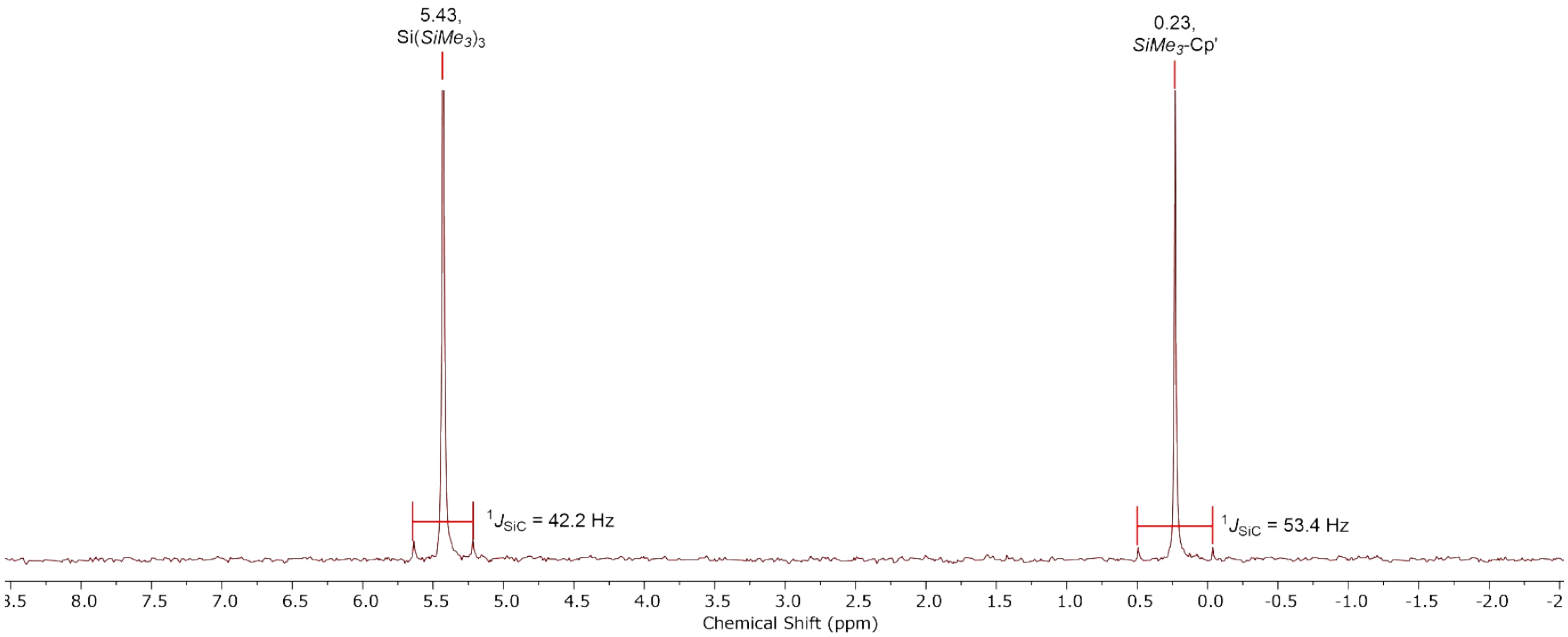

Figure S12. Zoomed in silyl region $^{13}C\{^1H\}$ NMR spectrum of **2** in d_6-benzene.

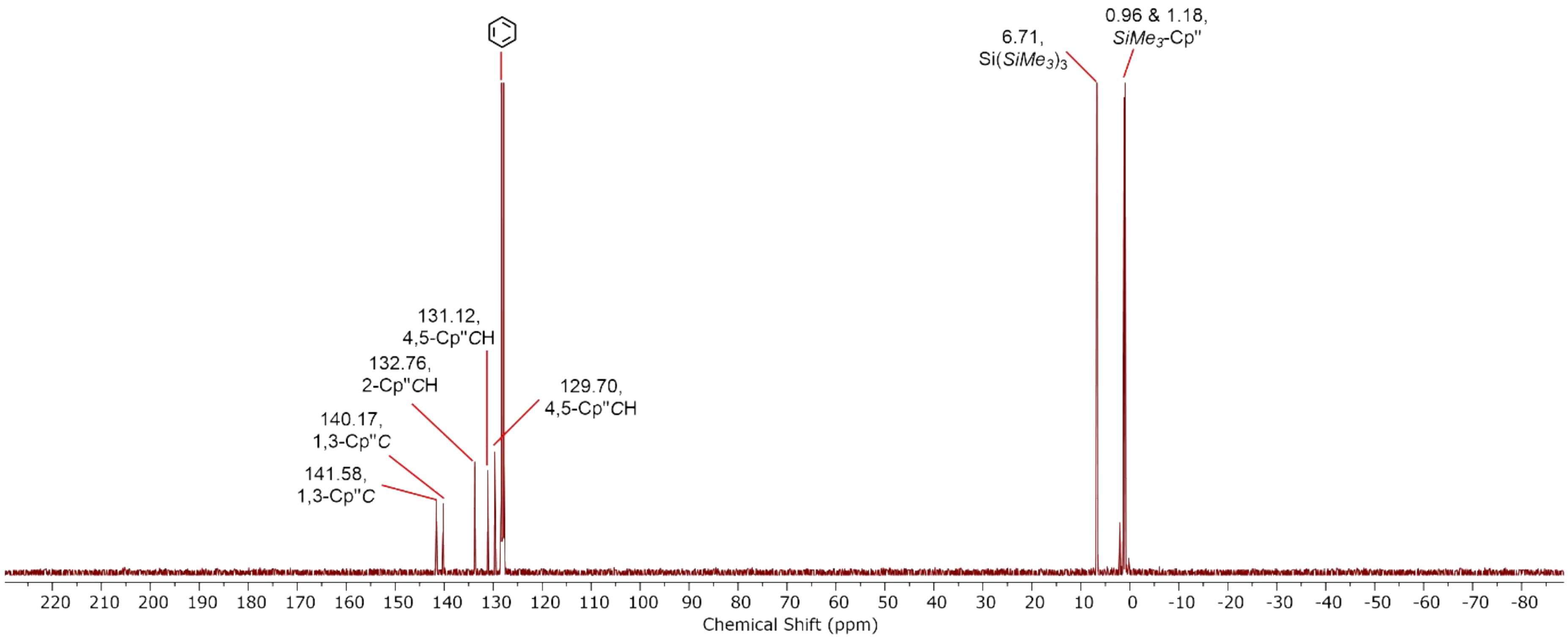

Figure S13. $^{13}C\{^1H\}$ NMR spectrum of **4** in d_6-benzene.

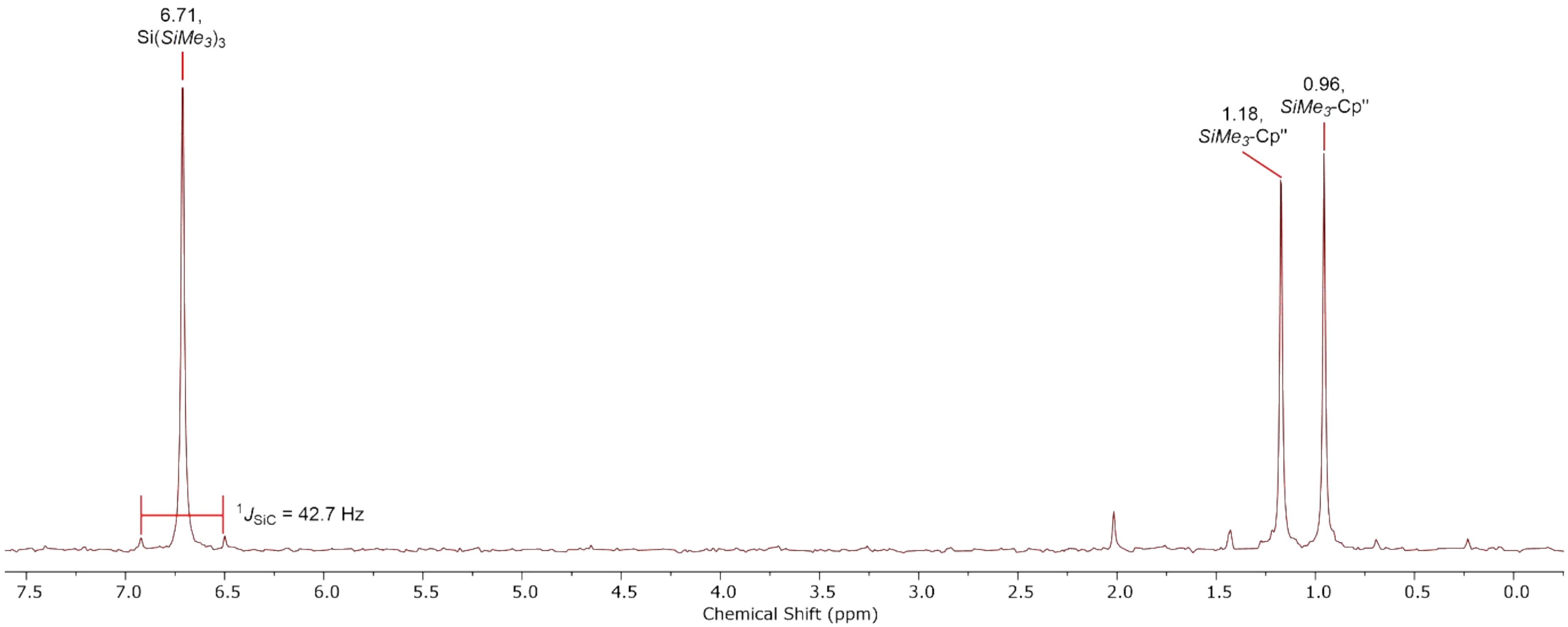

Figure S14. Zoomed in silyl region $^{13}C\{^{1}H\}$ NMR spectrum of **4** in d_6-benzene.

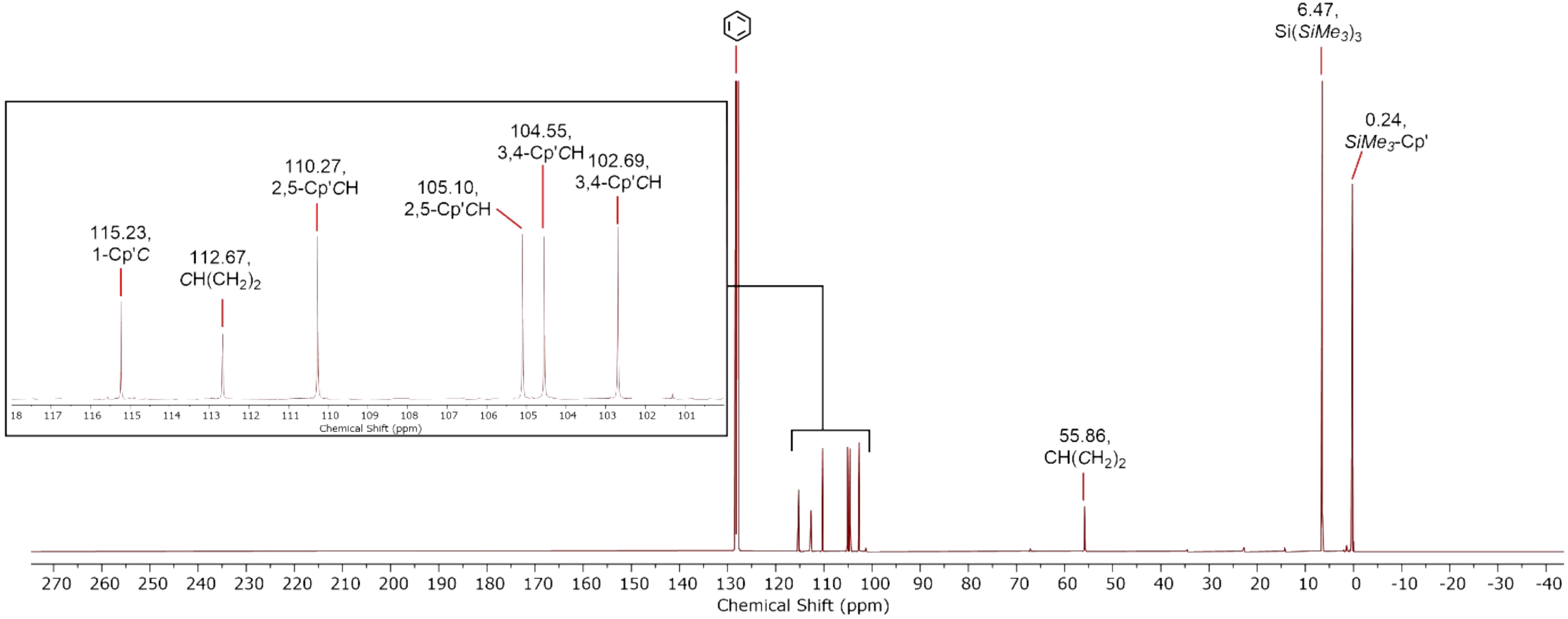

Figure S15. $^{13}C\{^1H\}$ NMR spectrum of **5** in d_6-benzene.

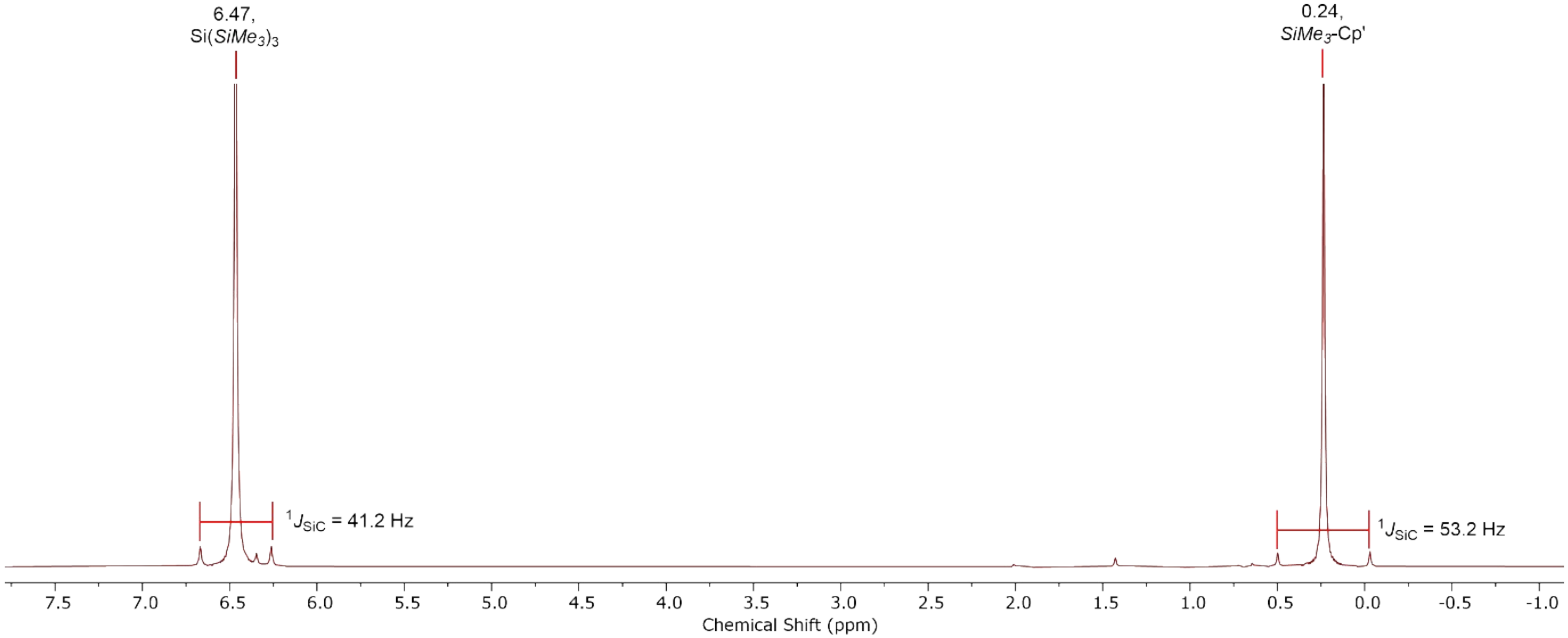

Figure S16. Zoomed in silyl region $^{13}C\{^1H\}$ NMR spectrum of **5** in d_6-benzene.

1.3. $^{29}Si\{^1H\}$ NMR spectra of $[M(Cp')_2(Cl)_2]$ (M = Zr, Hf), 1, 2, 4 and 5

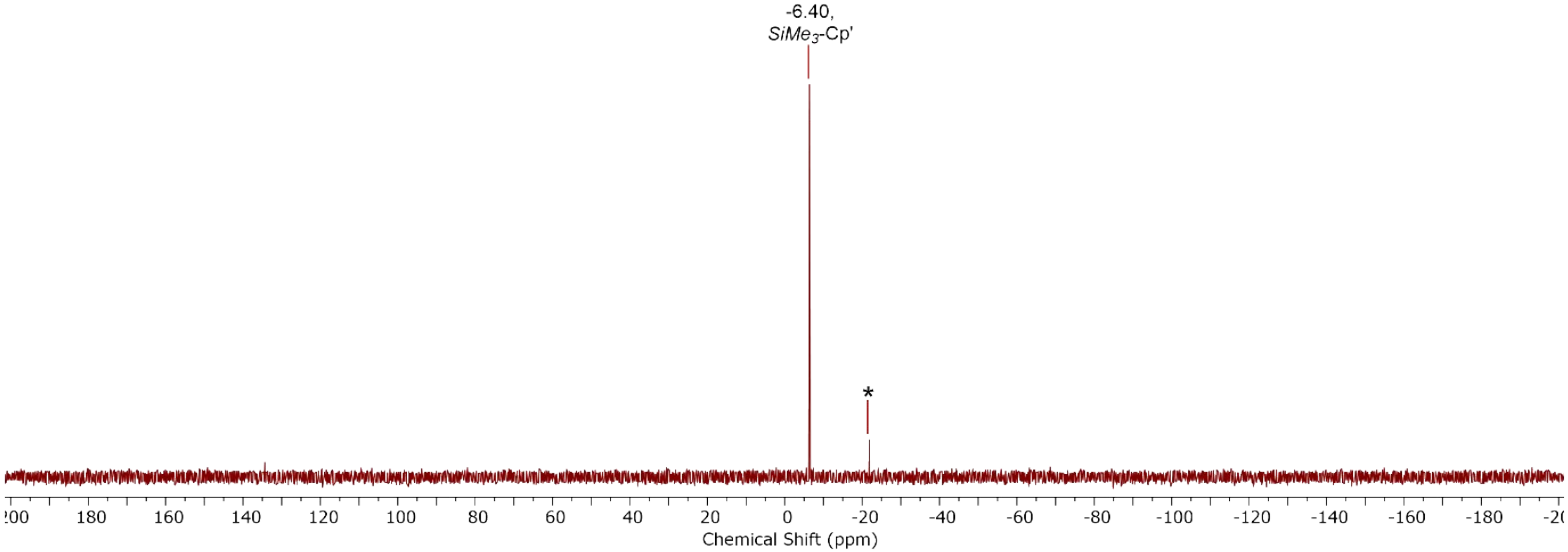

Figure S17. $^{29}Si\{^1H\}$ NMR spectrum of $[Zr(Cp')_2(Cl)_2]$ in d_6-benzene. * denotes silicon grease.

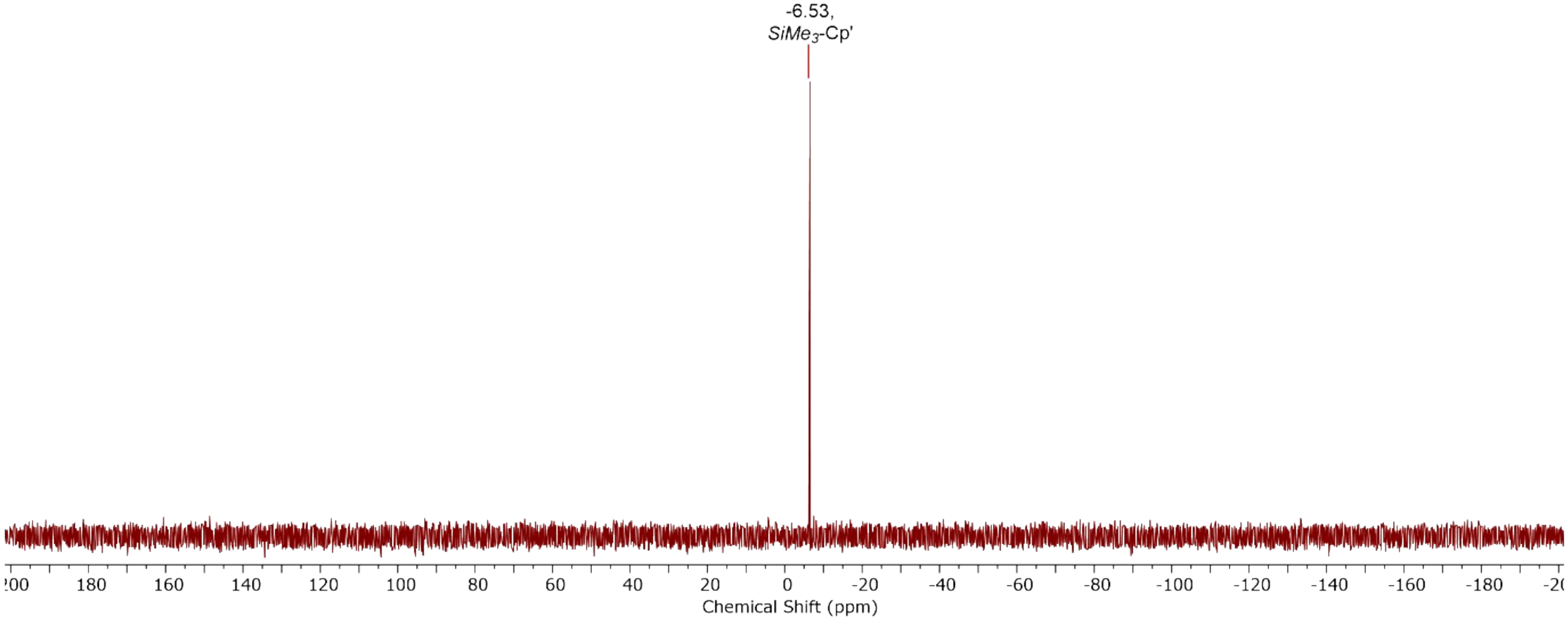

Figure S18. $^{29}Si\{^{1}H\}$ NMR spectrum of $[Hf(Cp')_2(Cl)_2]$ in d_6-benzene.

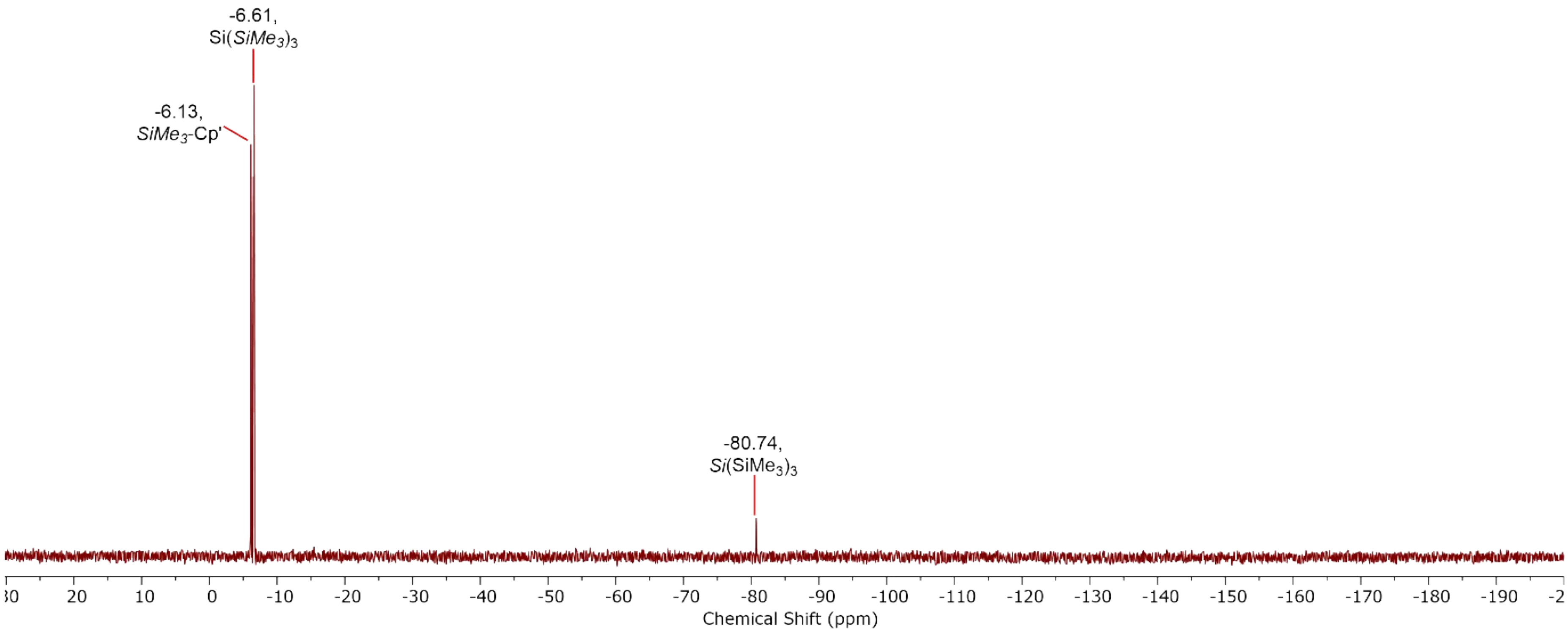

Figure S19. $^{29}Si\{^{1}H\}$ NMR spectrum of **1** in d_6-benzene.

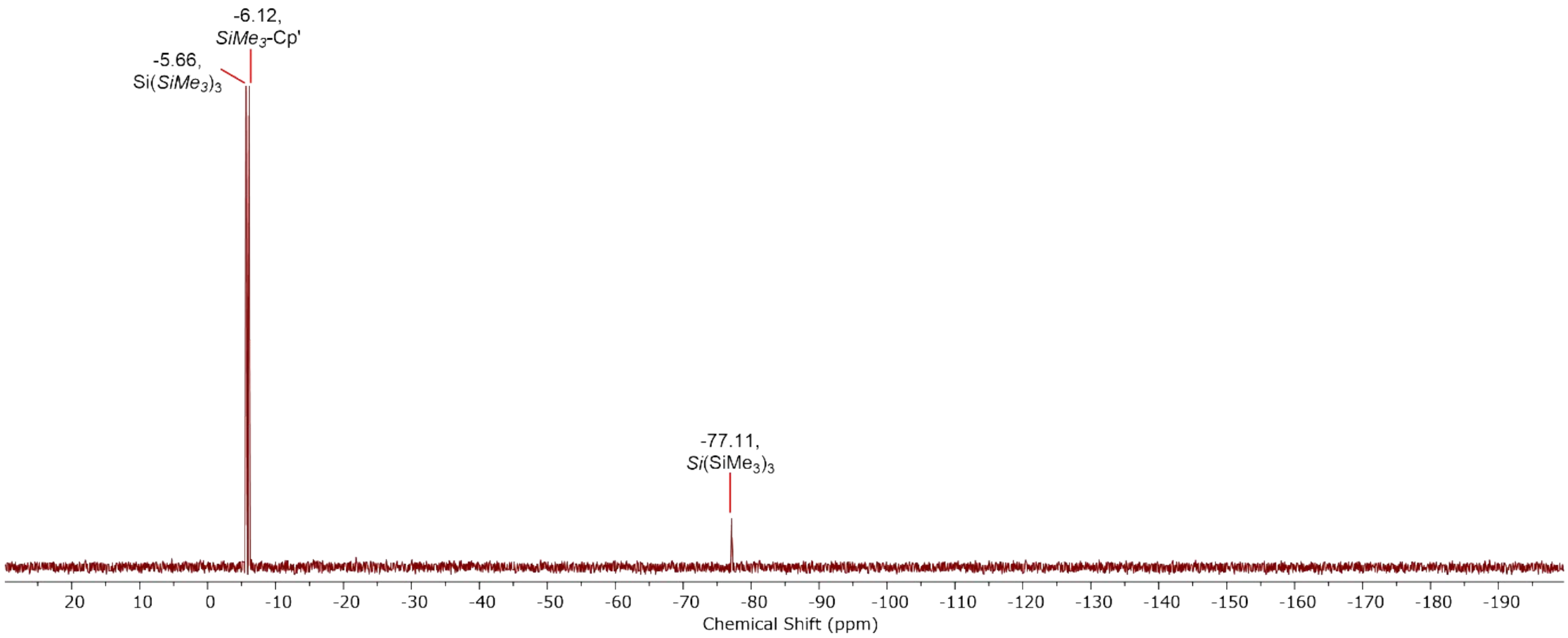

Figure S20. $^{29}Si\{^{1}H\}$ NMR spectrum of **2** in d_6-benzene.

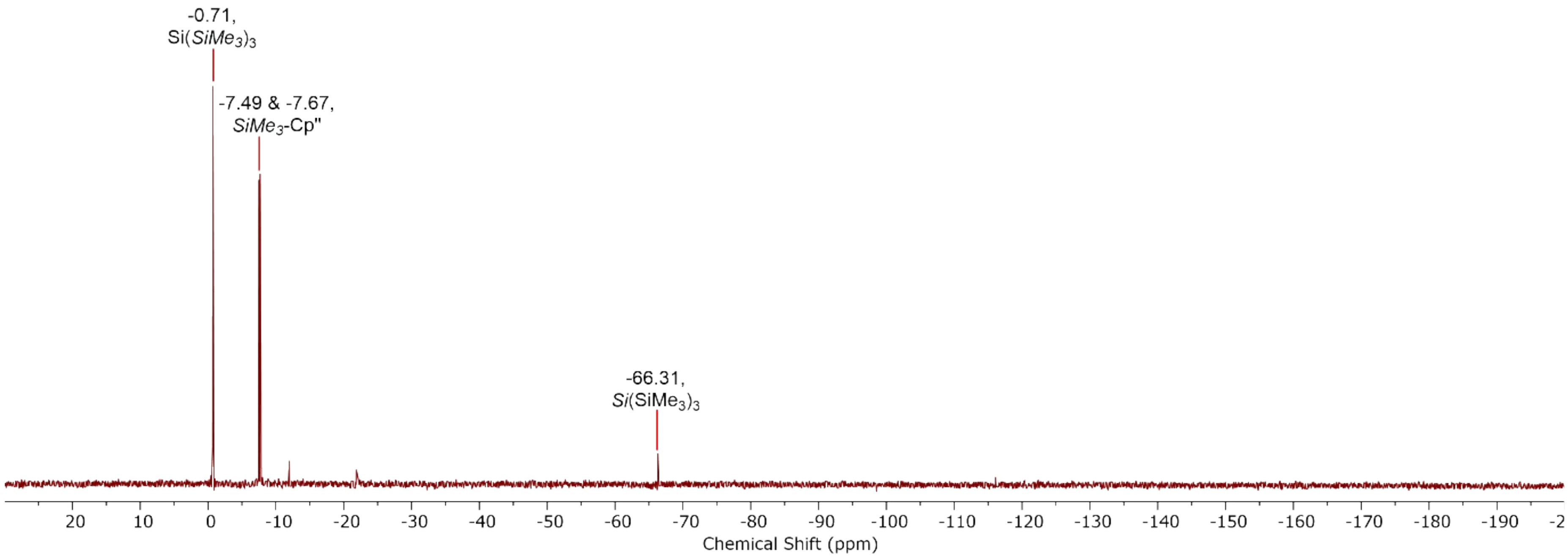

Figure S21. $^{29}Si\{^1H\}$ NMR spectrum of **4** in d_6-benzene.

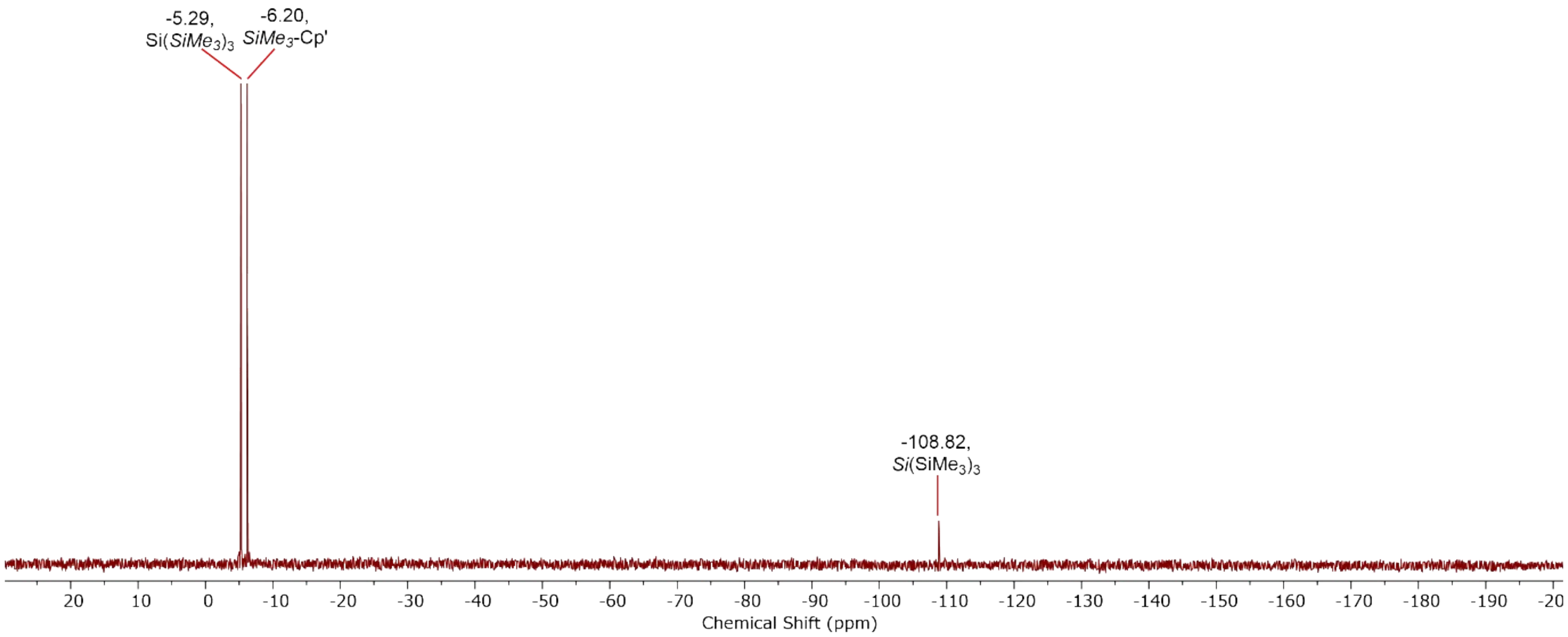

Figure S22. $^{29}Si\{^1H\}$ NMR spectrum of **5** in d_6-benzene.

1.4. 2D NMR spectra of 1, 2, 4 and 5

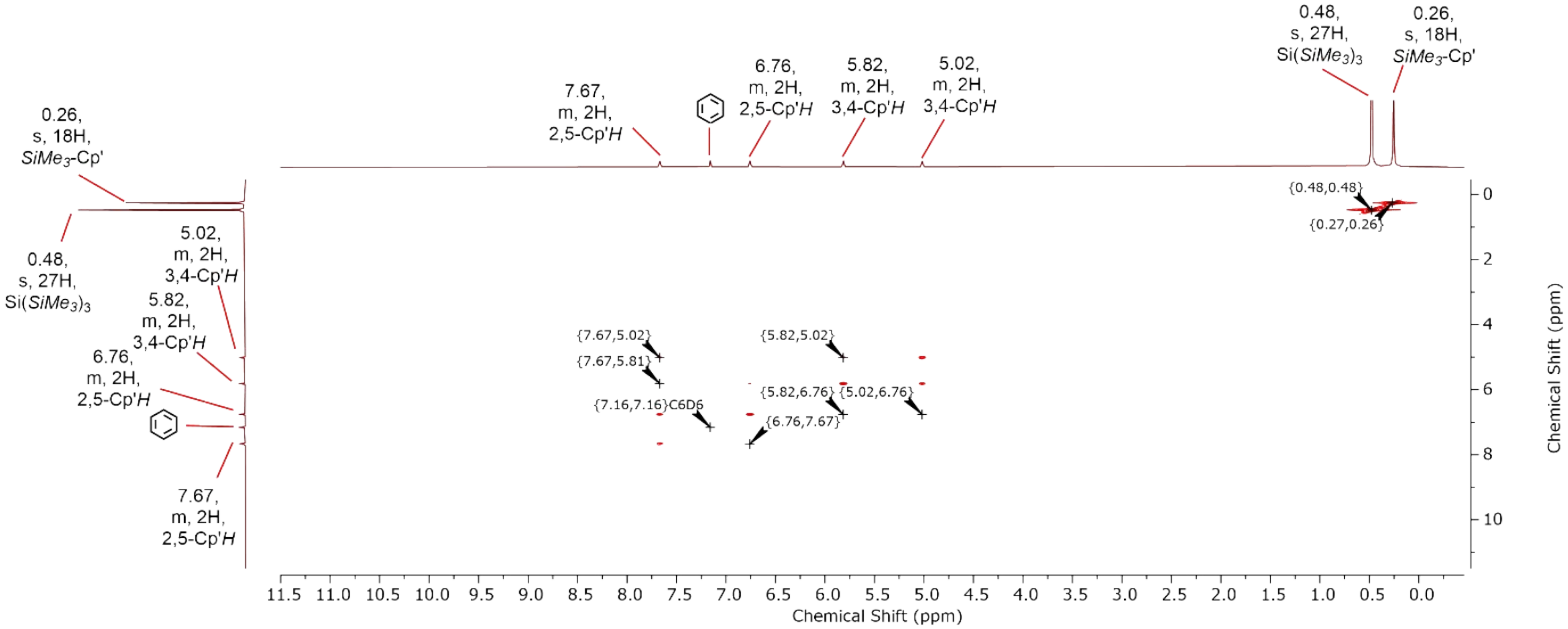

Figure S23. COSY NMR spectrum of **1** in d_6-benzene.

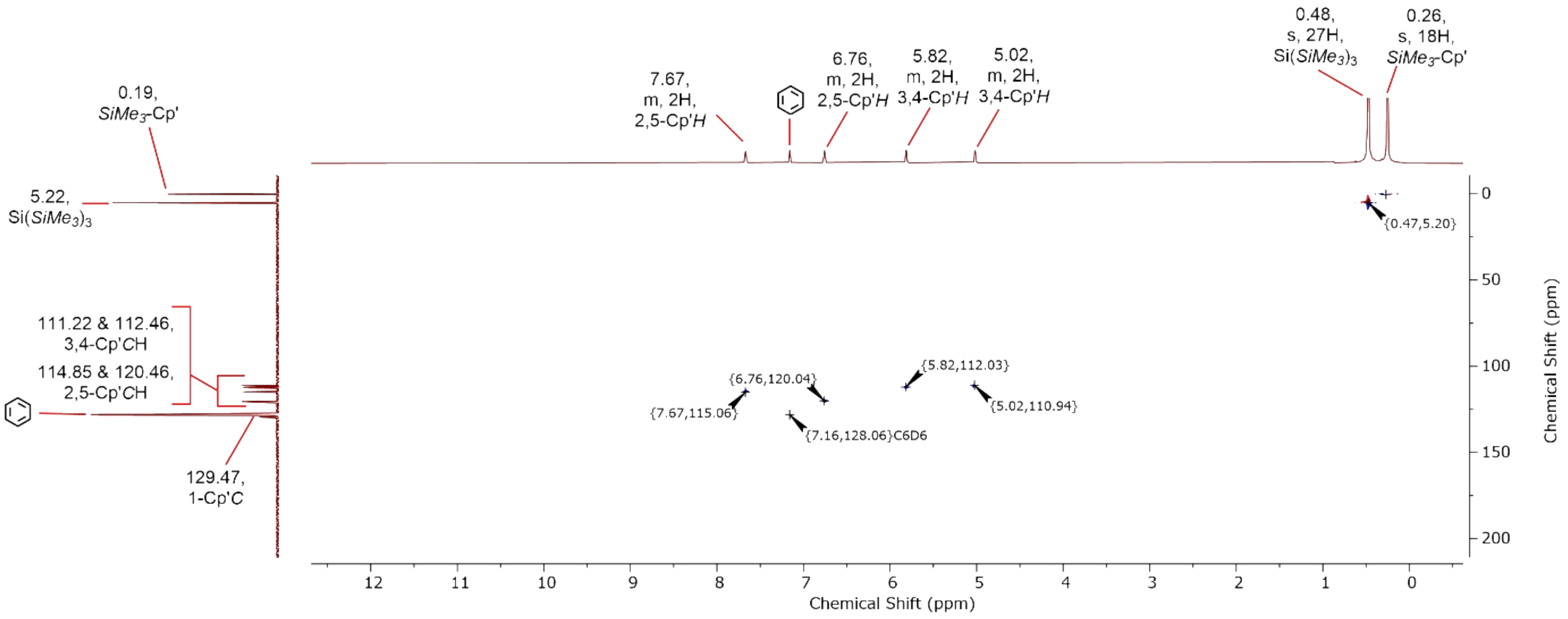

Figure S24. ^{1}H–^{13}C HSQC NMR spectrum of **1** in d_6-benzene.

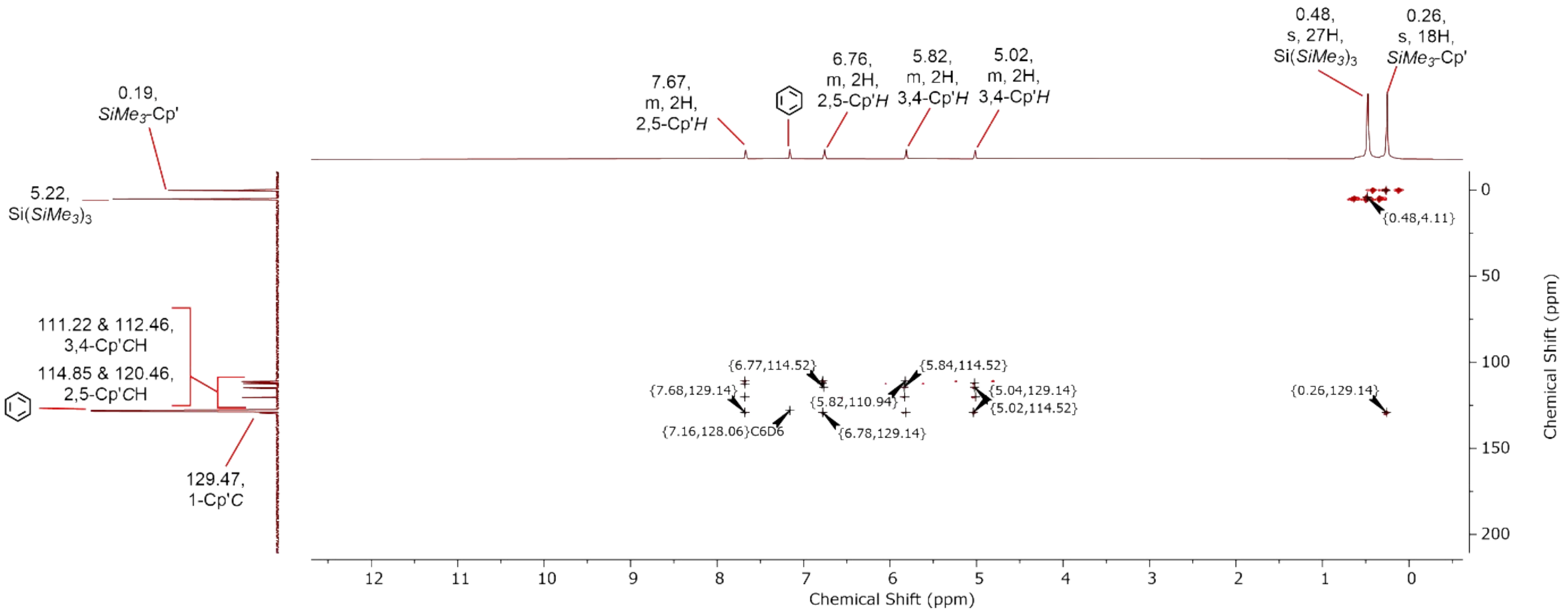

Figure S25. ^{1}H–^{13}C HMBC NMR spectrum of **1** in d_6-benzene.

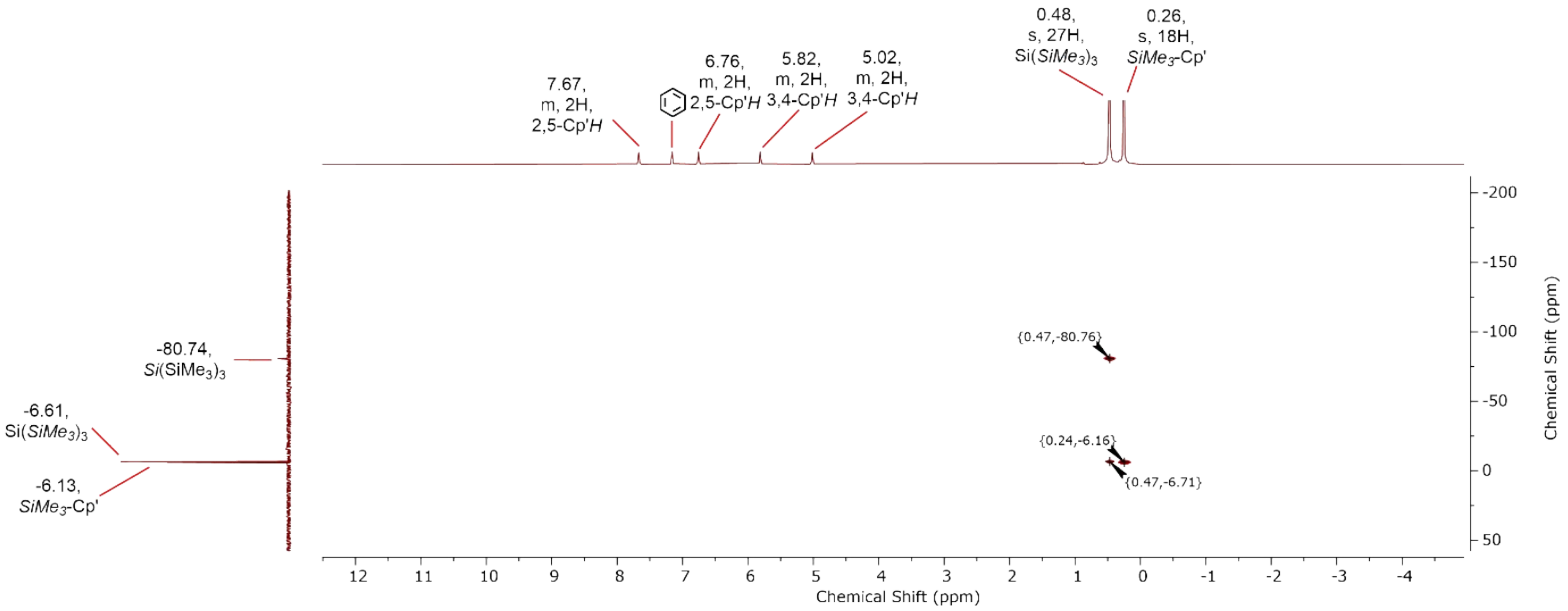

Figure S26. ^{1}H–^{29}Si HMBC NMR spectrum of **1** in d_6-benzene.

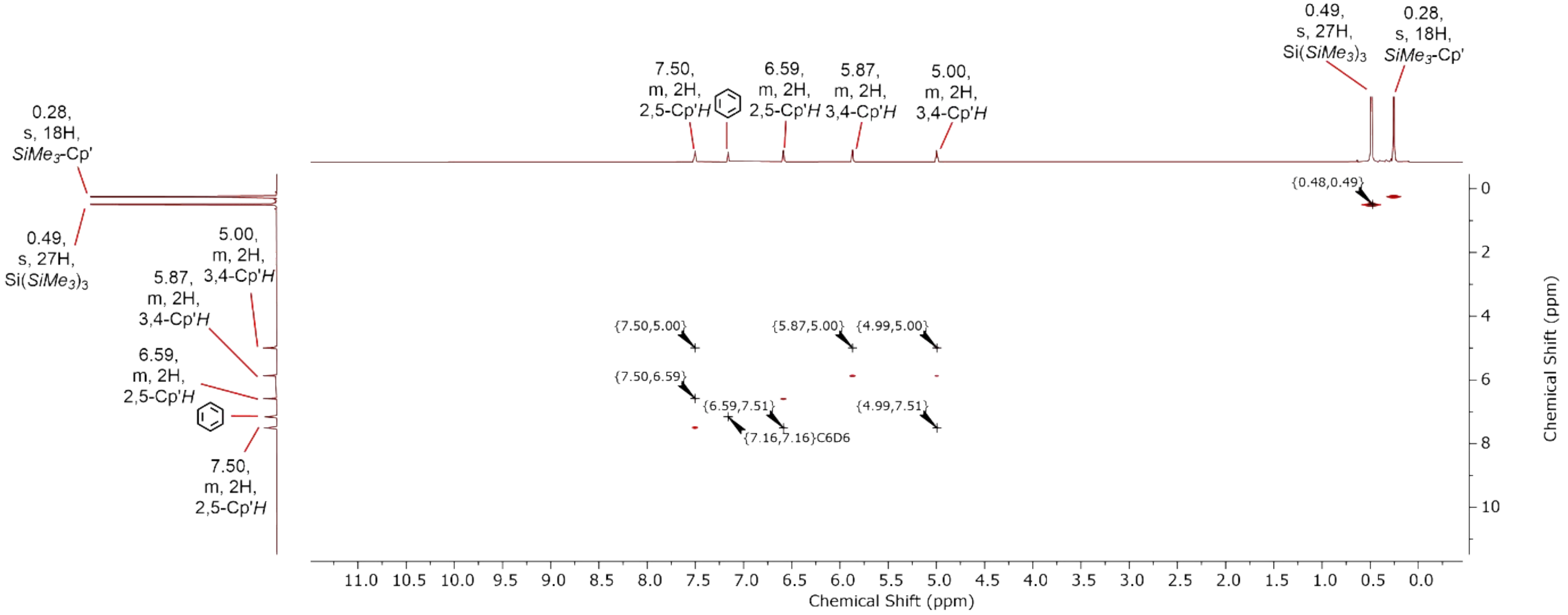

Figure S27. COSY NMR spectrum of **2** in d_6-benzene.

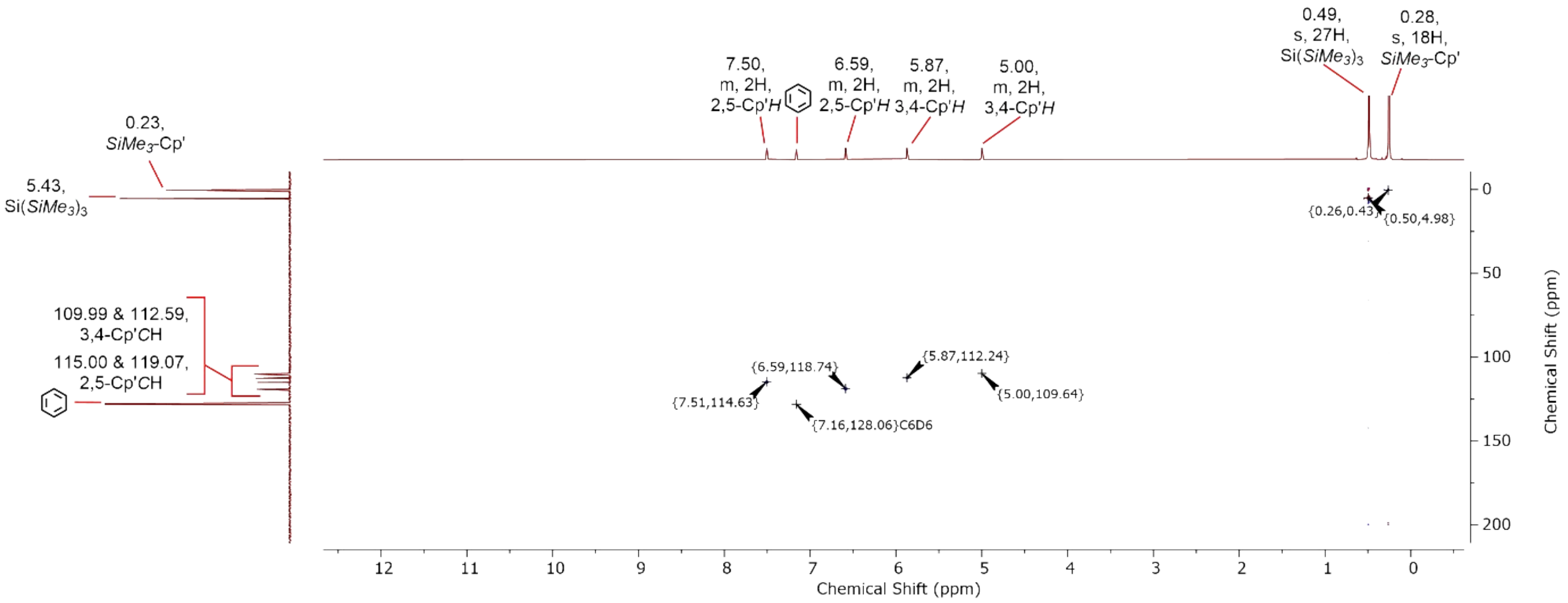

Figure S28. ^1H–^{13}C HSQC NMR spectrum of **2** in d_6-benzene.

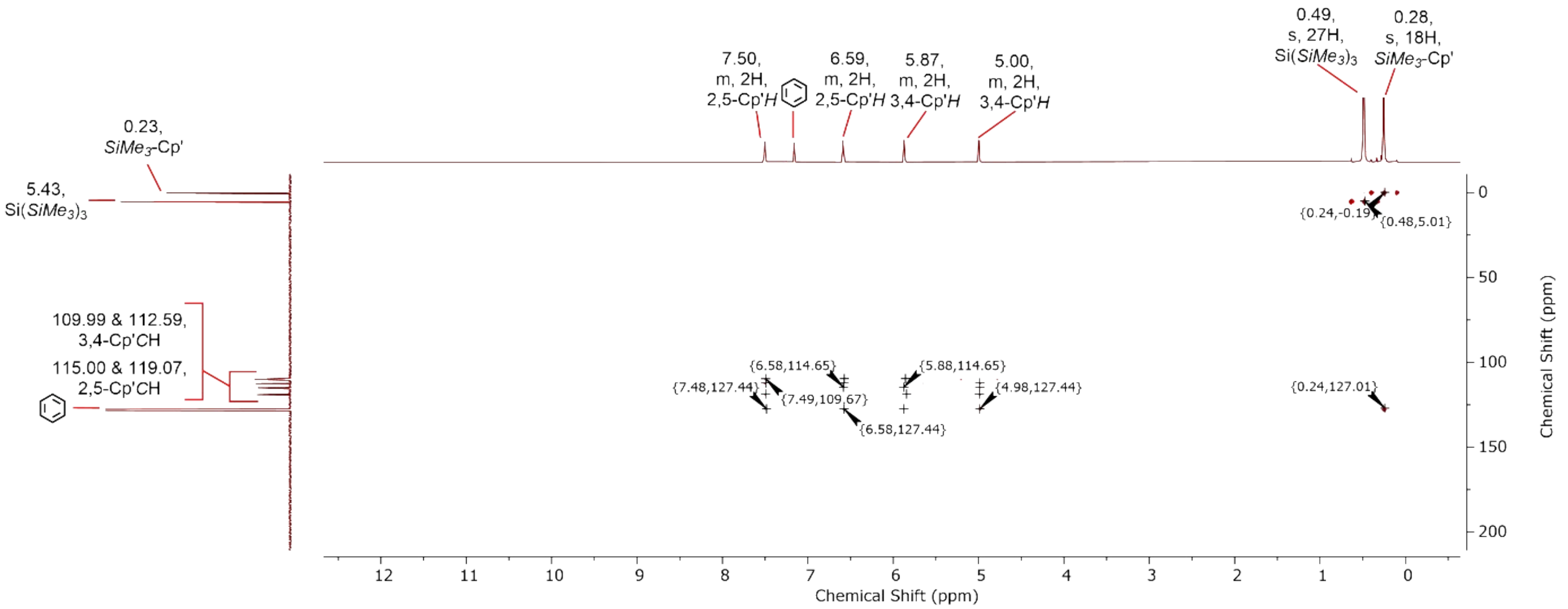

Figure S29. ^{1}H–^{13}C HMBC NMR spectrum of **2** in d_6-benzene.

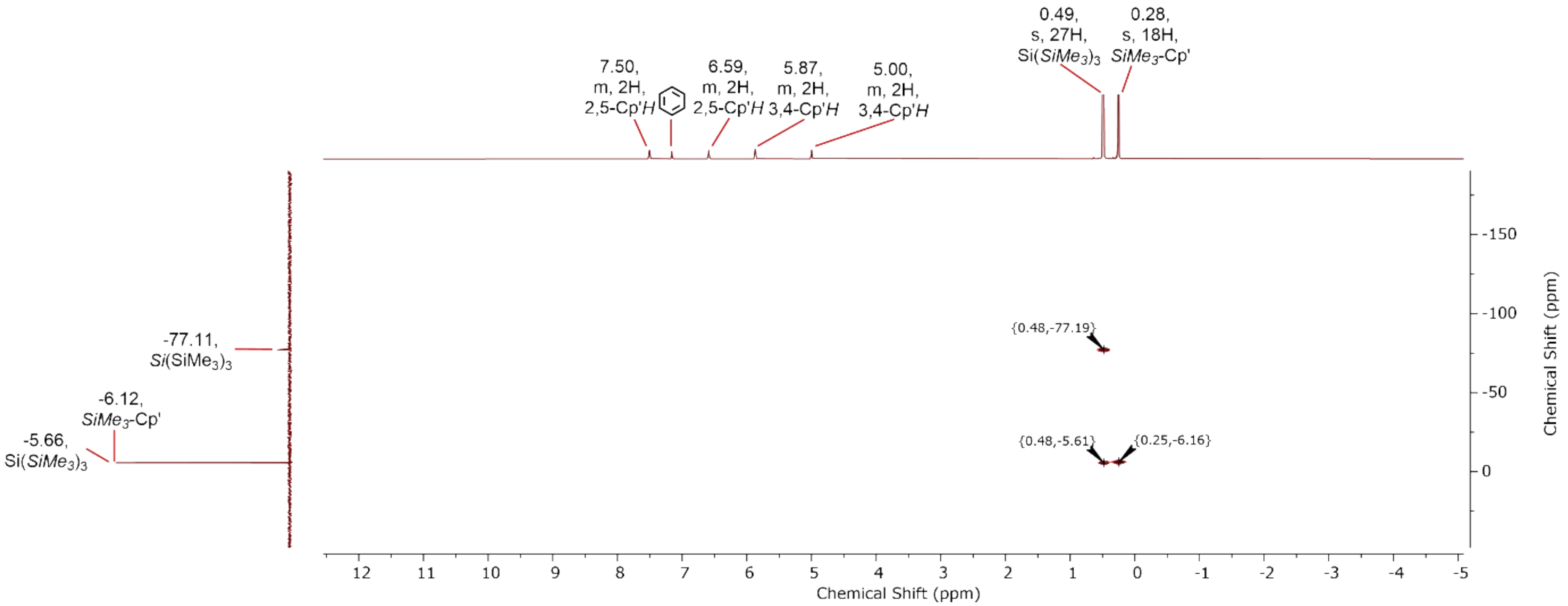

Figure S30. ^{1}H–^{29}Si HMBC NMR spectrum of **2** in d_6-benzene.

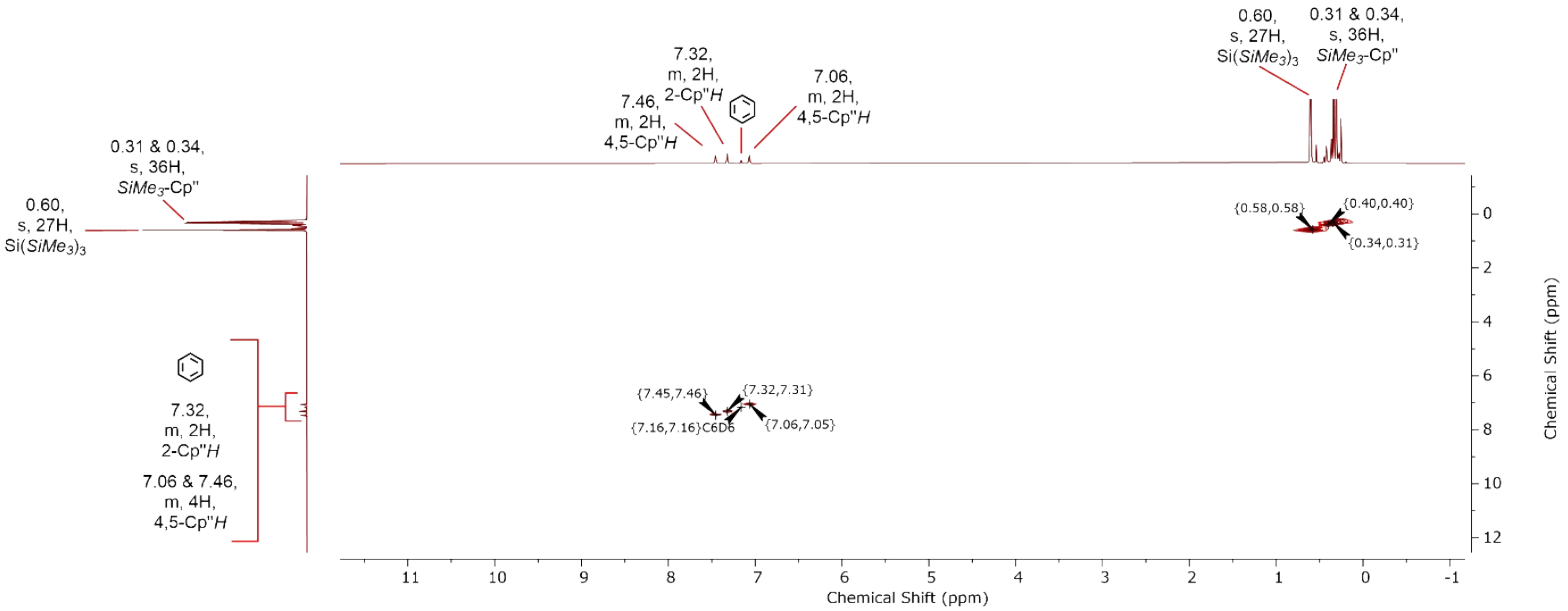

Figure S31. COSY NMR spectrum of **4** in d_6-benzene.

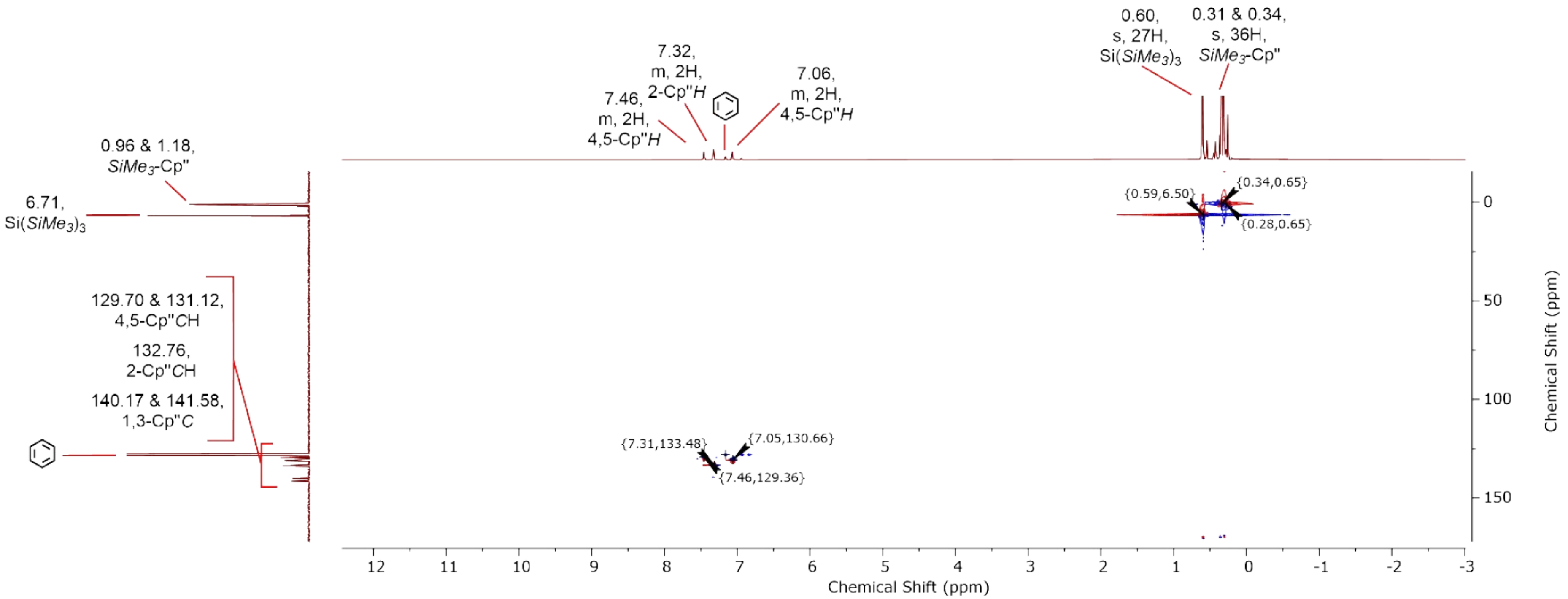

Figure S32. ^{1}H–^{13}C HSQC NMR spectrum of **4** in d_6-benzene.

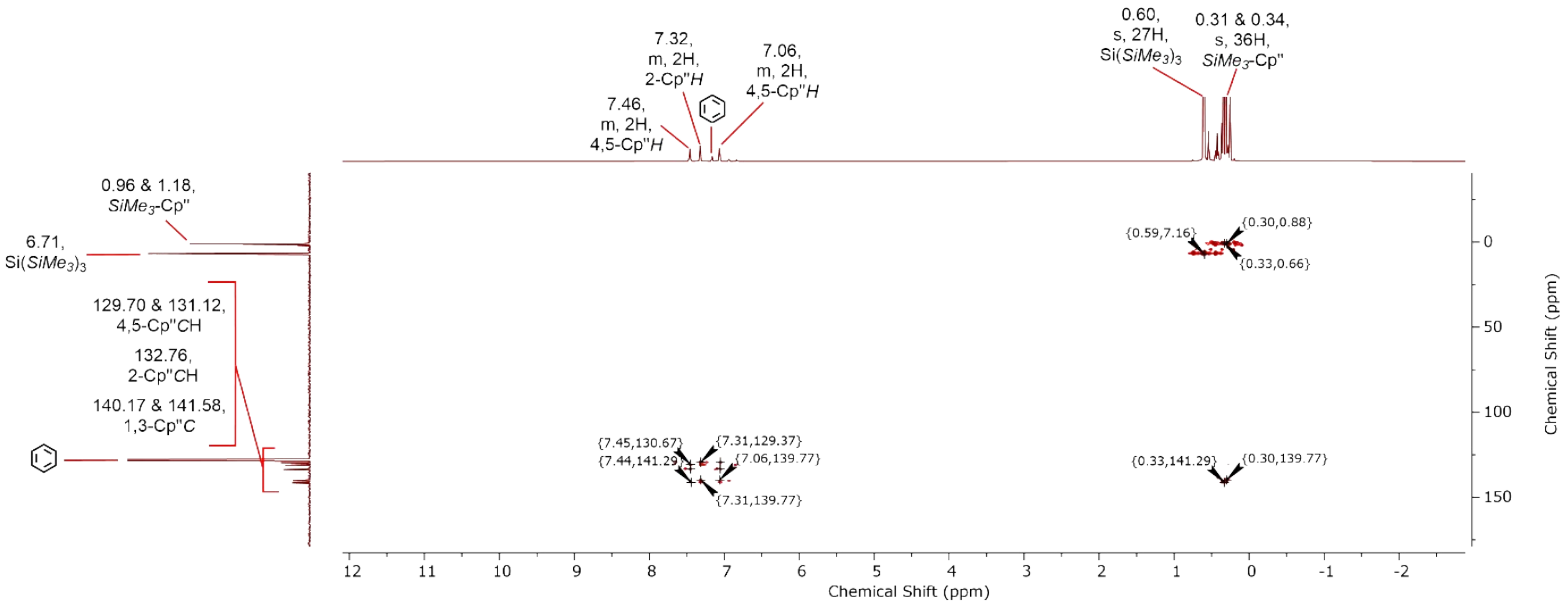

Figure S33. ^{1}H–^{13}C HMBC NMR spectrum of **4** in d_6-benzene.

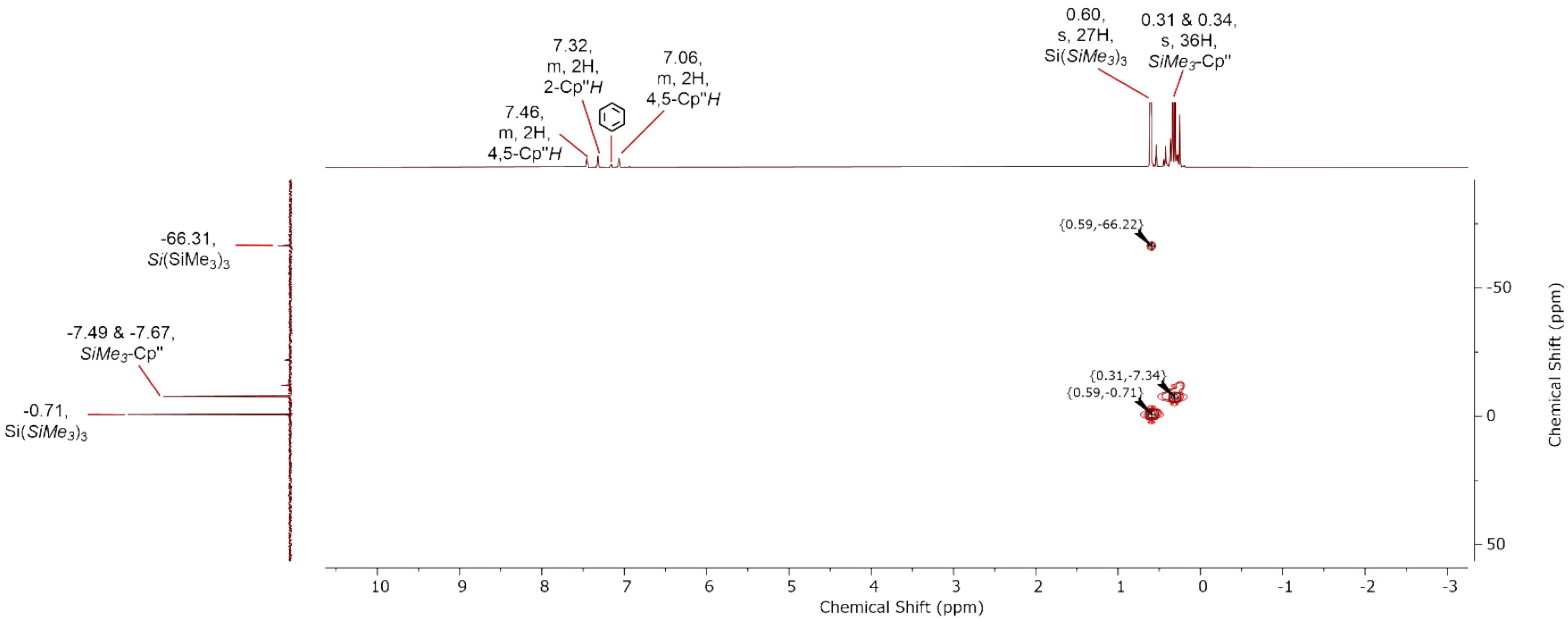

Figure S34. ^{1}H–^{29}Si HMBC NMR spectrum of **4** in d_6-benzene.

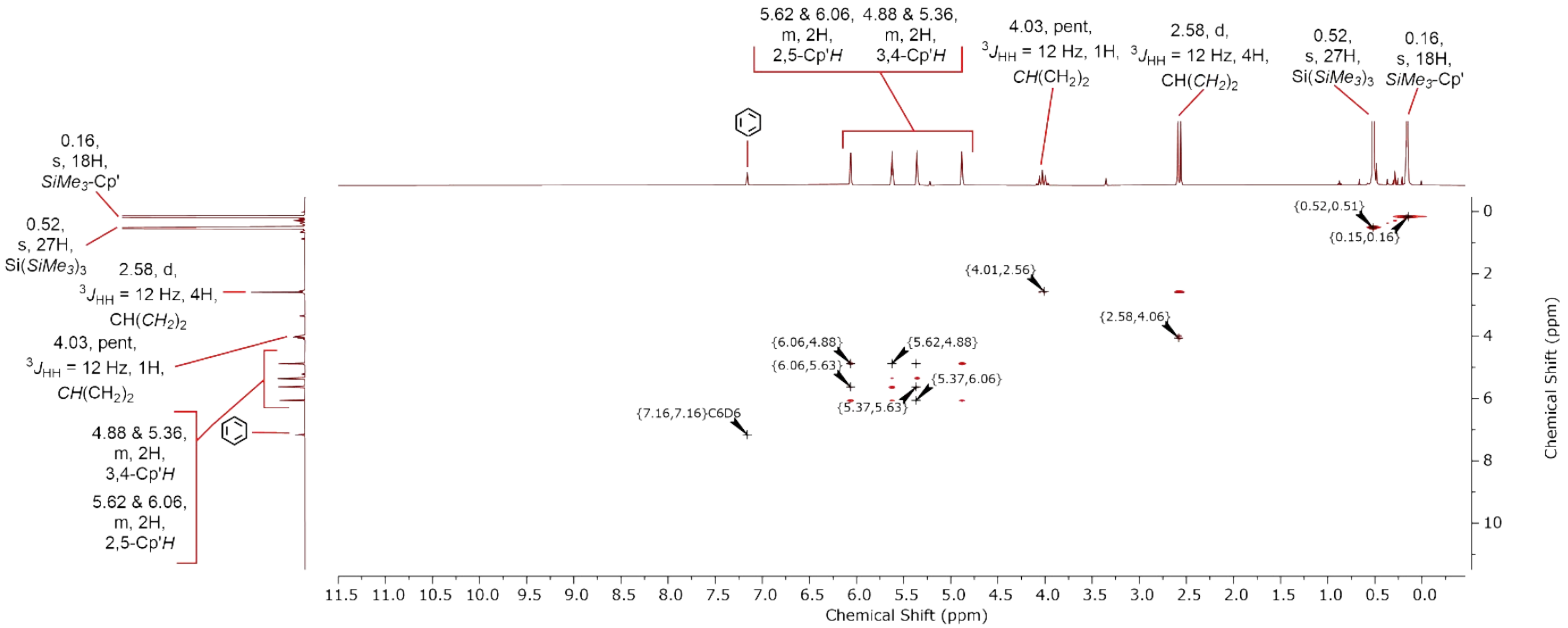

Figure S35. COSY NMR spectrum of **5** in d_6-benzene.

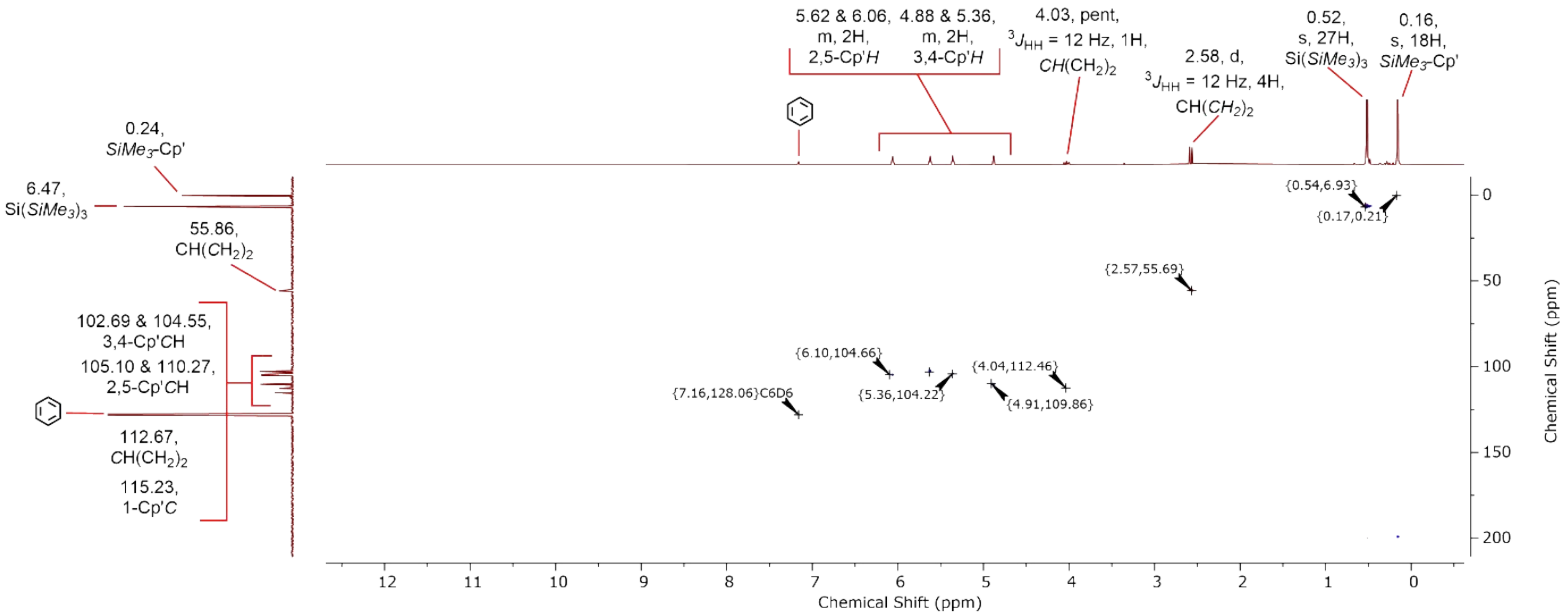

Figure S36. ^{1}H–^{13}C HSQC NMR spectrum of **5** in d_6-benzene.

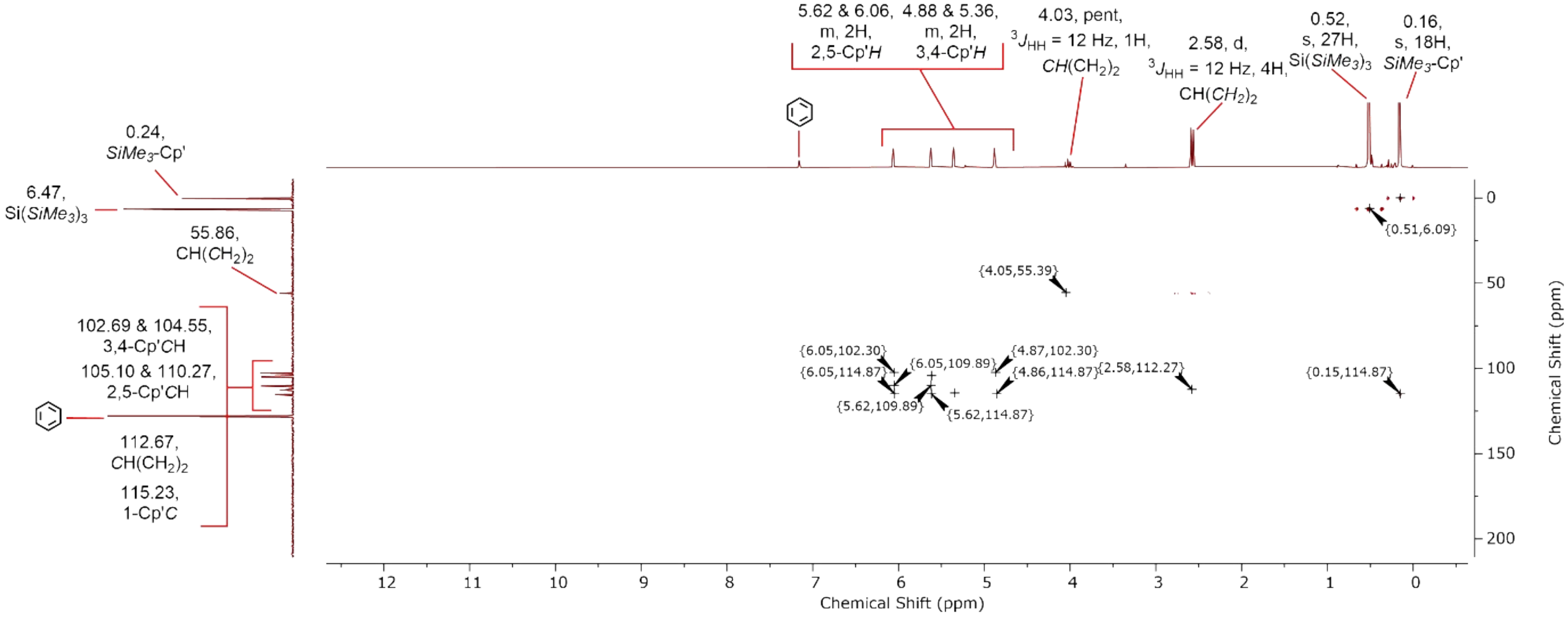

Figure S37. ^{1}H–^{13}C HMBC NMR spectrum of **5** in d_6-benzene.

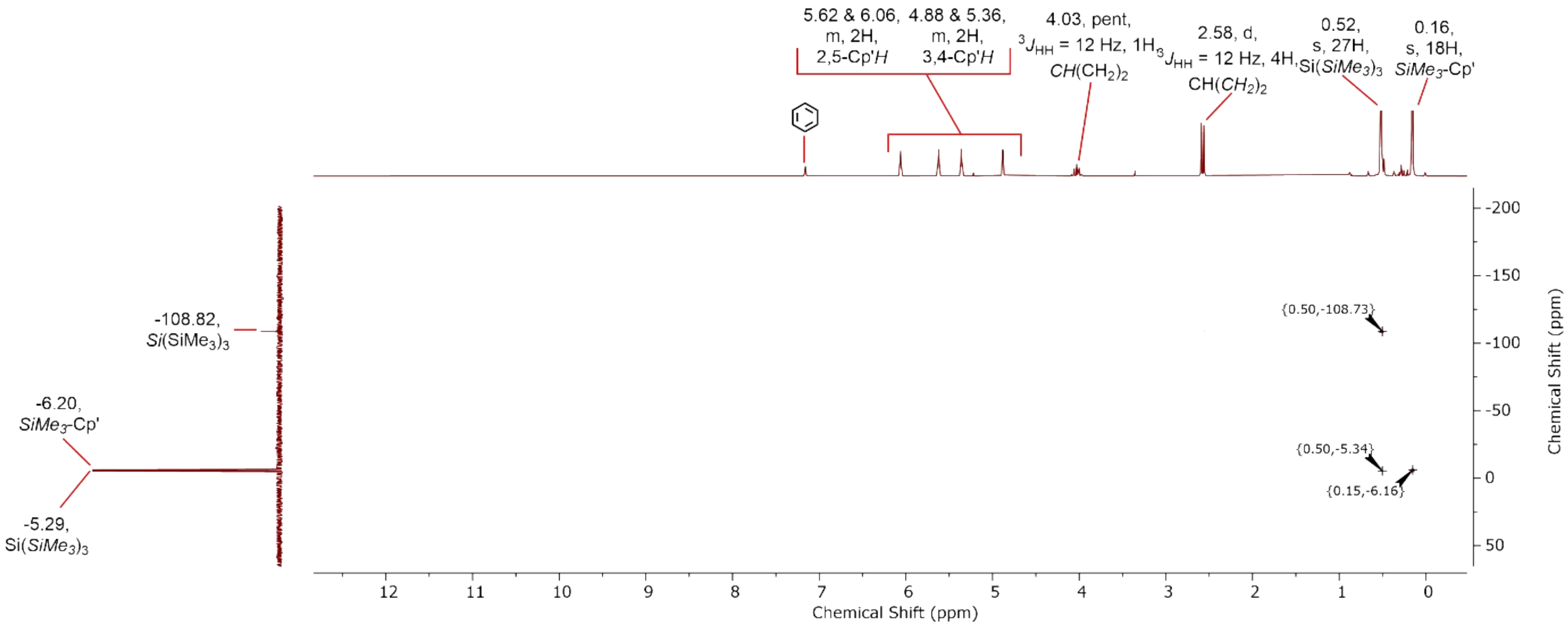

Figure S38. ^{1}H–^{29}Si HMBC NMR spectrum of **5** in d_6-benzene.

2. ATR-IR spectroscopy

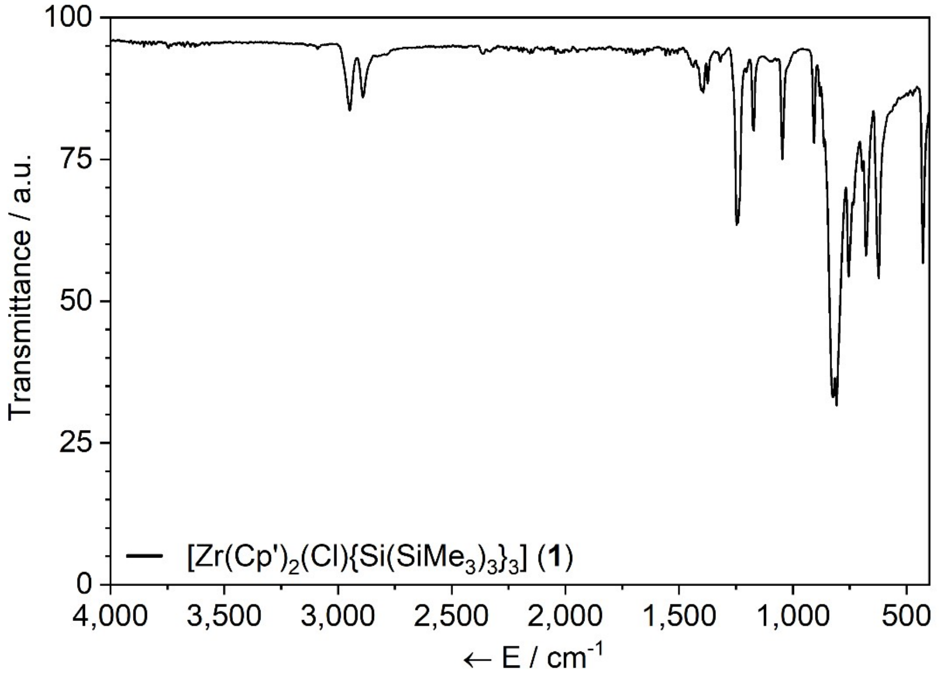

Figure S39. ATR-IR spectrum of **1** between 398-4000 cm^{-1}.

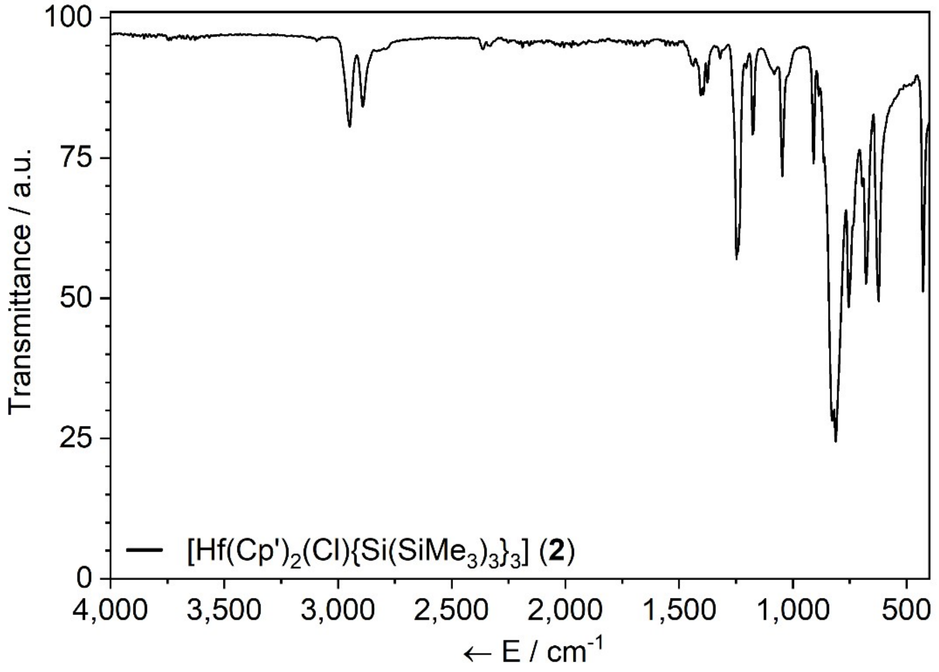

Figure S40. ATR-IR spectrum of **2** between 398-4000 cm^{-1}.

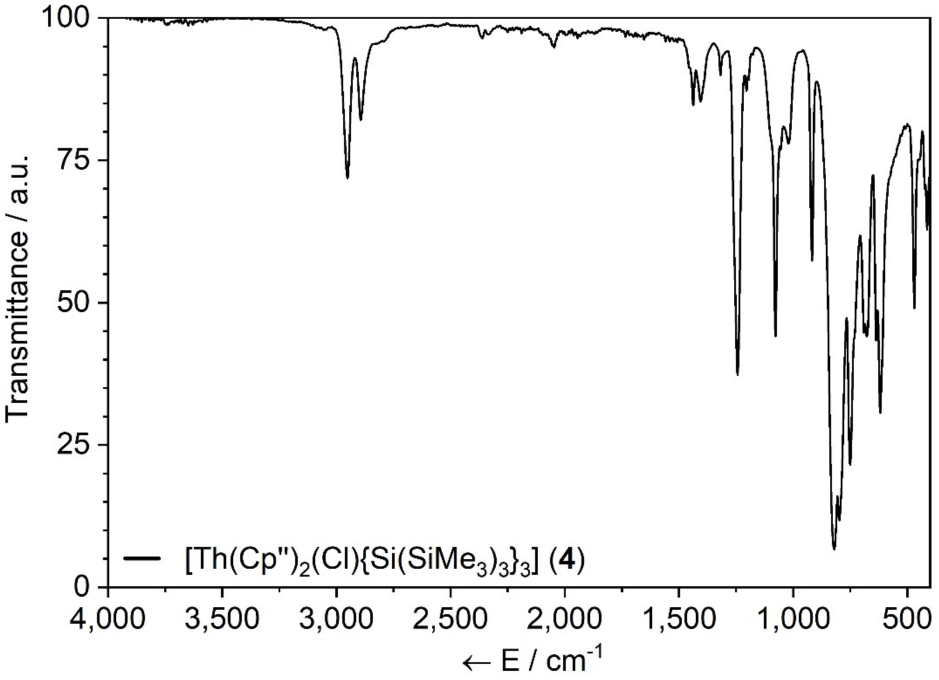

Figure S41. ATR-IR spectrum of **4** between 398-4000 cm^{-1}.

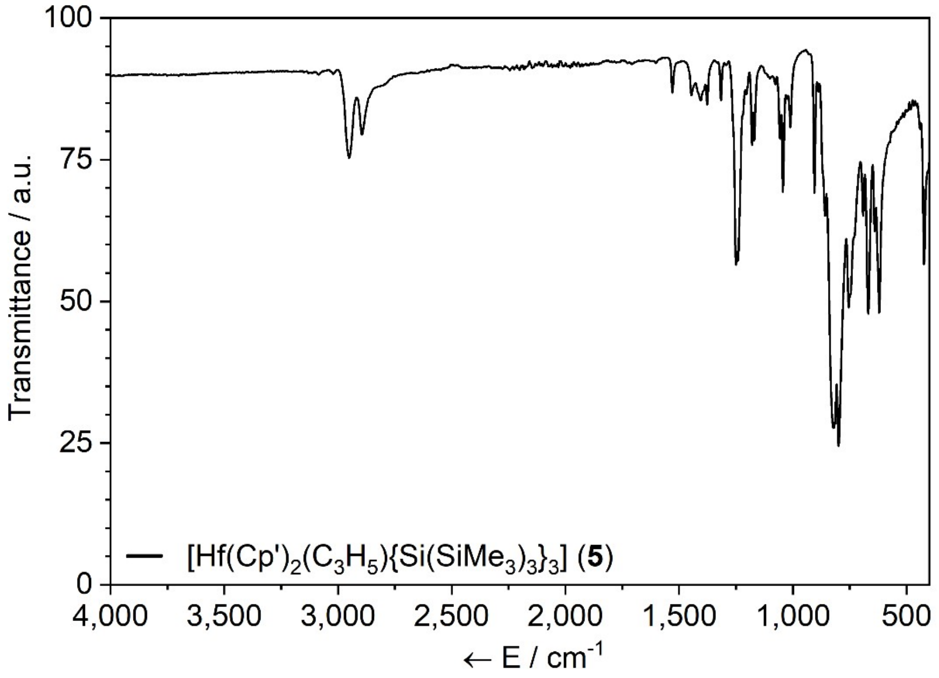

Figure S42. ATR-IR spectrum of **5** between 398-4000 cm^{-1}.

3. Crystallographic data

Table S1. Crystallographic data for [Hf(Cp′)$_2$(Cl)$_2$] and **2**.

	[Hf(Cp′)$_2$(Cl)$_2$]	**2**
Formula	$C_{16}H_{26}Cl_2HfSi_2$	$C_{25}H_{53}ClHfSi_6$
Fw	523.94	736.15
crystal size, mm	0.889 × 0.238 × 0.168	0.840 × 0.148 × 0.139
crystal system	Triclinic	Monoclinic
space group	*P*–1	P*2*$_1$*/c*
a, Å	6.7742(7)	12.6504(3)
b, Å	12.8385(8)	15.5103(3)
c, Å	12.8570(8)	19.0960(5)
α, °	68.257(6)	90
β, °	75.092(8)	106.407(3)
γ, °	81.994(2)	90
V, Å^3	1002.46(14)	3594.28(15)
Z	2	4
$\rho_{calcd,\ g\ cm^{-3}}$	1.736	1.360
μ, mm^{-1}	5.581	3.189
no. of reflections made	3670	23140
no. of unique reflns R_{int}	3670, 0.0512	8467, 0.0375
no. of reflns with $F^2 > 2\sigma(F^2)$	3468	7137
transmn coeff range	0.079-1.000	0.752-1.000
R, R_w[a] ($F^2 > 2\sigma(F^2)$)	0.0695, 0.2064	0.0310, 0.0551
R, R_w[a] (all data)	0.0723, 0.2089	0.0409, 0.0596
S[a]	1.147	1.039
no. of parameters, restraints	197, 114	313, 0
max., min. diff map, e Å^{-3}	7.083, -2.500	0.923, -0.652

[a] Conventional $R = \Sigma||F_o| - |F_c||/\Sigma|F_o|$; $R_w = [\Sigma w(F_o^2 - F_c^2)^2/\Sigma w(F_o^2)^2]^{1/2}$; $S = [\Sigma w(F_o^2 - F_c^2)^2/\text{no. data} - \text{no. params})]^{1/2}$ for all data.

Table S2. Crystallographic data for **3·0.5C$_5$H$_{12}$** and **4**.

	**3·0.5C$_5$H$_{12}$**	**4**
Formula	$C_{28}H_{61}ClHfSi_7 \cdot 0.5C_5H_{12}$	$C_{31}H_{69}ClSi_8Th$
Fw	844.41	934.07
crystal size, mm	0.350 × 0.171 × 0.164	0.319 × 0.273 × 0.173
crystal system	Monoclinic	Monoclinic
space group	P$2_1/n$	P$2_1/c$
a, Å	13.53762(7)	19.9729(11)
b, Å	16.04434(7)	35.7285(5)
c, Å	19.91805(9)	11.2395(6)
α, °	90	90
β, °	100.0313(4)	144.858(12)
γ, °	90	90
V, Å^3	4260.11(3)	4616.6(9)
Z	4	4
$\rho_{calcd,\ g\ cm^{-3}}$	1.317	1.344
μ, mm^{-1}	7.119	13.069
no. of reflections made	54010	43727
no. of unique reflns, R_{int}	8678, 0.0421	9459, 0.0442
no. of reflns with $F^2 > 2\sigma(F^2)$	8669	9392
transmn coeff range	0.111-0.788	0.357-1.000
R, R_w[a] ($F^2 > 2\sigma(F^2)$)	0.0329, 0.0876	0.0432, 0.1177
R, R_w[a] (all data)	0.0329, 0.0876	0.0.0434, 0.1180
S[a]	1.161	1.097
no. of parameters, restraints	352, 0	391, 0
max., min. diff map, e Å^{-3}	1.658, -1.964	5.455, -3.443

[a] Conventional $R = \Sigma||F_o| - |F_c||/\Sigma|F_o|$; $R_w = [\Sigma w(F_o^2 - F_c^2)^2/\Sigma w(F_o^2)^2]^{1/2}$; $S = [\Sigma w(F_o^2 - F_c^2)^2/\text{no. data} - \text{no. params})]^{1/2}$ for all data.

Table S3. Crystallographic data for **5** and **6**.

	**5**	**6**
Formula	$C_{28}H_{58}HfSi_6$	$C_{30}H_{40}HfSi_2$
Fw	741.77	635.29
crystal size, mm	0.893 × 0.559 × 0.416	0.239 × 0.190 × 0.147
crystal system	Monoclinic	Triclinic
space group	$P2_1/c$	P–1
a, Å	9.3391(3)	12.1816(6)
b, Å	32.1430(10)	13.2144(6)
c, Å	11.9936(5)	20.7629(8)
α, °	90	86.723(3)
β, °	96.875(3)	74.850(4)
γ, °	90	63.240(5)
V, $Å^3$	3574.5(2)	2873.5(3)
Z	4	4
$\rho_{calcd,\ g\ cm^{-3}}$	1.378	1.468
μ, mm^{-1}	3.135	3.729
no. of reflections made	15712	24285
no. of unique reflns, R_{int}	6774, 0.0570	13234, 0.0512
no. of reflns with $F^2 > 2\sigma(F^2)$	8127	9151
transmn coeff range	0.210-1.000	0.538-1.000
R, R_w[a] ($F^2 > 2\sigma(F^2)$)	0.0519, 0.1093	0.0530, 0.0821
R, R_w[a] (all data)	0.0638, 0.1156	0.0882, 0.0979
S[a]	1.066	1.027
no. of parameters, restraints	331, 0	607, 0
max., min. diff map, e $Å^{-3}$	3.682, -2.306	2.419, -1.159

[a] Conventional $R = \Sigma||F_o| - |F_c||/\Sigma|F_o|$; $R_w = [\Sigma w(F_o^2 - F_c^2)^2/\Sigma w(F_o^2)^2]^{1/2}$; $S = [\Sigma w(F_o^2 - F_c^2)^2/\text{no. data} - \text{no. params})]^{1/2}$ for all data.

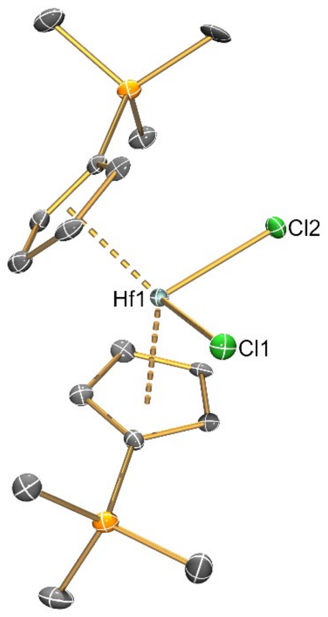

Figure S43. Single crystal XRD structure of [Hf(Cp′)$_2$(Cl)$_2$] with selective atom labelling. Displacement ellipsoids set at 30 % probability level and hydrogen atoms omitted for clarity.

4. NBO representations of selected frontier orbitals of 1-6

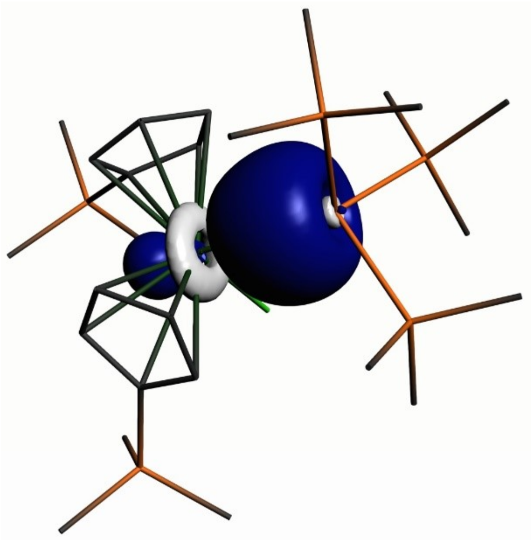

Figure S44. NBO depiction of the Zr–Si σ-bond in **1** (HOMO). Hydrogen atoms are omitted for clarity.

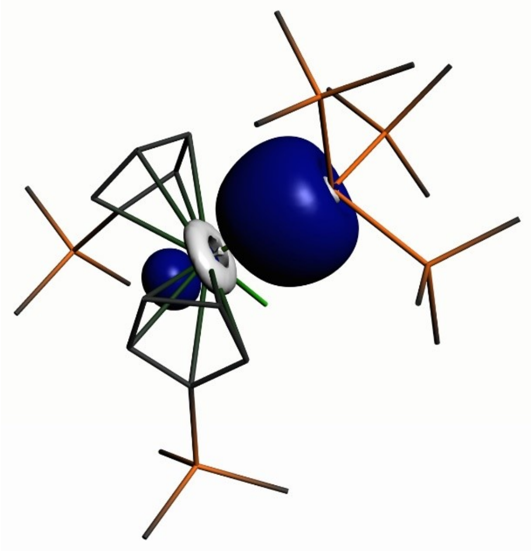

Figure S45. NBO depiction of the Hf–Si σ-bond in **2** (HOMO). Hydrogen atoms are omitted for clarity.

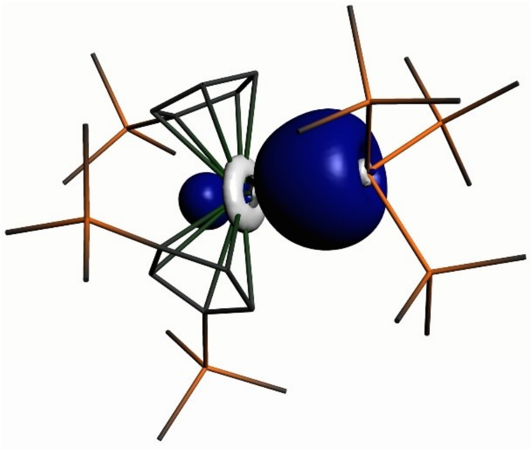

Figure S46. NBO depiction of the Hf–Si σ-bond in **3** (HOMO). Hydrogen atoms are omitted for clarity.

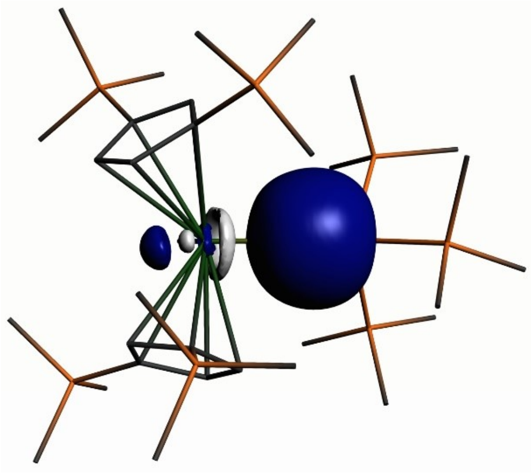

Figure S47. NBO depiction of the Th–Si σ-bond in **4** (HOMO). Hydrogen atoms are omitted for clarity.

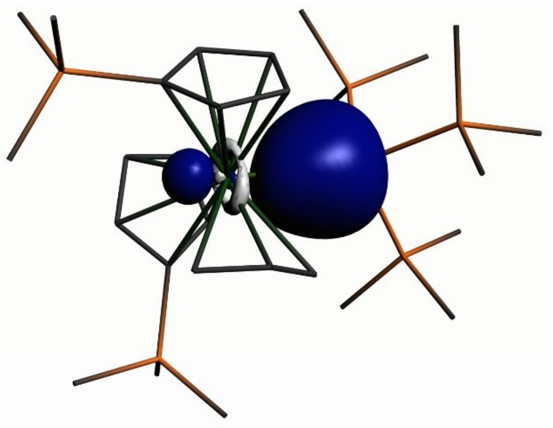

Figure S48. NBO depiction of the Hf–Si σ-bond in **5** (HOMO). Hydrogen atoms are omitted for clarity.

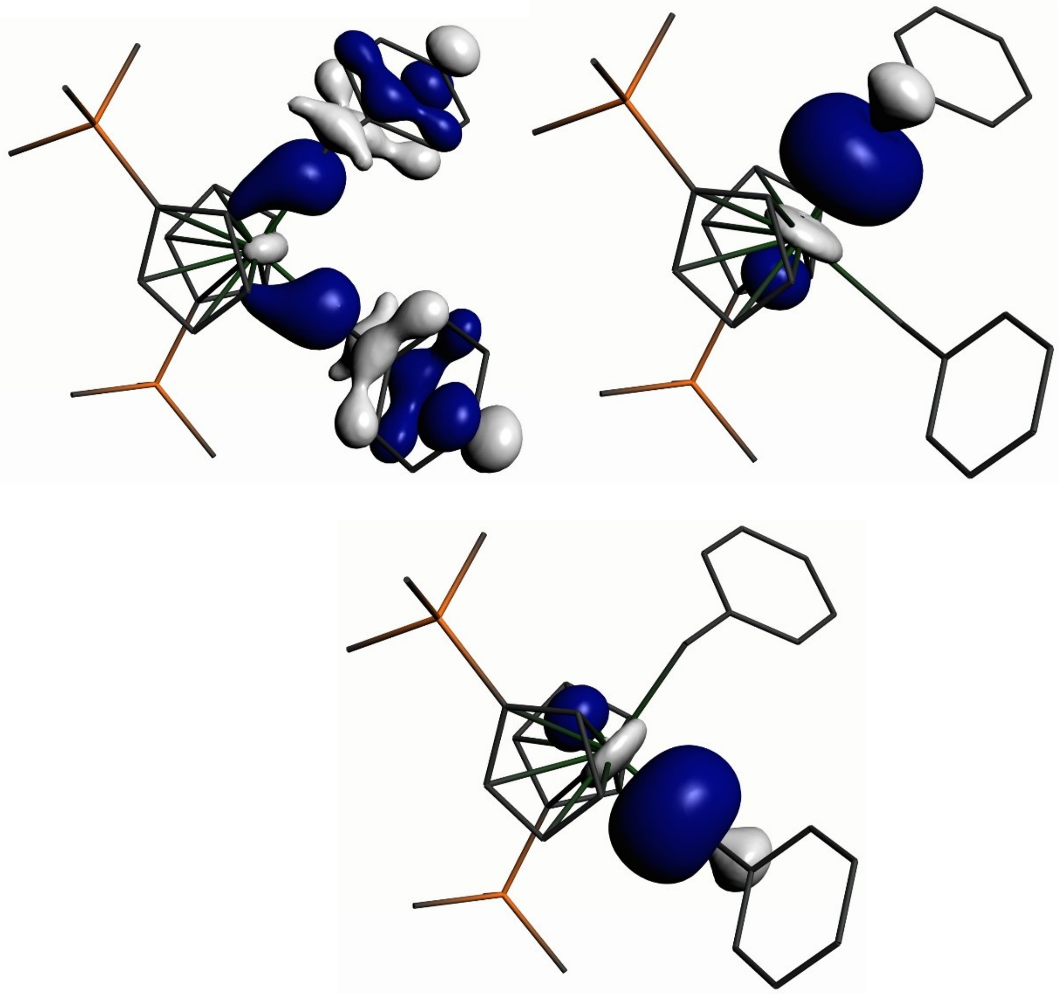

Figure S49. NBO depiction of the Hf–C σ-bonds in **6** (HOMO, top left; HOMO – 1 top right). Bottom: Kohn-Sham representation of the Hf–C σ-bonds (HOMO−1 159, −4.939 eV). Hydrogen atoms are omitted for clarity.

5. Optimised geometry coordinates for 1-6

Table S4. Final coordinates and energy from a single point energy calculation on geometry optimised **1**.

1.C	3.101521	-0.669105	-4.600798
2.C	0.284376	0.457255	-4.422232
3.C	0.739674	-2.532305	-4.031181
4.C	3.087688	2.746287	-2.107542
5.C	4.172457	-3.115331	-1.711307
6.C	5.162742	0.585539	-1.586623
7.C	-2.037384	-1.388336	-1.470463
8.C	-0.453745	2.179270	-1.280024
9.C	-1.843414	1.915254	-1.174279
10.C	-1.062748	-2.245704	-0.880263
11.C	-2.961026	-1.021091	-0.458286
12.C	1.818285	-3.974734	-0.024328
13.C	0.008640	2.562723	0.010406
14.C	-2.224218	2.113891	0.185618
15.C	-1.386452	-2.381184	0.491654
16.C	3.677506	1.784063	0.758497
17.C	-2.567510	-1.618128	0.782935
18.C	-1.078525	2.547791	0.934260
19.C	3.822846	-2.045289	1.159363
20.C	-4.638995	-3.337505	2.171739
21.C	-2.036206	4.916714	2.549288
22.C	-4.773043	-0.277526	2.486112
23.C	0.763771	3.692276	3.100747
24.C	-2.518146	-1.920494	3.882388
25.C	-1.803369	2.227920	4.000879
26.H	2.920549	-0.864451	-5.670793
27.H	0.142263	0.217562	-5.488532
28.H	0.754271	-2.695715	-5.121341
29.H	3.535638	0.338063	-4.514025
30.H	0.685251	1.480754	-4.369209
31.H	3.854087	-1.389467	-4.247989
32.H	-0.706513	0.461463	-3.945581
33.H	-0.303380	-2.633353	-3.698248
34.H	1.318743	-3.345459	-3.568224
35.H	2.978001	2.487583	-3.171487
36.H	5.177158	0.253749	-2.634653
37.H	3.686119	-3.477230	-2.630166
38.H	-2.073850	-1.089248	-2.513977
39.H	3.933324	3.448575	-2.025815
40.H	4.909284	-2.351974	-1.999232
41.H	0.139482	2.132572	-2.185427
42.H	-2.501617	1.623548	-1.987750
43.H	2.181329	3.285921	-1.799845
44.H	5.889879	1.407896	-1.481813

45.H	4.719921	-3.964775	-1.269355
46.H	-0.247283	-2.734199	-1.396911
47.H	5.516759	-0.248906	-0.964293
48.H	1.231669	-4.356824	-0.872108
49.H	-3.832775	-0.388745	-0.603329
50.H	2.487722	-4.787896	0.300878
51.H	1.031881	2.828171	0.251058
52.H	-3.234449	2.018305	0.573955
53.H	4.498776	2.518537	0.805138
54.H	1.129736	-3.767302	0.805255
55.H	4.650713	-1.340888	0.994644
56.H	-5.279076	-3.297670	1.277624
57.H	2.782132	2.251624	1.189397
58.H	-0.825434	-2.965604	1.213206
59.H	3.942325	0.940693	1.413105
60.H	4.247525	-2.965808	1.592880
61.H	-5.434213	-0.205592	1.609288
62.H	-1.620309	5.593410	1.787638
63.H	3.142433	-1.601625	1.901506
64.H	-3.986907	-4.219005	2.073892
65.H	-3.080679	4.705477	2.271238
66.H	1.223295	4.381664	2.377020
67.H	-4.219296	0.667967	2.576160
68.H	-5.286784	-3.492428	3.049257
69.H	1.360255	2.768335	3.125007
70.H	-2.046939	5.450930	3.512610
71.H	-5.416084	-0.372344	3.374924
72.H	-1.798509	-2.745822	3.770818
73.H	-2.853365	1.984014	3.782165
74.H	0.830511	4.161609	4.094608
75.H	-1.939523	-1.005212	4.070097
76.H	-1.246157	1.287622	4.111838
77.H	-3.132846	-2.132781	4.771599
78.H	-1.784014	2.755892	4.967361
79.Cl	0.444797	-0.223583	2.312116
80.Zr	-0.710819	0.070395	0.166691
81.Si	1.470943	-0.813352	-3.624365
82.Si	1.726741	-0.490980	-1.258086
83.Si	3.439144	1.198118	-1.040163
84.Si	2.896629	-2.456390	-0.454245
85.Si	-3.607670	-1.761705	2.353270
86.Si	-1.031601	3.315942	2.665224

Energy: –459.16437148 eV

Table S5. Final coordinates and energy from a single point energy calculation on geometry optimised **2**.

1.C	3.094224	-0.675975	-4.593497
2.C	0.287997	0.462530	-4.427253
3.C	0.729871	-2.525652	-4.024032
4.C	3.081560	2.744436	-2.109335
5.C	4.173885	-3.100011	-1.731091
6.C	5.157697	0.584682	-1.597686
7.C	-2.006086	-1.378863	-1.461377
8.C	-0.453207	2.140416	-1.270536
9.C	-1.841338	1.872259	-1.164070
10.C	-1.028984	-2.228174	-0.862482
11.C	-2.933946	-1.011357	-0.454670
12.C	1.835636	-3.987656	-0.050040
13.C	0.009387	2.528945	0.018693
14.C	-2.220480	2.067289	0.197280
15.C	-1.355844	-2.359167	0.508192
16.C	3.695659	1.791662	0.752492
17.C	-2.539608	-1.596544	0.791981
18.C	-1.077207	2.513116	0.942679
19.C	3.834458	-2.057056	1.145502
20.C	-4.646311	-3.303139	2.162776
21.C	-2.039774	4.902322	2.542042
22.C	-4.760485	-0.243978	2.456196
23.C	0.751606	3.687610	3.113068
24.C	-2.541442	-1.900614	3.894416
25.C	-1.820772	2.226953	4.007631
26.H	2.902881	-0.863544	-5.663163
27.H	0.163480	0.220241	-5.495331
28.H	0.730387	-2.678966	-5.115620
29.H	3.541119	0.325435	-4.505815
30.H	0.686356	1.486838	-4.370941
31.H	3.840373	-1.407434	-4.250774
32.H	-0.711249	0.466796	-3.967974
33.H	-0.307960	-2.634769	-3.678448
34.H	1.317961	-3.340932	-3.577211
35.H	2.972408	2.485531	-3.173323
36.H	5.166511	0.244260	-2.642878
37.H	3.686716	-3.469255	-2.646260
38.H	-2.038901	-1.084180	-2.506335
39.H	3.926133	3.448165	-2.027642
40.H	4.902965	-2.331514	-2.024316
41.H	0.140372	2.090535	-2.175332
42.H	-2.497907	1.573748	-1.976065
43.H	2.174431	3.282964	-1.801683
44.H	5.882336	1.410838	-1.505085
45.H	4.730738	-3.943527	-1.289561
46.H	-0.207942	-2.713122	-1.373265

47.H	5.519971	-0.242773	-0.970821
48.H	1.252981	-4.366924	-0.901845
49.H	-3.806852	-0.381466	-0.604610
50.H	2.515388	-4.796817	0.263905
51.H	1.033026	2.795226	0.258336
52.H	-3.230440	1.971106	0.586223
53.H	4.498067	2.547567	0.779772
54.H	1.144630	-3.801132	0.783003
55.H	4.644717	-1.331447	0.986407
56.H	-5.281717	-3.264838	1.265296
57.H	2.798033	2.236817	1.202703
58.H	-0.791537	-2.932588	1.235906
59.H	3.997875	0.957215	1.402050
60.H	4.285229	-2.976937	1.552877
61.H	-5.415334	-0.179047	1.574141
62.H	-1.628648	5.575456	1.774739
63.H	3.156035	-1.644247	1.906900
64.H	-4.003934	-4.192993	2.078131
65.H	-3.088976	4.697253	2.278367
66.H	1.215571	4.365276	2.381153
67.H	-4.203168	0.699449	2.539153
68.H	-5.300920	-3.442501	3.038306
69.H	1.354254	2.768943	3.162831
70.H	-2.035619	5.439475	3.504097
71.H	-5.410501	-0.325811	3.341009
72.H	-1.852660	-2.754711	3.810616
73.H	-2.871849	1.988613	3.788276
74.H	0.803547	4.178279	4.097529
75.H	-1.933274	-1.003542	4.074431
76.H	-1.271765	1.283580	4.131592
77.H	-3.177811	-2.072871	4.776844
78.H	-1.801449	2.764595	4.968905
79.Cl	0.417842	-0.212453	2.282662
80.Hf	-0.700175	0.063770	0.162401
81.Si	1.464145	-0.808560	-3.614323
82.Si	1.715540	-0.485295	-1.243109
83.Si	3.437521	1.197894	-1.041254
84.Si	2.898860	-2.454724	-0.465632
85.Si	-3.602916	-1.735951	2.346810
86.Si	-1.038636	3.300268	2.666616

Energy: –459.09828473 eV

Table S6. Final coordinates and energy from a single point energy calculation on geometry optimised **3**.

1.C	1.328211	1.170717	-4.933822
2.C	3.910360	1.163818	-3.207842
3.C	1.738831	3.272081	-2.779774
4.C	-3.598659	-1.166099	-2.668702
5.C	2.468328	-4.560441	-2.273711
6.C	-2.292219	2.725988	-2.311838
7.C	-0.063612	-0.378809	-2.128703
8.C	1.277173	0.118368	-2.089675
9.C	-0.093351	-1.680375	-1.553153
10.C	2.045705	-0.908182	-1.431086
11.C	-4.983858	2.553445	-0.935712
12.C	1.222176	-2.042605	-1.135099
13.C	-5.576293	-1.169037	-0.368911
14.C	-3.214329	-3.079315	-0.285688
15.C	0.394326	-4.843141	-0.027149
16.C	-2.672688	3.958041	0.468634
17.C	3.234032	-3.729946	0.569298
18.C	2.106926	1.862647	0.488095
19.C	0.807262	2.426579	0.460464
20.C	5.041170	-0.100583	1.012825
21.C	2.277564	1.118546	1.700526
22.C	0.153006	2.067620	1.676210
23.C	-5.060289	1.101100	2.673629
24.C	1.050249	1.277233	2.429651
25.C	4.748301	1.982509	3.240367
26.C	-3.505826	-1.512135	3.147048
27.C	3.625409	-0.846623	3.691451
28.C	-2.324747	1.167916	3.955890
29.H	1.797249	1.847446	-5.665434
30.H	1.484666	0.135942	-5.272741
31.H	0.244976	1.366511	-4.947264
32.H	4.407021	1.886556	-3.873691
33.H	4.160117	0.153032	-3.562703
34.H	2.199613	3.881762	-3.573914
35.H	-4.292934	-1.894968	-3.118507
36.H	1.684970	-4.607202	-3.046023
37.H	-3.885302	-0.169622	-3.036671
38.H	-2.631044	1.978732	-3.045666
39.H	-2.593954	-1.390718	-3.053746
40.H	-2.555053	3.720401	-2.707623
41.H	0.669899	3.528467	-2.759841
42.H	3.311748	-3.985940	-2.686947
43.H	-0.918124	0.132995	-2.559787
44.H	4.351635	1.284488	-2.206976
45.H	2.820198	-5.588442	-2.088134
46.H	-1.196762	2.662566	-2.256944

47.H	2.184699	3.585609	-1.826363
48.H	-5.313802	1.805948	-1.671947
49.H	-5.224063	3.549744	-1.342891
50.H	-0.978265	-2.299777	-1.450134
51.H	-6.117843	-1.926775	-0.959809
52.H	3.120681	-0.864593	-1.274359
53.H	-3.195447	-3.730169	-1.174713
54.H	-6.000244	-0.184774	-0.612860
55.H	-0.427876	-4.907894	-0.755149
56.H	-5.577099	2.410798	-0.021090
57.H	2.859206	1.975687	-0.285853
58.H	0.385266	3.030949	-0.335419
59.H	-3.088743	4.869924	0.009841
60.H	0.749498	-5.868754	0.161224
61.H	4.101653	-3.183734	0.169475
62.H	5.176365	0.675589	0.244670
63.H	-3.954008	-3.503088	0.410587
64.H	-2.235346	-3.143193	0.208309
65.H	-5.780679	-1.370788	0.692581
66.H	-1.589524	4.109078	0.577250
67.H	4.643836	-1.001234	0.525557
68.H	3.572015	-4.752757	0.798923
69.H	-0.010728	-4.442109	0.911754
70.H	6.039848	-0.346502	1.405926
71.H	-3.099828	3.874545	1.479249
72.H	2.928202	-3.254475	1.510684
73.H	-5.769289	0.648894	1.965527
74.H	-0.842066	2.369085	1.977074
75.H	-5.018363	2.180738	2.465130
76.H	4.930911	2.795831	2.521570
77.H	-4.196200	-2.078854	2.506201
78.H	-2.533599	-2.026484	3.130728
79.H	3.058589	-1.691430	3.276006
80.H	5.716782	1.694443	3.680068
81.H	-5.470429	0.975046	3.689607
82.H	0.836126	0.835985	3.397329
83.H	-2.217146	2.250494	3.792909
84.H	4.118020	2.387612	4.046626
85.H	4.584788	-1.227394	4.075164
86.H	-3.895521	-1.551028	4.177602
87.H	-1.321813	0.734219	4.070031
88.H	3.057250	-0.456882	4.549273
89.H	-2.853822	1.034362	4.913874
90.Cl	0.268350	-1.803081	2.026656
91.Hf	0.461883	-0.070169	0.344448
92.Si	2.038899	1.450331	-3.204854
93.Si	-3.711629	-1.288506	-0.761616
94.Si	1.813240	-3.785574	-0.674566
95.Si	-3.108802	2.440402	-0.607677
96.Si	-2.369060	0.370829	0.365259

97.Si	3.915247	0.501442	2.408051
98.Si	-3.330385	0.297980	2.585416

Energy: −520.51682243 eV

Table S7. Final coordinates and energy from a single point energy calculation on geometry optimised **4**.

1.C	1.072472	-0.969978	-5.740721
2.C	-2.277325	0.841614	-5.427676
3.C	1.044678	2.789500	-5.137029
4.C	-2.530948	-1.792519	-3.880217
5.C	3.249829	-0.410983	-3.681454
6.C	1.380739	-2.824787	-3.391544
7.C	-0.809784	3.883626	-2.991873
8.C	-3.631000	0.816893	-2.729397
9.C	2.128996	3.230591	-2.348690
10.C	-4.493272	-2.414145	0.042592
11.C	-3.269680	3.645339	0.270379
12.C	2.624390	-1.379695	0.122243
13.C	2.782491	0.025903	0.214979
14.C	2.110703	-4.911129	0.586517
15.C	-0.287524	4.131362	0.954687
16.C	3.514645	3.405948	1.244660
17.C	-2.633665	0.476559	1.165762
18.C	2.249496	-1.898314	1.405440
19.C	2.517845	0.439138	1.563972
20.C	-1.733104	1.436644	1.734527
21.C	-2.539885	-0.775162	1.847945
22.C	2.174357	-0.760093	2.268215
23.C	-3.100990	-3.779391	2.468439
24.C	3.963906	-3.970616	2.842728
25.C	-5.251355	-1.641038	2.910486
26.C	-1.051227	0.742935	2.787782
27.C	4.895182	1.465270	3.145950
28.C	-1.531531	-0.589017	2.850356
29.C	-2.363037	4.048319	3.191384
30.C	0.937726	-3.940216	3.318381
31.C	2.123490	2.520472	3.878309
32.H	1.829407	-1.584500	-6.256628
33.H	1.102750	0.039139	-6.174725
34.H	-1.495842	0.470372	-6.105895
35.H	-3.256248	0.591965	-5.870298
36.H	0.087150	-1.404527	-5.964281
37.H	0.219125	2.495000	-5.800486
38.H	-2.200577	1.938466	-5.392640
39.H	1.290292	3.843314	-5.351832
40.H	1.923574	2.184520	-5.403148
41.H	-1.812560	-2.283945	-4.552582
42.H	3.905685	-1.056182	-4.288868
43.H	-3.538194	-1.936688	-4.304087
44.H	3.390077	0.622890	-4.028993
45.H	2.109660	-3.380232	-4.005068
46.H	-1.695961	3.648832	-3.600036

47.H	0.385702	-3.252597	-3.582531
48.H	-4.564260	0.663483	-3.296011
49.H	-0.473251	4.895582	-3.271723
50.H	-2.490097	-2.314065	-2.912941
51.H	3.604236	-0.464692	-2.642086
52.H	-3.509382	1.899910	-2.578888
53.H	3.008251	2.615361	-2.594231
54.H	2.364928	4.272206	-2.622322
55.H	1.609445	-3.014937	-2.334183
56.H	-1.127812	3.916797	-1.940797
57.H	-3.759640	0.347421	-1.743917
58.H	1.992924	3.193436	-1.259044
59.H	2.765192	-1.968785	-0.781102
60.H	-3.077318	3.165565	-0.699659
61.H	-3.717596	-2.778837	-0.645002
62.H	3.083771	0.680537	-0.599310
63.H	-4.902221	-1.478186	-0.367351
64.H	2.855613	-4.711803	-0.199367
65.H	-5.312611	-3.150513	0.055415
66.H	-3.370163	4.727262	0.092206
67.H	0.108751	3.690186	0.029135
68.H	1.113334	-4.866727	0.126910
69.H	4.130091	3.093742	0.388247
70.H	-3.332726	0.695241	0.361192
71.H	-4.237107	3.276553	0.642614
72.H	-0.453034	5.203913	0.766719
73.H	2.280860	-5.938187	0.947367
74.H	2.588616	3.845923	0.851314
75.H	4.065326	4.202447	1.770414
76.H	0.488084	4.051074	1.729374
77.H	-2.297146	-4.167458	1.827423
78.H	4.788831	-3.843457	2.125001
79.H	5.591778	1.173943	2.344527
80.H	-5.718527	-0.710601	2.552814
81.H	-3.888870	-4.547144	2.526370
82.H	-6.029798	-2.421086	2.937375
83.H	-0.069587	-3.810357	2.897136
84.H	-2.494581	5.139202	3.107937
85.H	4.025373	-4.992660	3.250917
86.H	1.957272	-0.808260	3.334540
87.H	-2.701681	-3.645266	3.485245
88.H	-3.304882	3.619572	3.565367
89.H	4.130043	-3.264927	3.670888
90.H	4.806229	0.611994	3.835525
91.H	5.347436	2.304742	3.698977
92.H	-0.308741	1.173356	3.455125
93.H	-1.209104	-1.340681	3.569489
94.H	1.149568	2.910111	3.552363
95.H	0.993881	-4.953710	3.745357
96.H	-4.910194	-1.467991	3.942732

97.H	-1.587489	3.862828	3.949863
98.H	1.057112	-3.228653	4.150089
99.H	2.627891	3.321368	4.441446
100.H	1.941369	1.694530	4.582608
101.Cl	-0.781905	-2.873954	-0.354069
102.Si	1.433528	-0.977721	-3.866117
103.Si	-2.159422	0.065443	-3.687135
104.Si	0.587680	2.619192	-3.292432
105.Si	-0.053850	0.421154	-2.569811
106.Si	-1.892380	3.307657	1.514791
107.Si	-3.819322	-2.170866	1.787649
108.Si	2.284942	-3.691385	2.012545
109.Si	3.215607	1.970878	2.430757
110.Th	-0.019452	-0.501569	0.468878

Energy: −582.64635284 eV

Table S8. Final coordinates and energy from a single point energy calculation on geometry optimised **5**.

1.C	1.773848	-1.064623	-5.305416
2.C	4.388316	-2.080998	-4.019905
3.C	3.672425	0.874641	-3.842786
4.C	-0.188900	1.037386	-2.640567
5.C	2.034778	-1.341983	-2.248837
6.C	0.694379	-1.860924	-2.141038
7.C	0.590354	2.073915	-2.079570
8.C	-1.283615	0.792982	-1.768347
9.C	1.663205	5.031852	-1.248935
10.C	-3.025651	-2.674047	-1.218890
11.C	2.659208	-1.637472	-0.995318
12.C	0.506148	-2.419837	-0.856248
13.C	-0.039171	2.541099	-0.868611
14.C	-1.197949	1.724420	-0.696603
15.C	-1.239423	5.271619	-0.193056
16.C	-4.451976	-0.219603	-0.097892
17.C	1.730814	-2.272401	-0.138237
18.C	2.858443	1.267032	-0.024882
19.C	2.573281	0.569662	1.174477
20.C	-4.366556	-2.830707	1.469156
21.C	1.007425	4.299998	1.641518
22.C	-0.531039	-4.401727	1.890933
23.C	1.380890	0.688708	1.884612
24.C	-2.553501	2.399275	2.501283
25.C	1.081439	-2.523074	3.684968
26.C	-3.825054	0.047513	3.870830
27.C	-1.812635	-3.081135	4.298241
28.C	-0.976672	0.866470	4.589693
29.H	2.328822	-0.931237	-6.247259
30.H	1.319410	-2.066027	-5.327289
31.H	0.960802	-0.323851	-5.303086
32.H	4.944262	-1.890992	-4.951867
33.H	4.336972	0.993248	-4.713282
34.H	4.038880	-3.124138	-4.035617
35.H	2.896099	1.648670	-3.909556
36.H	5.097772	-1.981915	-3.183956
37.H	0.013857	0.519524	-3.571110
38.H	4.268085	1.073030	-2.939916
39.H	-0.050509	-1.854640	-2.931344
40.H	1.483465	2.500158	-2.523849
41.H	1.317855	5.101552	-2.291163
42.H	-2.065714	0.056120	-1.910936
43.H	-2.421243	-2.147178	-1.970528
44.H	-4.003539	-2.897586	-1.675873
45.H	2.606413	4.464924	-1.242233
46.H	-1.640159	5.332617	-1.215997

47.H	1.893087	6.051589	-0.902622
48.H	-2.535636	-3.638911	-1.017985
49.H	3.689041	-1.404543	-0.739226
50.H	-3.983976	0.485704	-0.799021
51.H	-5.362206	-0.613005	-0.579894
52.H	-0.388472	-2.912180	-0.499986
53.H	3.796658	1.054650	-0.535245
54.H	2.525954	2.299549	-0.113647
55.H	-1.053094	6.296673	0.165555
56.H	-1.924012	1.812004	0.102447
57.H	-2.019145	4.831174	0.446920
58.H	-4.769563	0.355978	0.783996
59.H	-5.276586	-3.114126	0.914070
60.H	1.916035	-2.607970	0.876226
61.H	0.270901	-4.427084	1.140301
62.H	-1.476676	-4.632152	1.377398
63.H	3.196806	-0.295825	1.415544
64.H	1.948583	3.743999	1.767027
65.H	-3.832037	-3.758215	1.723329
66.H	0.814750	1.619869	1.848269
67.H	1.206986	5.352668	1.900550
68.H	-3.336808	2.415995	1.728638
69.H	-1.669970	2.913096	2.100060
70.H	-4.680720	-2.355533	2.409179
71.H	0.287039	3.917800	2.379635
72.H	-0.339837	-5.218718	2.606053
73.H	1.205418	0.059061	2.750081
74.H	-4.630476	0.014218	3.122560
75.H	1.906446	-2.353241	2.978349
76.H	-2.922153	2.996876	3.350897
77.H	-2.836988	-3.292826	3.960134
78.H	-3.754510	-0.946476	4.333595
79.H	1.321937	-3.431635	4.261134
80.H	1.075915	-1.679950	4.392387
81.H	-0.040388	1.359527	4.291521
82.H	-4.129979	0.762652	4.653343
83.H	-1.454372	-3.961147	4.858107
84.H	-1.857504	-2.237241	5.001762
85.H	-0.712885	-0.087716	5.068721
86.H	-1.457633	1.498315	5.354328
87.Hf	0.773515	0.061631	-0.511263
88.Si	2.945820	-0.872413	-3.833056
89.Si	0.348050	4.246396	-0.133041
90.Si	-3.301499	-1.668754	0.390469
91.Si	-1.262670	-0.875152	1.433800
92.Si	-0.624308	-2.742786	2.841578
93.Si	-2.169208	0.626704	3.114540

Energy: –498.91735822 eV

Table S9. Final coordinates and energy from a single point energy calculation on geometry optimised **6**.

1.C	-2.975559	0.251366	-4.845927
2.C	-3.712385	2.090496	-2.505339
3.C	-1.139049	0.305214	-2.424294
4.C	-0.388618	-0.920657	-2.386559
5.C	-3.949638	-0.985038	-2.205854
6.C	-0.161660	1.356734	-2.370232
7.C	0.989136	-0.628924	-2.290557
8.C	1.131311	0.787403	-2.269579
9.C	5.007330	-1.248070	-1.373524
10.C	4.947032	-2.644629	-1.302276
11.C	0.011109	5.091848	-0.744878
12.C	4.073969	-0.464221	-0.692814
13.C	3.942271	-3.237997	-0.529198
14.C	2.643095	3.681880	0.045252
15.C	3.041643	-1.039927	0.078645
16.C	3.009278	-2.449091	0.147664
17.C	-1.480150	2.270532	0.402069
18.C	-0.960746	-1.894353	0.402314
19.C	2.032458	-0.194526	0.771544
20.C	-0.173851	2.531311	0.934592
21.C	-2.077838	1.208088	1.123434
22.C	-1.413499	-2.298769	1.760835
23.C	0.959077	5.054510	2.196675
24.C	-2.785760	-2.320507	2.090750
25.C	-0.012592	1.613612	2.030358
26.C	-1.169394	0.812257	2.146882
27.C	-0.508923	-2.666332	2.780019
28.C	-3.230765	-2.671917	3.366499
29.C	-0.948761	-3.020484	4.057798
30.C	-2.314463	-3.022085	4.364841
31.H	-4.007126	0.290715	-5.230994
32.H	-2.402234	1.058232	-5.327705
33.H	-2.530336	-0.703749	-5.163432
34.H	-4.692704	2.189946	-2.997493
35.H	-3.092581	2.933360	-2.846184
36.H	-4.985189	-0.959866	-2.579932
37.H	-3.529872	-1.966714	-2.472215
38.H	-0.810633	-1.920366	-2.430008
39.H	5.793051	-0.765103	-1.957339
40.H	-0.376923	2.420449	-2.404727
41.H	5.677781	-3.259162	-1.829051
42.H	1.799410	-1.350344	-2.224827
43.H	2.071687	1.326114	-2.198918
44.H	-0.023924	4.573286	-1.714398
45.H	-3.874668	2.199433	-1.423845
46.H	0.546559	6.041354	-0.900773

47.H	-3.986367	-0.923963	-1.108576
48.H	2.677061	3.145077	-0.913225
49.H	4.147219	0.624455	-0.747988
50.H	3.887184	-4.324992	-0.447991
51.H	-1.021090	5.337217	-0.453298
52.H	3.205353	4.621536	-0.075901
53.H	-1.937194	2.803174	-0.425381
54.H	-1.762158	-2.094634	-0.323220
55.H	-0.097578	-2.508408	0.083745
56.H	3.175530	3.073901	0.791676
57.H	2.238123	-2.934892	0.749417
58.H	-3.060301	0.779641	0.946244
59.H	2.467232	0.787963	1.007569
60.H	-3.517372	-2.066545	1.319967
61.H	1.503321	6.001432	2.052445
62.H	1.750467	-0.649242	1.738529
63.H	-0.047939	5.291358	2.572151
64.H	1.479590	4.485594	2.982548
65.H	0.561681	-2.676414	2.563794
66.H	0.857633	1.548063	2.677197
67.H	-1.335698	0.024198	2.875774
68.H	-4.301179	-2.683137	3.579868
69.H	-0.219091	-3.302489	4.819051
70.H	-2.659636	-3.302787	5.360577
71.Hf	-0.085541	0.197521	-0.100517
72.Si	-2.940871	0.424328	-2.962500
73.Si	0.871310	4.062666	0.589348

Energy: –429.18895328 eV
